# Synthesis, crystal structure, and characterization of a new crystalline berberine–2-sulfobenzoate salt, integrating vibrational spectroscopy and quantum chemical investigation

**DOI:** 10.3389/fchem.2026.1971429

**Published:** 2026-10-21

**Authors:** Anubha Srivastava, Preeti Prajapati, Manishkumar R. Shimpi, Poonam Tandon

**Affiliations:** 1 Department of Physics, University of Lucknow, Lucknow, India; 2 Chemistry of Interfaces, Luleå University of Technology, Luleå, Sweden; 3 Department of Physics, Deen Dayal Upadhyaya Gorakhpur University, Gorakhpur, India

**Keywords:** berberine–2-sulfobenzoate salt, synthesis, crystal structure, PXRD, FT–Raman

## Abstract

A hydrothermal crystallization screen was conducted to expand the solid-form landscape of berberine. Hydrolysis of saccharin under the applied conditions produced 2-sulfobenzoic acid, leading to the formation of a new crystalline berberine–2-sulfobenzoate salt (BER–SBA). Single-crystal X-ray diffraction established a 1:1 salt structure stabilized by C–H•••O interactions between berberine and sulfobenzoate ions and O–H•••O interactions between neighboring sulfobenzoate units. Agreement between the experimental and simulated powder X-ray diffraction patterns confirmed the phase identity of the bulk material. BER–SBA exhibited a higher differential scanning calorimeter onset temperature than the investigated berberine chloride sample (257.6 °C ± 0.5 °C) and underwent single-step decomposition after melting, indicating greater apparent thermal robustness up to the melting transition. FT–Raman spectroscopy and density functional theory calculations using a 1:2 molecular cluster provided complementary insight into the local intermolecular interactions. The Quantum Theory of Atoms in Molecules (QTAIM), reduced-density-gradient, natural bond orbital, and molecular electrostatic potential analyses supported the hydrogen-bonding and charge-transfer network associated with intermolecular stabilization within the modeled assembly. Frontier-orbital analysis further indicated that salt formation altered the calculated electronic properties of the system. Furthermore, molecular docking demonstrated favorable binding affinities against 3-hydroxy-3-methylglutaryl-coenzyme A (HMG-CoA) reductase, mitogen-activated protein kinase (MAPK), and Nrf2 targets, respectively. Overall, the crystallographic, thermal, spectroscopic, and computational results establish BER–SBA as a distinct crystalline berberine salt and provide a molecular-level understanding of its solid-state stabilization. These findings support BER–SBA as a promising solid form for further pharmaceutical evaluation, particularly with respect to hygroscopicity and formulation performance.

## 1 Introduction

Multicomponent crystallization is an established strategy in pharmaceutical solid-state chemistry for modifying the physicochemical and material properties of active pharmaceutical ingredients (APIs) (Schultheiss and Newman, 2009; Xia et al., 2022). Through the formation of salts, cocrystals, solvates, and related crystalline phases, this approach can influence properties such as solubility, dissolution behavior, hygroscopicity, thermal stability, mechanical performance, and bioavailability (Panzade and Shendarkar, 2020; Kavanagh et al., 2019). The identification and characterization of new multicomponent crystalline forms are therefore important components of pharmaceutical solid-form development (Hasa et al., 2020; Reddy et al., 2010; Ahmed et al., 2017).

Berberine (Ber) is an isoquinoline alkaloid found in the roots and stem bark of several medicinal plants, including *Berberis vulgaris*, *Berberis aristata*, and *Berberis aquifolium* (Zuo et al., 2006). It has been investigated for a wide range of biological activities, including lipid- and cholesterol-lowering (Doggrell, 2005), antitumor (Jantova et al., 2003; Kettmann et al., 2004), antihyperglycemic (Pan et al., 2003), antimalarial (Tran et al., 2003), antiarrhythmic (Sanchez-Chapula, 1996; Tsai and Tsai, 2004), antihypertensive (Ko et al., 2009), antifungal (Lu et al., 2006), anti-inflammatory (Kupeli et al., 2002; Yesilada et al., 2002), and antioxidant effects (Misik et al., 1995). Ber is commonly encountered as a yellow crystalline material with limited aqueous solubility, although its physicochemical properties depend strongly on its ionic form and solid-state structure (Dostál et al., 2004). Berberine chloride (BerCl) is one of its most widely used salt forms and has been incorporated into oral and topical formulations (Battu et al., 2010; Patel et al., 2010).

Previous studies have demonstrated that the solid-state behavior of Ber is strongly influenced by the counterion and hydration state. Kariuki and Jones (1995) reported several inorganic Ber salts and their hydrates, although their thermal behavior was not systematically evaluated. Tong et al. (2010) subsequently showed that BerCl can undergo temperature- and moisture-dependent transformations among tetrahydrate, dihydrate, and anhydrous forms. Such interconversions may complicate material handling, processing, storage, and solid-form control. An organic Ber palmitate salt with a layered crystal structure has also been reported, but its thermal behavior was not evaluated by differential scanning calorimetry or thermogravimetric (TG) analysis (Chahine et al., 2025). More recently, Ber–saccharin (BbSac) and Ber–acesulfame salts (BbAs) were prepared and characterized, demonstrating the ability of organic counterions to generate distinct crystalline Ber phases (Wang et al., 2016).

These findings indicate that alternative counterions may provide access to Ber salts with different crystal-packing arrangements and thermal properties. However, the relationships among counterion selection, intermolecular interactions, crystal structure, and the resulting solid-state behavior remain insufficiently understood. This knowledge gap is particularly relevant for BerCl because of its hydrate-dependent phase transformations and sensitivity to temperature and moisture (Tong et al., 2010). A combined experimental and computational approach can help establish how the intermolecular interaction network of a new salt influences its structural and thermal characteristics.

In the present study, hydrothermal crystallization screening of BerCl was performed with a range of organic compounds to expand the solid-form landscape of Ber. Screening in the presence of saccharin produced a new crystalline phase. Under the applied hydrothermal conditions, saccharin underwent hydrolysis to form 2-sulfobenzoic acid, followed by the formation of a berberine–2-sulfobenzoate salt, designated BER–SBA. Single-crystal X-ray diffraction established that the isolated material is a 1:1 ionic solid composed of berberine cations and 2-sulfobenzoate anions.

BER–SBA was characterized by single-crystal X-ray diffraction, powder X-ray diffraction, differential scanning calorimetry, thermogravimetric analysis, and FT–Raman spectroscopy. Its thermal behavior was compared with that of the investigated BerCl material to determine whether the formation of the new salt altered the thermal profile of Ber. Density functional theory calculations were used to complement the experimental findings and provide molecular-level insight into the local intermolecular interactions, charge-transfer effects, and electronic characteristics of the BER–SBA assembly.

The experimental crystal structure has a 1:1 stoichiometric ratio of Ber to SBA. In contrast, a 1:2 BER–SBA molecular cluster was selected for the computational analysis to represent key local contacts involving one Ber ion and two neighboring SBA units within the crystal environment. The model was not intended to redefine the experimentally established stoichiometry. Instead, it was used to examine the principal O–H•••O interactions between neighboring SBA units and the C–H•••O contacts between Ber and SBA observed within the local interaction network.

The computational investigation included vibrational assignments, the Quantum Theory of Atoms in Molecules (QTAIM), reduced-density-gradient and noncovalent-interaction analyses, natural bond orbital analysis, molecular electrostatic potential mapping, frontier molecular orbital analysis, and global reactivity descriptors. These methods were applied to investigate the nature and relative importance of the interactions contributing to local stabilization of the modeled BER–SBA assembly. Molecular docking of Ber against selected biological targets was additionally performed as an exploratory analysis of its potential ligand–protein interactions. Overall, this study integrates crystallographic, thermal, spectroscopic, and computational methods to establish BER–SBA as a distinct crystalline Ber salt and to clarify the molecular interactions associated with its solid-state structure and thermal behavior.

## 2 Method and simulations

### 2.1 Experimental details

#### 2.1.1 Materials

BerCl was purchased from Sichuan Xieli Pharmaceutical, Co., Ltd. All chemicals and solvents (Purity >99%) were purchased from Sigma Aldrich or Acros Organics and were used without further purification.

#### 2.1.2 Hydrothermal screening experiments

Hydrothermal screening of BerCl was performed with various safe additive chemicals such as benzoic acid, fumaric acid, saccharin, adipic acid, succinic acid, and ketoglutaric acid. In a typical screening, equimolar amounts of (0.1 mmol) BerCl and the corresponding organic moiety were dissolved in 10 mL of water. The mixture was stirred for 15 min at room temperature and then sealed in a 25 mL Teflon-lined autoclave and heated at 120 °C for 10 h, followed by gradual cooling to room temperature. Plate- and block-type yellow crystals were obtained from the autoclave containing the fumaric acid and saccharin system. In this study, we focus on the Ber–saccharin system. Note that under the hydrothermal conditions employed, saccharin underwent hydrolysis to form 2-sulfobenzoic acid. Subsequent solid-state characterization, including single-crystal X-ray diffraction, revealed that the isolated crystalline material was the BER–SBA salt. Therefore, the present study focuses on the structural and physicochemical characterization of the BER–SBA system.

#### 2.1.3 Powder X-ray diffraction (PXRD)

Powder X-ray diffraction (PXRD) patterns for the samples were collected using an Empyrean X-ray diffractometer (PANalytical, Netherlands) equipped with a PIXel3D detector and a monochromatic Cu Kα radiation X-Ray tube (1.54056). The tube voltage and amperage were set at 45 kV and 40 mA, respectively. Samples were prepared for analysis by pressing a thin layer of the sample onto a metal sample holder. Instrument calibration was performed using a silicon reference standard. Each sample was scanned 2θ range of 5^o^ to 40^o^, increasing at a step size of 0.02^o^ 2θ. The data were process using High Score Plus Version 3.0 software (PANalytical, Netherlands).

#### 2.1.4 Differential scanning calorimetry (DSC)

Thermal analyses of these samples were performed on a TA instruments differential scanning calorimeter Q1000 V9.9 Build 303. The crystals (1–3 mg) were placed in aluminum crucibles and were scanned at 10 °C/min in the range of 25 °C–300 °C under a dry nitrogen atmosphere (flow rate 50 mL/min). The data were managed using TA universal analysis software. Indium was used as the calibration standard.

#### 2.1.5 Thermogravimetric (TG) analysis

TG analyses were performed using a TA Instruments TGA Q5000 V3.8 Build 256 thermogravimetric analyzer. The samples (approximately 5 mg) were placed into a platinum sample pan and inserted into the TG furnace. The samples were heated from 25 ^°^C to 400 °C at the rate of 10 ^°^C/min under nitrogen purge at a flow rate of 50 mL/min. The data were managed using TA universal analysis software.

#### 2.1.6 FT–Raman spectrometry

The FT–Raman spectrometer” MultiRAM” uses fluorescence-free excitation laser excitation at 1,064 nm and is equipped with a liquid nitrogen cooled D418-T/R27 Germanium detector. The spectra of the powered samples were obtained by averaging 64 scans with an optical resolution of 4 cm^-1^. The resulting interferograms were Fourier-transformed using the Mertz phase correction mode, a Blackman–Harris 3-term apodization function, and a zero-filling factor of 2. All spectra were recorded using the double-side forward–backward acquisition model.

#### 2.1.7 Single-crystal X-ray diffraction

The single-crystal X-ray diffraction data of the crystals were collected on a Bruker Nonius Kappa CCD. The dataset for compounds was collected at room temperature (293K). The crystals were quite stable, and hence, no extraordinary precautions were necessitated for the smooth collection of intensity data. All the datasets were collected using Mo Kα radiation (λ = 0.71073 Å), and crystal structures were solved using direct methods with SIR-92 and refined by full-matrix least-squares refinement on F2 with anisotropic displacement parameters for non-H atoms using SHELXL-2013. Hydrogen atoms were placed on their expected chemical positions using the HFIX command. All the non-hydrogen atoms were refined anisotropically. Structure solution, refinement, and generation of publication materials were performed using shelXLe, V623 software (Hubschle et al., 2011). Supplementary Tables S1, S2 present the pertinent crystallographic data and the hydrogen-bond parameters, respectively. All the intermolecular interactions were computed using PLATON (Spek, 2003). All packing diagrams are generated using Diamond software (Crystal-Impact, 2008).

### 2.2 Quantum chemical computational details

The molecular characteristics and optimization of studied compounds were analyzed using DFT (Hohenberg and Kohn, 1964) with Gaussian 16 software (Frisch et al., 2016) and 6–311++G(d,p) basis set (Petersson and Allaham, 1991; Petersson et al., 1988), retaining closed-shell Becke–Lee–Yang–Parr hybrid exchange-correlation three-parameter functional (B3LYP) (Becke, 1993; Lee et al., 1988; Perdew and Wang, 1992). The vibrational wavenumbers and assignments of the normal modes were analyzed according to Pulay’s recommendation (Pulay et al., 1979) using the Gar2Ped suite (Martin and Alsenoy, 1995). The calculated wavenumbers were scaled using the wavenumber linear scaling (WLS) procedure by Yoshida et al. (2002) to overcome the harmonicity (Bloino et al., 2015). The optimized geometry was plotted using GaussView 6.0 software (Dennington et al., 2016). The comparisons of PXRD, differential scanning calorimetry (DSC), and FT–Raman spectra were performed using the Origin program (Origin (Pro) Version, 2020). In AIM analysis, the interaction energies were defined using Bader’s Quantum Theory of Atoms in Molecules (Bader, 1985) and the AIMALL program (Keith, 2009). The types of hydrogen bonds have been categorized by a number of definite criterion that has been demarcated in the appropriate literature (Srivastava et al., 2017). The reduced density gradient (RDG) graph was designed using the Multiwfn program (Lu and Chen, 2012). Furthermore, the MEP surface was also plotted using the GaussView 6.0 program (Dennington et al., 2016).

## 3 Results and discussion

### 3.1 Structure investigation

BerCl occurs as a yellowish polycrystalline powder with a bitter taste and no odor. It is well known that BerCl undergoes solid state transformations among an anhydrate and two hydrates depending on relative humidity. XRPD analysis of the BerCl sample was carried out by comparing the experimental diffraction pattern with simulated XRPD patterns generated from reported single-crystal structures of Ber dihydrate and Ber tetrahydrate (see Supplementary Figure S1). The experimental pattern shows a clear overlap with characteristic reflections from both hydrate forms, with no single-phase accounting for all observed peaks. Based on the comparative analysis, the sample is best described as a physical mixture of Ber dihydrate and Ber tetrahydrate, likely arising from partial hydration during preparation or storage. Our main objective was to screen and identify new salts or cocrystals of BerCl. To achieve hydrothermal crystallization, screening experiments were carried out involving BerCl in the presence of various organic molecules. Notably, Wang et al. (2016) previously reported the preparation of a BbSac salt at room temperature via an anion-exchange reaction between berberine chloride and sodium saccharin. Saccharin is known to undergo slow hydrolysis in hot aqueous solutions, as reported in previous studies (Wang et al., 2016). Under the hydrothermal conditions employed in this study, saccharin was hydrolyzed to form 2-sulfobenzoic acid, which subsequently underwent an ion-exchange reaction with BerCl. This process resulted in the formation of the BER–SBA salt, while Cl ions were displaced from the crystal structure and remained in the mother liquor as soluble counterions. Consequently, Cl was not incorporated into the final crystalline phase. Consistent with this interpretation, no Cl atom was detected in the refined single-crystal X-ray structure, and the empirical formula derived from the crystallographic data contains only Ber and 2-sulfobenzoate components, resulting in the formation of a 1:1 salt with Ber, called the BER–SBA salt system.

Yellow block-shaped (Supplementary Figure S2) BER–SBA suitable for crystal structure determination by single-crystal X-ray diffraction (SCXRD) was obtained by hydrothermal crystallization. The SXRD results showed that the BER–SBA salt crystallized in the triclinic space group P-1 and possessed one molecule of the Ber anion and one molecule of the 2-sulfobenzoic acid cation. An asymmetric unit containing Ber and SBA with the ratio 1:1 is shown in Figure 1a. No classic hydrogen bonds can be found in Ber and SBA. Rather, only weak C−H•••O hydrogen bonds (Supplementary Table S2) are present to stabilize these two components (Figure 1b). However, two SBA anions interact through O−H•••O (1.79 Å, 170°) hydrogen bonds, as shown in Figure 1c. The three-dimensional layered architecture extending along the c axis in the crystal structure of the BER–SBA salt is shown in Figure 1d.

**FIGURE 1:**
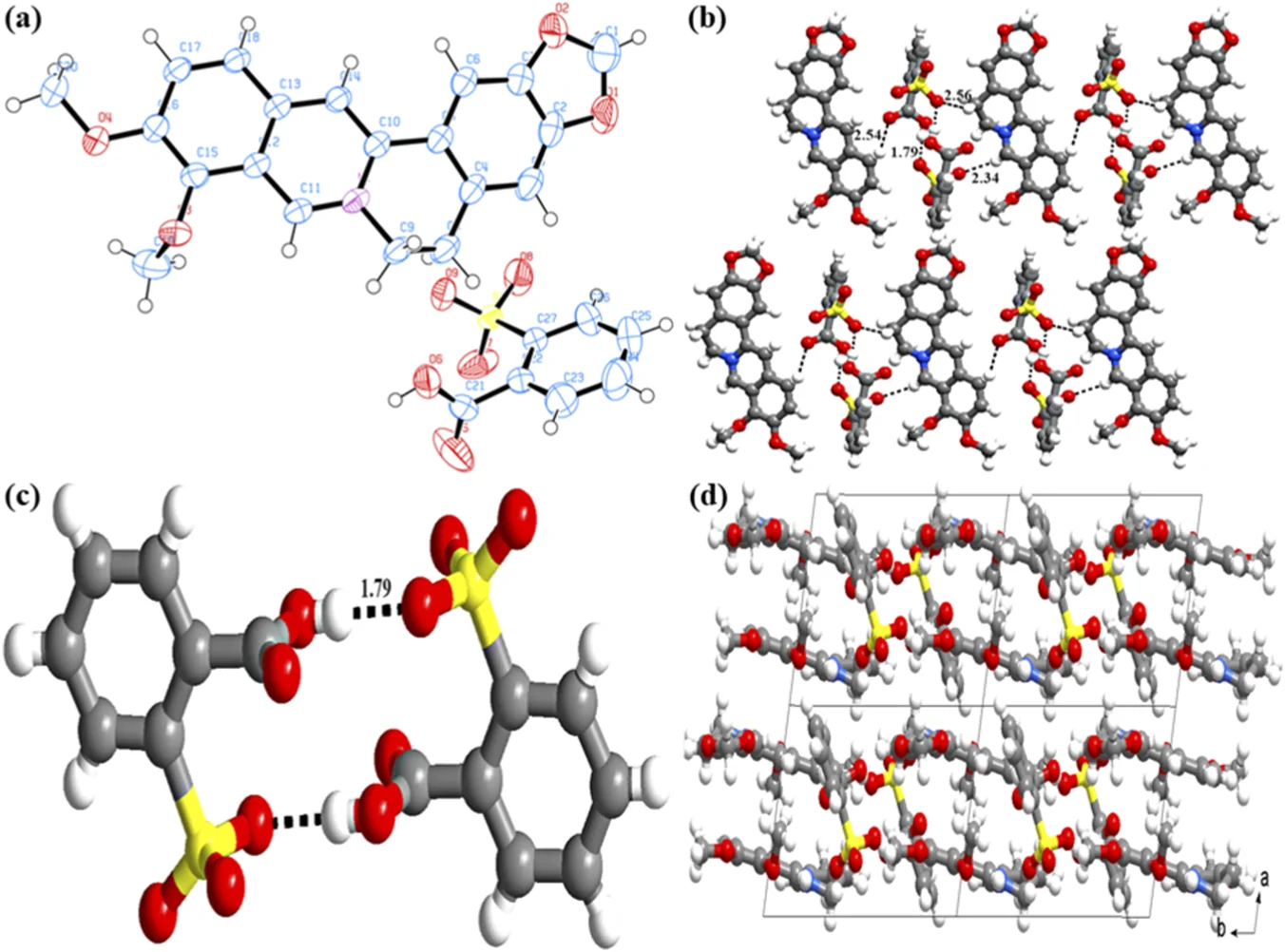
**(a)** ORTEP representation of the BER–SBA salt; **(b)** sheet-like packing formed via C−H•••O hydrogen bonds between Ber and SBA; **(c)** dimeric interaction between SBA anions within the lattice; **(d)** three-dimensional layered architecture extending along the c axis in the crystal structure of the BER–SBA salt.

The crystallographic data for this salt were deposited at CCDC (No. 2529913). Supplementary Table S1 summarizes the crystal parameters, data collection, and refinement details of the salt. Powder X-ray diffractograms of the bulk crystalline material of BER–SBA well matched the simulated PXRD patterns derived from single-crystal structures (Figure 2a). This confirms that the bulk material is the single-phase and pure crystalline salt. The thermal analyses of BerCl and BER–SBA salt is shown in Figure 2b. DSC of BerCl typically shows endothermic signals attributable to the loss of lattice water, while Thermogravimetric analysis (TGA) reveals mass loss corresponding to the loss of water molecules at approximately 90 °C. After that, BerCl shows a melting onset temperature of 176.6 °C, with a peak temperature of 189.6 °C. In contrast, the hydrothermally derived BER–SBA salt exhibits a single endothermic event in the DSC, with an average onset temperature of 257.6 °C ± 0.5 °C, an average peak temperature of 264.2 °C ± 0.5 °C, and an average enthalpy of melting of 132.7 ± 2.1 J/g (errors are sample standard deviations; n = 3). Wang et al. (NIH, 2026) previously developed BbSac and BbAs salts. These salts exhibited single DSC endotherms with onset temperatures of 225 °C and 229 °C and enthalpies of fusion of 112.4 and 56.44 J/g, respectively. Thus, the BER–SBA onset temperature was 32.6 °C higher than that of BbSac and 28.6 °C higher than that of BbAs, while its transition enthalpy exceeded the corresponding values by 20.3 and 76.26 J/g, respectively. The TGA results are in line with the DSC data, with BER–SBA displaying a single-step decomposition immediately after melting rather than multi-stage mass losses. Taken together, the BER–SBA salt exhibits a sharper thermal transition, greater apparent thermal stability up to its melting point, and a simpler, one-step decomposition pattern, as shown in Figure 2b.

**FIGURE 2:**
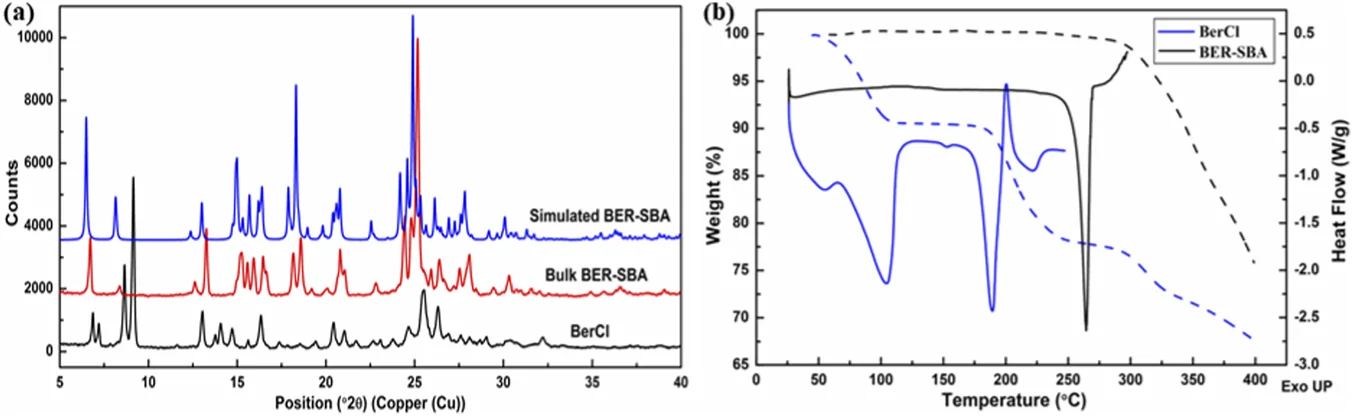
**(a)** PXRD pattern generated using simulated single crystal structure of BER–SBA (top) compared with the PXRD pattern of the bulk crystalline BER–SBA (middle) and BerCl (bottom), **(b)** DSC and TGA thermograms for BerCl and BER–SBA.

#### 3.1.1 Geometry optimization

The initial structures of BerCl and SBA were obtained from Kornilova et al. (2022) and the PubChem Database (NIH, 2026), and their optimized structures are presented in Supplementary Figures S3, S4, respectively. The crystal structure of the BER–SBA salt, along with its hydrogen bonding, and the ground-state optimized structure of the 1:2 model are shown in Figures 1, 3, respectively. The optimized energies of BerCl, SBA, and the BER–SBA salt were calculated as −1,589.1008, −1,044.8238, and −3199.13725792 Hartree, respectively.

**FIGURE 3:**
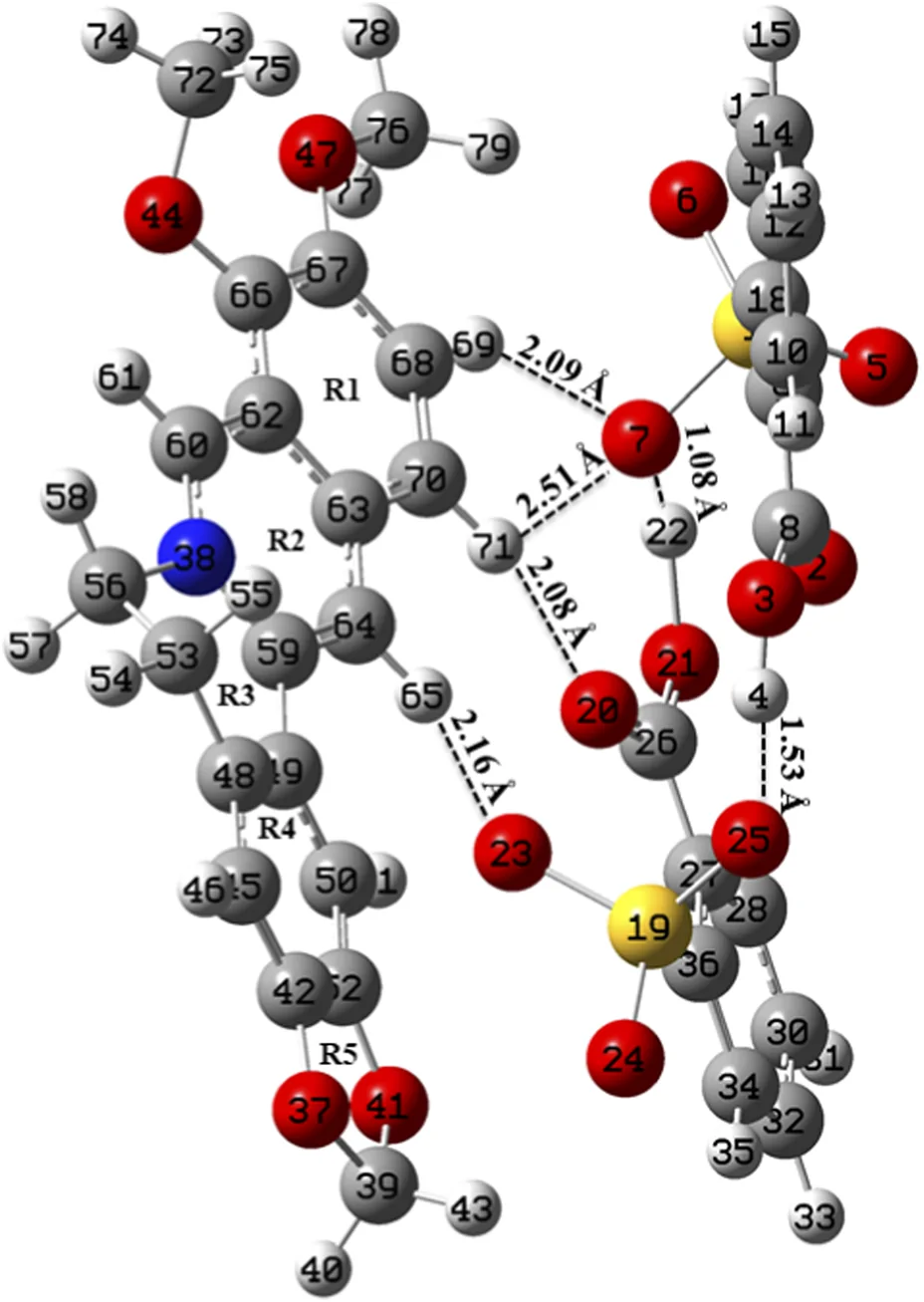
Optimized structure for the 1:2 model of the BER–SBA salt with the atomic numbering scheme adopted in this study.

A comparison between the optimized and experimental values of BerCl, SBA, and the BER–SBA salt is provided in Supplementary Table S3. After comparing the optimized parameter of BerCl with that of the BER–SBA salt, it is noticed that the values do not changes by more than 0.06 Å in the bond length, 5.4° in the bond angle, and 30.5° in the dihedral angle, apart from the bonds having one hydrogen atom. Maximum differences were expected around the (C19–H20) and (C27–H28) bonds of BerCl (Supplementary Figure S3) because of the participation of these groups in intermolecular hydrogen bonding (S1-O7•••H69-C68; C26 = O20•••H71-C70) with SBA in the salt system (Figure 3). However, this was not observed because ring C–H groups are less likely to undergo noticeable shifting than other hydrogen-bonding groups such as N–H or O–H. This is due to the π-system of the ring, which generates a more stable electronic atmosphere for the C–H protons, making them less prone to hydrogen-bonding interactions (Nishio, 2012).

Similarly, a comparison was made between the optimized values of 2Sfa and the BER–SBA salt, and differences of 0.03 Å, 3.9°, and 4.9° were observed in their bond lengths, bond angles, and dihedral angles, respectively, excluding bonds with a hydrogen atom. The prominent changes take place about the bonds (S1 = O; C13 = O6) and (O5-H19; S1 = O) in the SBA molecule (Supplementary Figure S4) due to the formation of intermolecular hydrogen bonds (S1–O7. H69–C68; C26 = O20. H71–C70) and (S1–O7. H22–O21; S19-O25. H4–O3), respectively, with Ber in the salt system, as shown in Figure 3. Among them, the maximum changes were noticed around the respective groups of this (S1–O7. H22–O21; S19–O25. H4–O3) intermolecular hydrogen bond as it is stronger than the other bond.

Moreover, the optimized geometrical parameters of the BER–SBA salt were consistent with their experimental data, resulting that the differences not arises by more than 0.3 Å in the bond length, 9.8° in the bond angle, and 34.2° in the dihedral angle, except the bonds having hydrogen atoms. The comparative examination of observed and simulated values showed good agreement; however, some major differences were observed around the bonds which are involved in the intermolecular interactions with neighboring molecules in the crystal structure, such as (C8 = O2) and few O atoms of the SO_3_ group (Figure 1) because the simulations were performed on only the (1:2) system, although the experiments were performed under the bulk condition.

Equally, the BER–SBA salt system consists of several intermolecular hydrogen bonds between BerCl (API) and SBA (coformer); therefore, binding energy (B.E) was also computed for the present system, which was evaluated as the difference between the total energy of the salt and the sum of individual energies of BerCl and SBA molecules, as shown in the following equation:

$$B.E=E_{BER-SBA}-\left(E_{BerCl}+E_{SBA}\right),$$

where *E*_*BER−SBA*_ = Total energy of salt.

*E*_*BerCl*_ = Energy of API.

*E*_*SBA*_ = Energy of the coformer.

The calculated binding energy was −169.43 kcal mol^-1^ using DFT (Hohenberg and Kohn, 1964) methods. The simulated binding energy was further corrected for basis set superposition error (BSSE) using the counterpoise method proposed by Boys and Bernardi (1970), yielding a value of −162.15 kcal mol^-1^.

### 3.2 Spectroscopic assignments and wavenumber

Vibrational spectroscopy, in conjunction with quantum chemical methodology, is often utilized to assign vibrational modes in molecular systems (Prajapati et al., 2022; Khanum et al., 2022). The studied constituents such as BerCl, SBA, and the BER–SBA salt contain 44, 19, and 79 atoms, respectively, which have generated 126, 51, and 231 (3N-6) vibrational modes, respectively. Generally, DFT simulations produce Raman amplitudes, which cannot be directly interpreted as Raman intensities. Hence, Raman scattering cross section, ∂σj/∂Ω, which are proportional to Raman intensity, may be calculated from the Raman scattering amplitude. Experimentally and theoretically calculated values of vibrational wavenumbers, along with their PED assignments for BerCl, SBA, and the BER–SBA salt, are tabulated in Supplementary Tables S4–S6, respectively. The calculated values were found to be overestimated compared to experimental recorded vibrational frequencies due to the negligence of anharmonicity and the presence of the environment (gaseous phase). For this purpose, the calculated wavenumbers were corrected using the WLS formula [νobs/νcal = (1.0087 − 0.000016× νcal) cm^-1^] proposed by Yoshida et al. (2002) to achieve the best fit with the experimental spectra. Supplementary Figures S5, S6 and Figure 4 show the comparative experimental and calculated Raman spectra of BerCl, SBA, and the BER–SBA salt, respectively. The significant changes in stretching vibrational wavenumbers and the bond lengths of the groups involved in hydrogen bonding of BerCl and SBA molecules are presented in Table 1.

**FIGURE 4:**
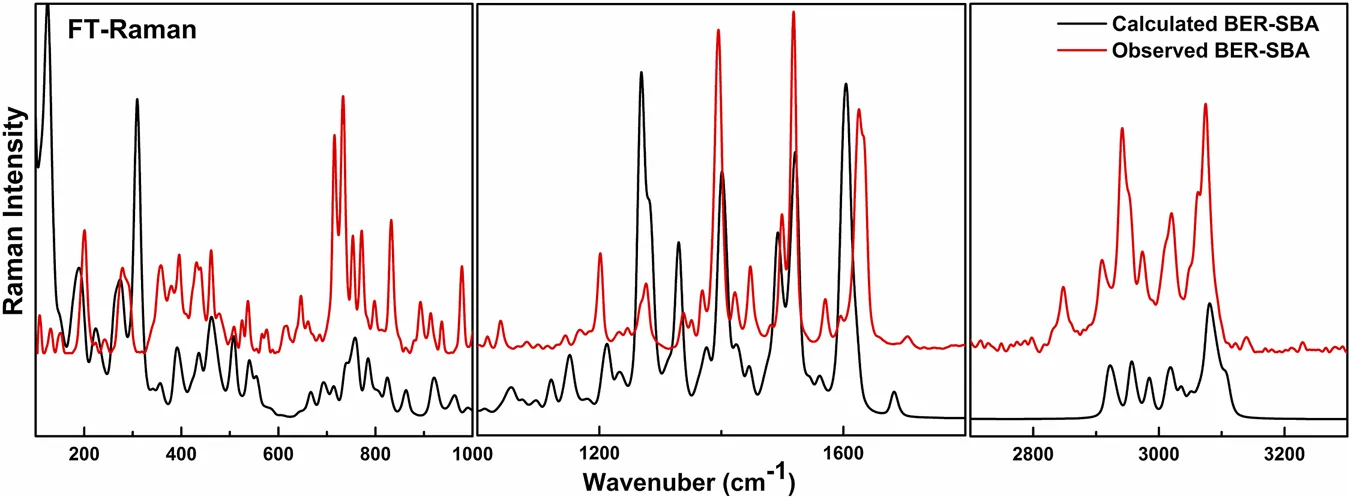
Experimental and calculated (scaled) Raman scattering spectra of the BER–SBA salt in the regions of 100–1,000, 1,000–1,900, and 2,000–3,300 cm^-1^.

**TABLE 1:** Observed and calculated bond length (Å) and stretching wavenumbers (cm^-1^) of modes of BerCl, SBA, and the BER–SBA salt involved in hydrogen bonding.

Molecule	C–H group		C=O group		O–H group	
	Bond length	Stretching wavenumber	Bond length	Stretching wavenumber	Bond length	Stretching wavenumber
	​	Raman	​	Raman	​	Raman
Experimental						
BerCl	0.9505 (C19–H20)	3,019	-	-	-	-
	0.9498 (C27–H28)	3,019	-	-	-	-
SBA	-	-	1.2339 (C13=O6)	1727	0.9863 (O2–H18)	-
	-	-	-	-	0.9813 (O5–H19)	-
BER–SBA salt	0.9002 (C70–H71)	3,071	1.1891 (C8=O2)	1704	0.8630 (O3–H4)	-
	0.9820 (C68–H69)	-	1.2809 (C26=O20)	1,625	0.8629 (O21–H22)	-
Theoretical						
BerCl	1.0841 (C19–H20)	3037	-	-	-	-
​	1.0841 (C27–H28)	3037	-	-	-	-
SBA	-	-	1.2049 (C13=O6)	1762	0.9694 (O2–H18)	3,569
	-	-	-	-	0.9694 (O5–H19)	3,561
BER–SBA salt	1.0804 (C68–H69)	3107	1.2357 (C8=O2)	1,684	1.0424 (O3–H4)	2,920
	1.0820 (C70-H71)	3088	1.2664 (C26=O20)	1,603	1.4460 (O21-H22)	2,904

#### 3.2.1 Ring vibrations

Typically stretching vibrational modes of the ring C–H group are found in the range of 3,100–3,000 cm^−1^ (Muthu et al., 2012). Stretching vibration of the C–H group (ring 1) in BerCl was appeared at 3,019 cm^−1^, with a medium-intensity Raman band corresponding to the calculated wavenumbers at 3,037 cm^−1^. Meanwhile, the same group was observed/calculated at 3,071/3,088 cm^−1^ in the BER–SBA salt, with a sharp peak in the Raman spectrum. Another C–H stretching vibration of rings 2 and 4 of BerCl was depicted at 3,078 cm^−1^, which has generated an experimental Raman band at 3,073 cm^−1^. The stretching vibrational frequency of the ring 2 C–H group was observed/calculated at 3,048/3,049 cm^−1^ in the BER–SBA salt, as shown in Figure 5. In case of SBA, the ring C–H stretching vibrations were found at 3,091, 3,079, 3,070, and 3,036 cm^−1^ in the Raman spectrum and calculated at 3,071, 3,063, 3,053, and 3,041 cm^−1^, respectively, whereas in the BER–SBA salt, the similar vibration attributed at 3,062 cm^−1^ in the Raman spectrum corresponding to the calculated wavenumber at 3,067 cm^−1^.

**FIGURE 5:**
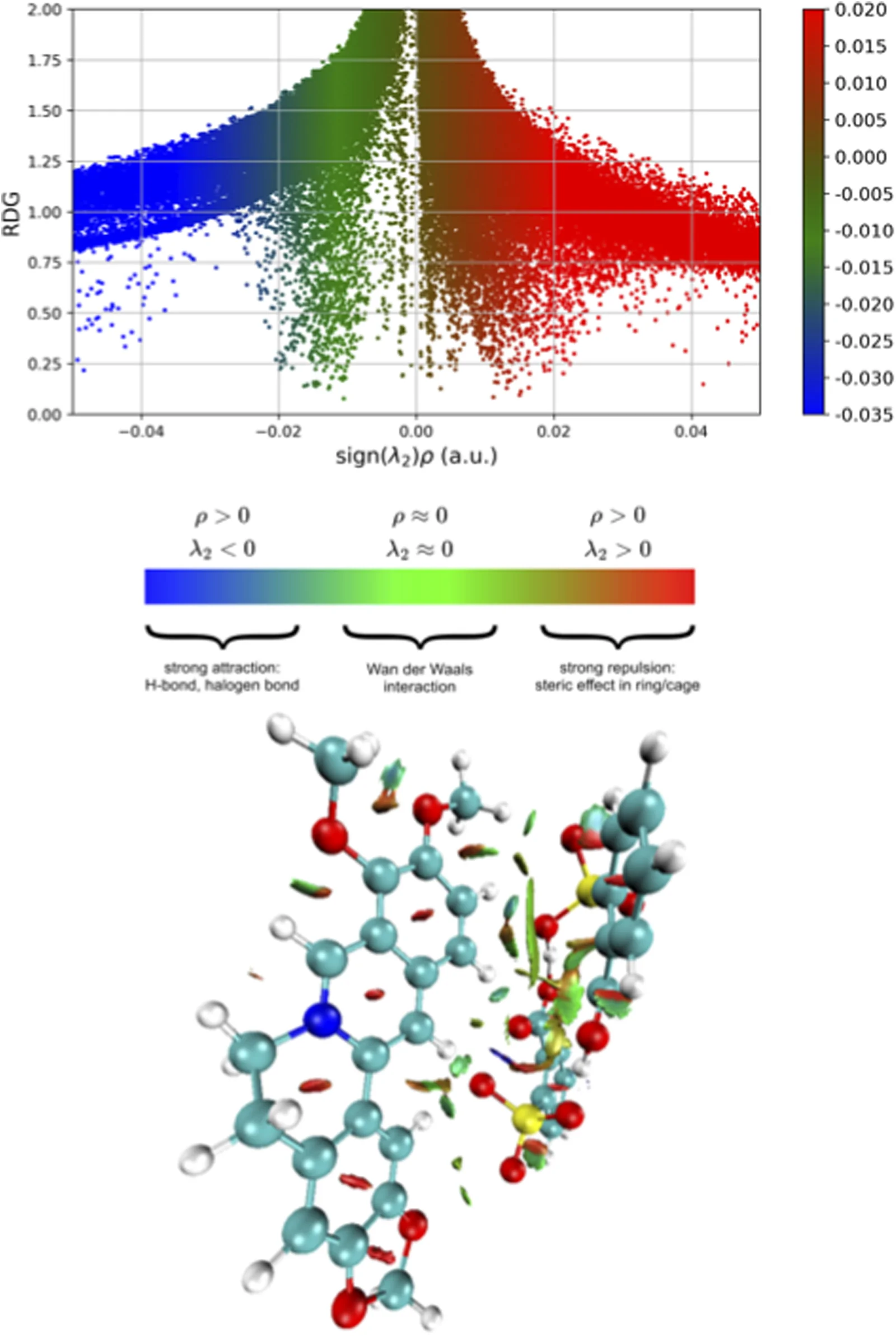
2D scatter and isosurface density plots illustrating the non-bonded interactions of the BER–SBA salt.

The in-plane bending vibration of the ring C–H group was observed at 1,236 cm^−1^ in the Raman spectrum, while the calculated value for BerCl was 1,252 cm^−1^. In the BER–SBA salt, the same group was generated/observed at 1,242/1,246 cm^−1^ in the Raman spectrum. Some vibrational modes corresponding to the in-plane bending of the ring C–H were observed/calculated at 1,445/1,446 cm^−1^ and 1,447/1,447 cm^−1^ in SBA/BER–SBA molecules, in the Raman spectrum. In addition, the vibrational modes of asymmetric deformation of the ring in BerCl/BER–SBA were observed at 731/714 cm^−1^ in the Raman spectrum corresponding to the calculated values at 731/712 cm^−1^. Similarly, the asymmetric deformation of the ring of SBA was calculated at 790 cm^−1^ and observed at 793 cm^−1^, but in the BER–SBA salt system, this was calculated/observed at 809/812 cm^−1^, in the Raman spectrum.

C–C stretching modes of BerCl and BER–SBA were experimentally recorded as a low-intensity Raman peak at 1,633 and 1,634 cm^−1^ attributed toward calculated bands at 1,631 and 1,619 cm^−1^, respectively. When a comparison was made among SBA and the BER–SBA salt, the same stretching vibrational mode was calculated/observed at 1,580/1,577 cm^−1^ and 1,575/1,570 cm^−1^, respectively, in the Raman spectrum. Another weak Raman band corresponding to C–C vibration was depicted at 1,301/1,320 cm^−1^; however, the same was calculated at 1,309/1,319 cm^−1^ in SBA/BER–SBA. It was noteworthy that the torsional ring vibrational mode was also observed/calculated in the coformer and BER–SBA salt system at 539/524 cm^−1^ and 535/536 cm^−1^, respectively.

#### 3.2.2 CH_2_ vibrational modes

Generally, methylene group stretching vibrational bands arise in the region 3,100–3,000 cm^−1^ (Lu et al., 2005). The asymmetric stretching mode of the CH_2_ group attached to ring 3 in BerCl was calculated at 2,964 cm^−1^, corresponding to a sharply intense Raman band at 2,955 cm^−1^. In the BER–SBA salt, the same vibrational mode has generated an intense Raman peak at 2,943 cm^−1^, which was calculated at 2,956 cm^−1^, whereas symmetric stretching vibration was observed/calculated at 2,909/2,906 cm^-1^ in BerCl and at 2,908/2,928 cm^−1^ in the BER–SBA salt. Apart from stretching vibrations, deformation of the CH_2_ group in API/salt was also obtained, where the theoretical wavenumbers were calculated at 1,514/1,520 cm^−1^ and intense Raman bands were observed at 1,518/1,517 cm^−1^. Wagging modes were further calculated at 1,391/1,393 cm^−1^, corresponding to the observed wavenumber at 1,397/1,395 cm^−1^ in the Raman spectrum, in both BerCl/BER–SBA molecules. Additionally, rocking vibrational modes of the CH_2_ group was observed at 912/936 cm^−1^ in the Raman spectrum and calculated at 922/935 cm^−1^ in both BerCl/BER–SBA.

#### 3.2.3 CH_3_ vibrational modes

Asymmetric stretching vibrational modes for CH_3_ groups were recorded at 3,009 and 3,010 cm^−1^ in the Raman spectrum, whereas they were calculated at 3,004 and 3,014 cm^−1^ in BerCl and the BER–SBA salt, respectively. On the other hand, the symmetric stretching vibrational mode was calculated at 2,885 cm^−1^ in BerCl and at 2,820 cm^−1^ in BER–SBA; however, this mode was recorded as an intense Raman peak at 2,849 cm^−1^ in both API and the salt system. Meanwhile, rocking vibrational modes were also obtained for CH_3_ groups, which were experimentally observed at 1,145 and 1,143 cm^−1^ in the Raman spectrum in BerCl and the BER–SBA salt, respectively, corresponding to theoretically obtained wavenumbers at 1,156 and 1,145 cm^−1^, respectively.

#### 3.2.4 (S=O) and (S–O) bonds vibrational modes

Stretching wavenumbers of the (S1=O) group were calculated at 1,142, 1,129, and 1,095 cm^−1^, corresponding to experimentally recorded values at 1,150, 1,135, and 1,079 cm^−1^, respectively, in the Raman spectrum in case of SBA, while the same group in the BER–SBA salt was calculated and observed at a wavenumber of 1,079 cm^−1^. Another stretching mode of (S1–O) of SBA was calculated and observed at 756 cm^−1^ in the Raman spectrum. Another type of bending vibrations viz. wagging, rocking, and twisting of (S1=O) was recorded/calculated at 570/573, 485/470, and 320/341 cm^−1^ in SBA, respectively, whereas asymmetric deformations of the (S=O) group were obtained at 394 and 378 cm^−1^ (in Raman), corresponding to the calculated wavenumbers at 397 and 390 cm^−1^, respectively, in the salt system.

#### 3.2.5 Groups involved in hydrogen bonding

Salt formation between BerCl (API) and SBA (coformer) occurred through intermolecular hydrogen bonding involving the C–H group of BerCl and the C=O and S=O groups of SBA. As a result, significant changes in bond lengths and vibrational wavenumbers were observed around these groups, as shown in Table 1. Two C–H groups of BerCl, namely, C19–H20 and C27–H28, were actively participated in salt formation with SBA. The stretching mode of vibration of (C19–H20) was recoded with a medium-intense peak at 3,019 cm^−1^ in Raman spectra in API, whereas the same mode was observed as at 3,071 cm^−1^ in BER–SBA. A similar kind of blue shifting was noticed in theoretical wavenumbers, which were calculated at 3,037 cm^−1^ (API) and at 3,088 cm^−1^ (salt). The bond length was also changed in C19–H20 (0.05Å/0.003Å) and C70–H71 (0.03Å/0.002Å) bonds in the API and salt system, respectively, in their respective observed/calculated values, which ensures that these groups were involved in intermolecular hydrogen bonding with SBA molecules. However, prominent shifting was not detected in the C–H modes as already discussed in Section 3.1.3 (Nishio, 2012).

Moreover, when a comparison was made among the coformer and salt, it was found that mainly the (C26=O20) and (S1–O7) groups of SBA were involved with (C70–H71) and (C68–H69) groups of BerCl, respectively, in the BER–SBA salt system (as discussed already) via C26=O20 … H71-C70 and S1-O7•••H69-C68 hydrogen bonds, as shown in Figure 3 and Table 1. C13=O6 in SBA, which was simulated at 1,762 cm^−1^, has generated a medium Raman band at 1,727 cm^−1^ and appeared with shifting toward lower wavenumber regions at 1,603 cm^−1^ (calculated) and 1,625 cm^-1^ (Raman spectrum), corresponding to the (C26=O20) group in the BER–SBA salt. Correspondingly, the observed/calculated bond lengths in the BER–SBA salt were also increased by 0.04/0.10 Å compared to those in SBA, confirming the red shifting. Another free C=O group of the salt system was calculated at 1,684 cm^-1^ and observed at 1,704 cm^-1^ in the Raman spectrum. It was noteworthy that the S1–O7 group was also affected due to its involvement with the C–H group of BerCl for the formation of the S1–O7•••H69–C68 bond, with decreases in wavenumber and elongation in the bond length in the salt system (Supplementary Table S3).

The BER–-SBA salt system also stabilized due to the existence of strong intermolecular hydrogen bonding between S–O and O–H groups of SBA molecules with the formation of S1–O7•••H22–O21 and S19–O25•••H4–O3 bonds (Figure 3). Theoretical bands corresponding to the hydroxyl group (O5–H19) were found at 3,561 cm^−1^ in SBA, whereas significant shifts (decrement) in the vibrational wavenumbers were calculated (O21–H22; 2,904 cm^−1^ and O3–H4; 2,920 cm^−1^) in the BER–SBA salt system, with an increase in the bond length, confirming the incorporation of these groups in strong intermolecular hydrogen bonding. It also confirmed that, among all, the S1–O7•••H22–O21 hydrogen bond is the strongest having the maximum shifting in the wavenumber (657 cm^-1^).

### 3.3 Topological and energy parameters at the bond critical path (BCP)

The QTAIM is a theoretical tool, which is used to provide the strength and nature of chemical bonding based on a definite criterion in the molecules (Bader, 1985). Koch and Popelier (1995) have developed a quantum chemical topology (QCT) to establish the changes in intra- and intermolecular hydrogen bonding via the electron density of compounds. It is a topological investigation of electron density (ρ_BCP_) with the Laplacian of electron density (∇^2^ρ_BCP_) at the bond critical point (BCP) and can be utilized in defining various other interactions such as non-covalent and van der Waals interactions (Lindquist-Kleissler et al., 2021). The value of ρ_BCP_ should lie in the range of (0.002–0.040 a. u.) and (0.024–0.139 a. u.) for ∇^2^ρ_BCP_ (Bader, 1985). According to the criterion by Rozas et al. (2000), hydrogen bonds can be convoluted as follows: (1) H_BCP_< 0 and ∇^2^ρ_BCP_ > 0 (partially covalent and medium in nature), (2) H_BCP_< 0 and ∇^2^ρ_BCP_ < 0 (covalent and strong), and (3) H_BCP_> 0 and ∇^2^ρ_BCP_ > 0 (weak and electrostatic in nature). The perception of conventional (strong) and non-conventional (weak) hydrogen bonds can also be defined using the QTAIM methodology by calculating the bond distances between the interacting atoms and the sum of their van der Waals radii (Desiraju, 2000).

In contrast, Hammami and Issaoui (2022) reported that the hydrogen binding energy can be calculated using the following equation:

$$E_{H\cdots O}=1/2\;V_{BCP.}$$

The illustration of the molecular graph for the intermolecular hydrogen bonding interactions in the BER–SBA salt system is provided in Figure 6, and their geometrical and topological parameters are given in Table 2. The geometrical parameters for the confirmation of the hydrogen bonding are described in Supplementary Table S7.

**FIGURE 6:**
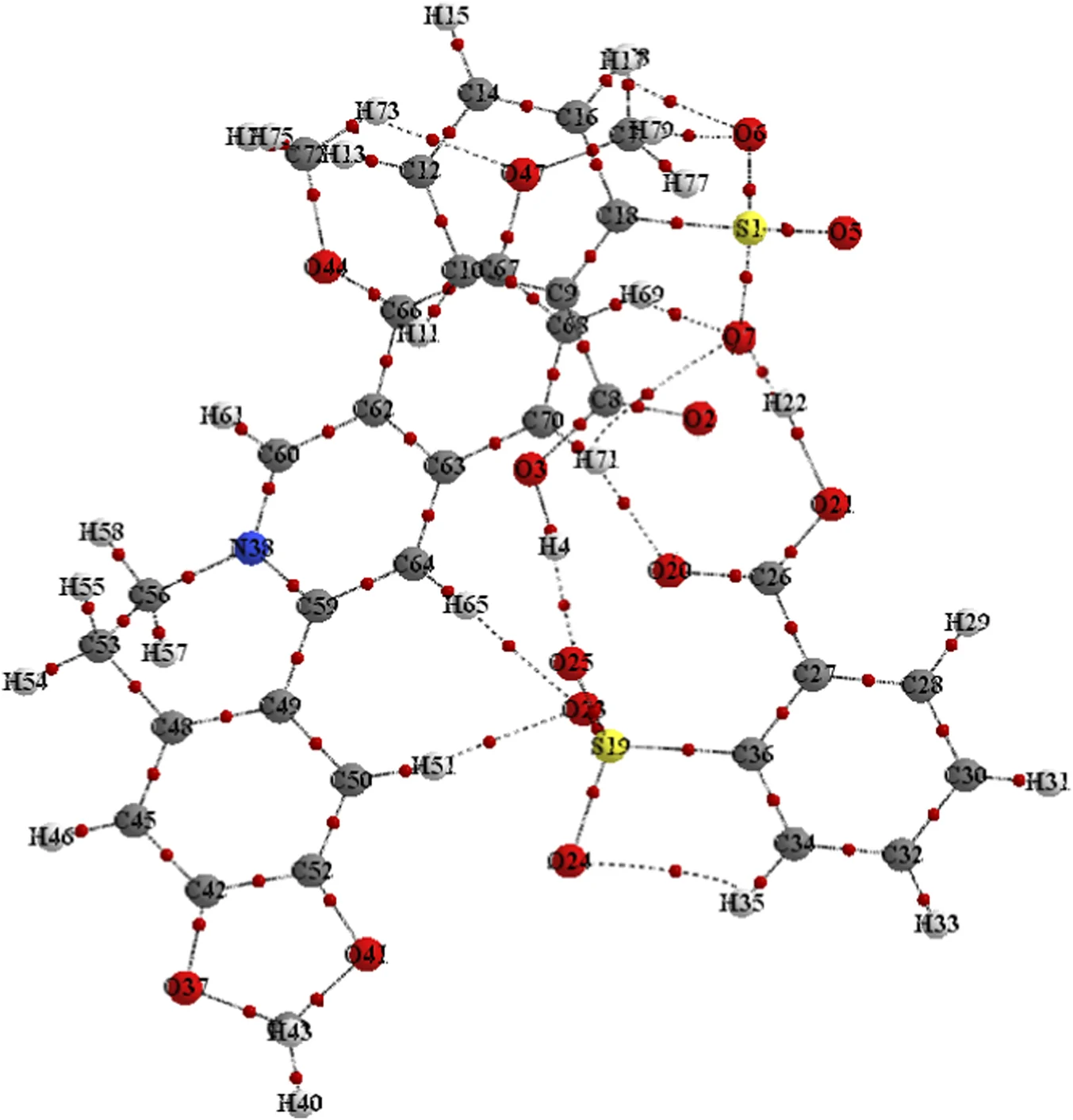
Molecular graph of the BER–SBA salt: bond critical points (small red spheres), ring critical points (small yellow sphere), and bond paths (pink lines).

**TABLE 2:** Geometrical parameter (bond length) and topological parameters for bonds of interacting atoms of intermolecular hydrogen bonding of the BER–SBA salt: electron density (ρ_BCP_), Laplacian of electron density (∇^2^ρ_BCP_), electron kinetic energy density (G_BCP_), electron potential energy density (V_BCP_), total electron energy density (H_BCP_) at bond critical point (BCP), and estimated interaction energy (E_int_).

Hydrogen bond	Bond length (Å)	ρ_BCP_ (a.u.)	∇^2^ρ_BCP_ (a.u.)	G_BCP_ (a.u.)	V_BCP_ (a.u.)	H_BCP_ (a.u.)	E_int_ (kcal mol^-1^)
S1–O7···H71	2.5117	0.0098	0.0386	−0.0015	−0.0067	−0.0082	−2.1103
S1–O6···H17	2.1957	0.0203	0.0765	−0.0023	−0.0145	−0.0168	−4.5407
O3–H4···O25	1.5347	0.0634	0.1613	0.0138	−0.0678	−0.0541	−21.2836
C26=O20···H71	2.0825	0.0188	0.0713	−0.0028	−0.0123	−0.0151	−3.8655
S1–O7···H22	1.0810	0.2426	−1.2806	0.3897	−0.4592	−0.0695	−144.0609
S19–O24···H35	2.3533	0.0151	0.0575	−0.0019	−0.0106	−0.0125	−3.3299
S19–O23···H51	2.1685	0.0164	0.0569	−0.0019	−0.0104	−0.0123	−3.2514
S19–O23···H65	2.1630	0.0161	0.0577	−0.0021	−0.0102	−0.0123	−3.1859
C67–O47···H73	2.1363	0.0197	0.0780	−0.0028	−0.0139	−0.0167	−4.3577
S1–O6···H79	2.2294	0.0148	0.0492	−0.0016	−0.0090	−0.0107	−2.8379
S1–O7···H69	2.0905	0.0210	0.0827	−0.0028	−0.0151	−0.0179	−4.7531

The results showed that in the BER–SBA salt, 11 oxygen–hydrogen (O•••H) BCPs (non-bonded interactions are represented by BCPs) are present, in which the strongest intermolecular hydrogen bond is (S1–O7•••H22) with maximum interaction energy (Eint) values of −144.0609; (∇^2^ρ_BCP_) <0 and H_BCP_< 0, which is strong, highly stable, ultra-short, low-barrier hydrogen bond that has crossed over into a shared covalent-like bonding network due to a near-complete proton-transfer event (Rejnhardt et al., 2025). Another one is (O3–H4•••O25) of medium and partially covalent nature as (∇^2^ρ_BCP_) > 0 and H_BCP_< 0 with Eint of −21.2836 kcal mol^-1^ (Nishio, 2012). It also proves that the BER–SBA salt is stabilized because of strong and medium intermolecular interactions (S1–O7•••H22) and (O3–H4•••O25), respectively, between the 2-sulfobenzoate dimer. According to QTAIM principles (Grabowski, 2001), this interaction (S1–O7•••H22) operates entirely within a shared-shell regime, rather than a closed-shell framework. This cooperative induction effect subsequently deactivates the thermodynamic driving force for a second proton transfer (H4) on the opposite side (Gilli et al., 1989). Consequently, the complex stabilizes into an asymmetric ground state, leaving the opposite pathway (O3–H4•••O25) as a conventional, partial covalent intermolecular hydrogen bond. Following intermolecular interactions (S1–O7•••H69) and (C26=O20•••H71) with Eint of −4.7531 and −3.8655 kcal mol^-1^, respectively, are also important as a linkage between Ber and SBA in the BER–SBA salt, which is of medium and partially covalent in nature (Table 2). Following Desiraju criteria (Desiraju, 2000), all the 10 hydrogen bonds among 11 are termed as medium-strength non-conventional bonds as H_BCP_< 0 and ∇^2^ρ_BCP_ > 0 having partially covalent character, except the one (S1–O7•••H69) with H_BCP_< 0 and ∇^2^ρ_BCP_ < 0 having strong, symmetric, or short hydrogen bond and covalent-like in nature (Rejnhardt et al., 2025) (Supplementary Table S7).

#### 3.3.1 Reduced gradient density (RDG) analysis

Non-covalent interactions are of great importance, but their accurate examination is still crucial. Reduced density gradient (RDG) analysis has been performed to study the non-covalent interactions (NCIs), such as hydrogen bonding, van der Waal, steric effect, in real space (Boto et al., 2016). Reduced density gradient is defined as follows (Johnson et al., 2010):

$$s\left(r\right)=\frac{1}{2{\left(3\pi^{2}\right)}^{1/3}}\frac{\left|\Delta \rho \left(r\right)\right|}{\rho {\left(r\right)}^{4/3}}.$$

s(r) = reduced density gradient.

*ρ*(r) = electron density.

Δ*ρ*(r) = gradient of electron density.

RDG analysis is widely used to investigate and visualize non-covalent interactions within and between molecules. It helps identify the nature of interactions, such as hydrogen bonding, van der Waals interactions, π–π interactions, and steric repulsion. NCI can be studied through electron density ρ(r), which implements the concepts of DFT, i.e., the electron density can be directly related to the energy, and therefore, it should be applicable to study interactions purely from the density (Laplaza et al., 2020). The inter and intra non-bonded interactions of the present molecular system have been explored through visual representation and the gradient isosurfaces and scatter graphs of the BER–SBA salt are shown in Figure 5. The scatter graph is plotted between RDG vs. sign (λ_2_)ρ, where sign (λ_2_)ρ is the second Eigen value of the electron density. For attractive interactions, (λ_2_)ρ < 0, whereas for repulsive interactions, (λ_2_)ρ > 0, and (λ2)ρ ∼ 0 predicts van der Waal interactions (Contreras Garcia et al., 2016; Saleh et al., 2012). (λ_2_)ρ lies between −0.035 and 0.020 a.u. in the RDG scatter graph, which is classified into three regions: red, blue, and green. In the RDG graph, the red regions highlighted within the range of 0.001–0.050 a.u. indicate steric repulsion predicted among all the rings of Ber and SBA molecules. This is the region of higher repulsive exchange contribution, whereas green color reflects vander Waal interactions, which were observed for C–H and CH_3_ groups of Ber and SO_3_, O–H, and C–H groups of SBA. On the other hand, a blue spike was observed between the O–H and C=O groups of SBA molecules, indicating strong attraction and the formation of an O7•••H22−O21 bond. The range of −0.02 to −0.05 a.u. confirms the presence of hydrogen bonding. Hence, RDG analysis demonstrated various interactions in the BER–SBA salt.

### 3.4 Natural bond orbital (NBO) analysis

Natural bond orbital (NBO) methods have emerged as a most promising tool for studying hyperconjugative interactions and electron transfer from filled lone-pair electrons, providing insights into molecular bonding, hybridization, and electron delocalization (Fatimaa et al., 2024; Pir et al., 2012). It describes covalent bonding through occupied natural orbitals and noncovalent interactions through donor–acceptor interactions between occupied and unoccupied orbitals. Thus, NBO analysis is particularly useful for evaluating intermolecular interactions, such as hydrogen bonding and associated charge transfer. The extent of charge transfer from bonding orbitals to anti-bonding orbitals is determined on the basis of stabilization energy, E(2). For each donor (*i*) and acceptor (*j*), the stabilization energy *E*(2) associated with the delocalization *i*→*j* is estimated using the following equation (Foster and Weinhold, 1980):

$$E\left(2\right)=\Delta E_{ij}=q_{i}\frac{{F\left(i,j\right)}^{2}}{\varepsilon_{i}-\varepsilon_{j}},$$

where *q*_i_ is the donor orbital occupancy, *ε*_*i*_ and *ε*_j_ are diagonal elements, and *F*(*i*, *j*) is the off-diagonal NBO Fock matrix element.

E(2) denotes the strength of the bonds, as well as the stability of the molecular system to be examined. A greater E(2) value reflects a stronger interaction between the donor and acceptor moieties (Srivastava et al., 2025; Nagy, 2014). The selected inter- and intramolecular hydrogen bonding interactions between filled donor and empty acceptor orbitals with values of E(2) of the BER–SBA salt are tabulated in Supplementary Table S8.

The presence of hyperconjugative interactions among BerCl and SBA highlighted the presence of inter- and intramolecular hydrogen bonding, which are useful in the determination of stability of the studied system. The most intensive charge transfer were observed as LP (2) O3→π*(O2−C8) within unit 1, i.e., SBA with a maximum stabilization energy of 27.31 mol^-1^. Another intramolecular charge transfer within SBA was noticed with a stabilization energy of 36.64 mol^-1^, taking place between lone pair of O20 and π*(Ο21−C26). These interactions were responsible for the stability of SBA. Furthermore, within unit 3 (BerCl), the strong charge transfer occurred between LP (2) O47→π*(C66−C67) and LP (2)→π*(C42−C45) with interaction energies of 14.67 and 13.80 mol^-1^, respectively, provided the stability to BerCl. Despite the occurrence of intramolecular charge transfer, intermolecular interactions were also found, leading to salt formation.

Intermolecular charge transfer existed among two molecules of SBA, namely, LP (3) O7→ LP*(1) Η22 and LP (3) O25→LP*(1) H4, reflected the presence of a strong intermolecular hydrogen bond between them with highest stabilization energies 148.01 and 23.07 kcal mol^-1^. The occurrence of strong intermolecular hydrogen bonds viz. O7•••H22−O21 and O25•••H4−O3 is responsible for the stability of SBA. On the other side, various intermolecular charge transfers were observed between BerCl and SBA, with stabilization energies of 2.02 mol^-1^ (LP (1) O7→σ*(C68−H69)) from unit 1 to 3 and 2.36 mol^-1^ (LP (2) O20→σ*(C70−H71)) from unit 2 to 3, as shown in Supplementary Table S8. These interactions confirm the existence of C68−H69•••O7 and C26−O20•••H71 hydrogen bonds, which is responsible for salt formation. It may be concluded that most intensive interactions arise due to the transfer of electron density from lone pair bonding orbitals to π* and σ* antibonding orbitals. Hence, NBO studies supported the presence of hydrogen bond interaction between BerCl and SBA, leading to salt formation.

### 3.5 Molecular electrostatic potential (MEP) topology

The molecular electrostatic potential (MEP) serves as an analytical tool of electron density in a molecular system. It is very useful in predicting the chemical properties such as reactivity, electrophilic sites, and nucleophilic sites in a given molecule (Politzer and Murray, 2002; Akman et al., 2020; Jamal et al., 2024). Moreover, it facilitates the phenomenon of biological recognition and interactions, which include hydrogen bonding (Drissi et al., 2015). The electrostatic potential areas are identified by different colors at the MEP surface. Blue represents positive (electron-deficient), red shows negative (electron-rich), and green denotes the neutral regions (Gokce et al., 2022). The MEP at a given point r (x, y, z) in the vicinity of a molecule can be defined as the force acting on a positive test charge (a proton) located at ‘r’. The electrical charge cloud generated through the molecules, electrons, and nuclei are given using the following expression (Politzer and Murray, 2002):

$$\left(r\right)=\sum_{A}\frac{Z_{A}}{R_{A}-r}-\int \frac{\rho \left(r^{\prime }\right)dr^{\prime }}{\left|r^{\prime }\left.-r\right|\right.},$$

where Z_A_ is the charge on nucleus A located at R_A_ and *ρ*(r^’^) is the electronic density. The first term in the expression represents the effect of the nuclei, and the second represents that of electrons.

Supplementary Figures S7, S8 and Figure 7 depict the MEP surfaces of Ber, SBA, and the BER–SBA salt system, respectively. Negative regions (red and yellow) are observed in Cl and C=O groups, indicating possible sites for electrophilic attack, whereas the positive section (blue) are located on the hydrogen atoms of CH_2_, CH_3_, and OH groups, representing probable sites for nucleophilic attack in Ber and SBA, respectively (Supplementary Figures S7 and S8).

**FIGURE 7:**
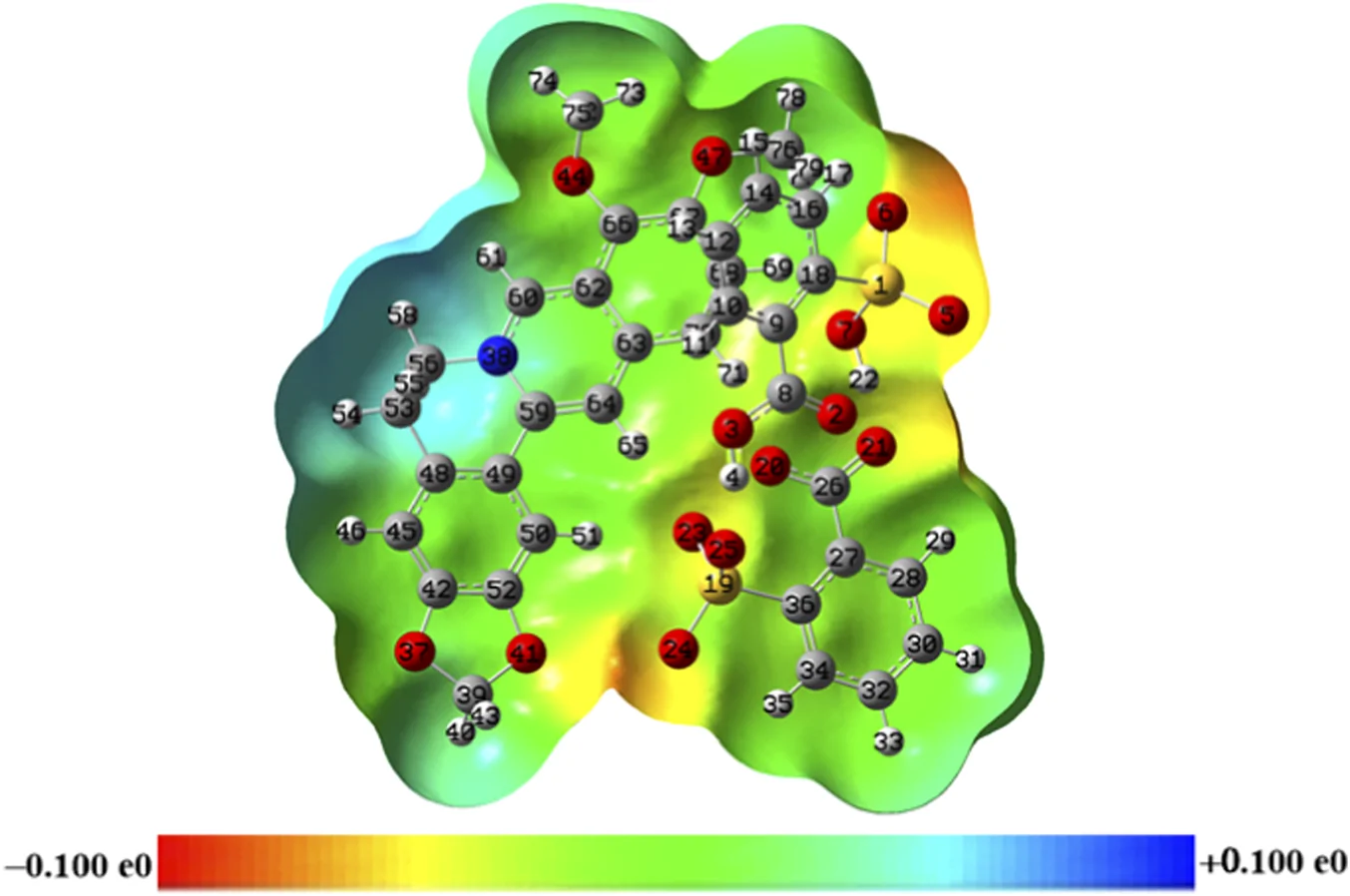
Molecular electrostatic potential (MEP) map formed by mapping of total electron density over electrostatic potential in the gas phase for the BER–SBA salt.

In the BER–SBA salt, the electronegative portion is found near oxygen atoms of SO_3_ and C=O groups, whereas the hydrogen of rings containing nitrogen atom demonstrate the electropositive region due to its electropositive character (Figure 7). These groups also participate in intermolecular hydrogen bonding with the neighboring molecule in crystal structure, as shown in Figure 1. The remaining nucleophilicities of (S1–O7 and C26=O20) and electrophilicities of (C68–H69 and C70–H71) groups of Ber and SBA, respectively, were neutralized in the BER–SBA salt, which proves the formation of intermolecular hydrogen bonding (S1–O7•••H69–C68; C26=O20•••H71–C70), as depicted in Figure 3. The same phenomenon occurs between the interacting groups of two SBA molecules (Figures 3, 7).

### 3.6 Chemical reactivity analysis

The chemical reactivity of the molecule can be monitored by exploring frontier molecular orbitals and various reactivity descriptors as they play an important role in quantum chemistry (Wolff et al., 2012; Pal and Chattaraj, 2021).

#### 3.6.1 Frontiers molecular orbitals (FMOs)

The frontier molecular orbitals (FMOs) are used to understand the reactivity and selectivity of the molecule, thereby helping recognize the motifs behind the mechanisms of their chemical reactions (Karaman, 2015; Liu and Schauer, 2015). FMOs are known as the highest occupied molecular orbitals (HOMOs) and the lowest unoccupied molecular orbitals (LUMOs), which represent the capacity to donate and accept an electron, respectively (Sahin and Dege, 2021). The minimum energy required to most common and essential mechanism of energy transfer in a system is to push an electron with a minimum amount of energy, which is represented by the energy gap. The energy gap (ΔE) between HOMO and LUMO (E_L_ − E_H_) is the minimum energy required to push an electron, which plays an essential role in verifying several phenomena such as chemical reactivity and stability of the molecule (Megrouss et al., 2019). The lower value of ΔE indicates high chemical reactivity and low kinetic stability (Shamina et al., 2024). The HOMO–LUMO plots and ΔE values of BerCl, SBA, and the BER–SBA salt are represented in Supplementary Figures S9, S10, and Figure 8, respectively and Table 3. The ΔE value of the salt system is less (2.3054 eV) than that of BerCl (3.7432 eV) and SBA (5.6382 eV). Therefore, the BER–SBA salt system is more reactive, soft, less stable, and more polarizable than BerCl (API). Figure 8 shows that charge transfer occurred between the Ber moieties because the HOMO and LUMO orbitals are mainly localized on these moieties.

**FIGURE 8:**
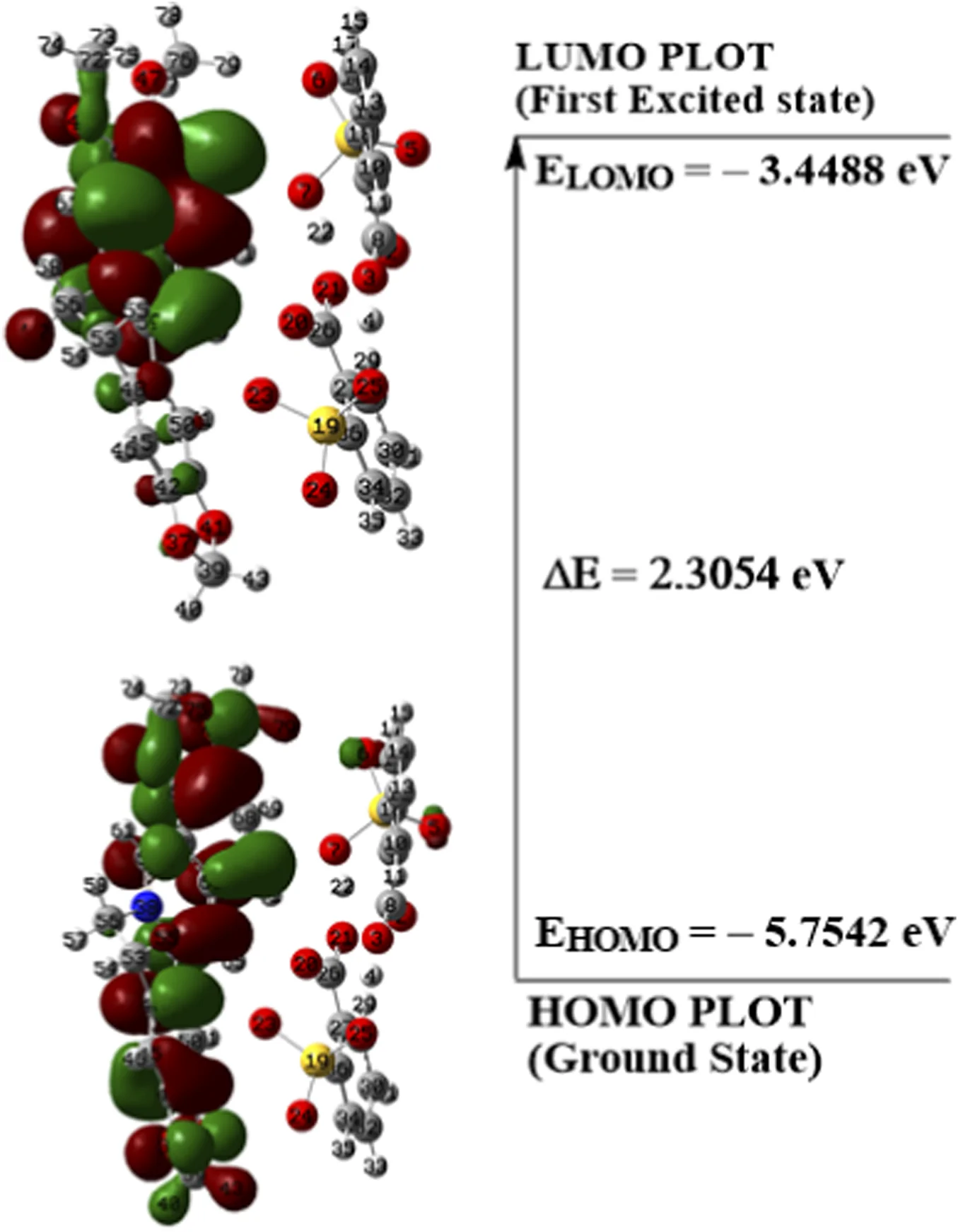
HOMO–LUMO plot with their energy gap (ΔE) of the BER–SBA salt with orbital involved in electronic transitions.

**TABLE 3:** The calculated EHOMO, ELUMO, energy band gap (EL-EH), chemical potential (μ), electro negativity (χ), global hardness (η), global softness (S) and global electrophilicity index (ω) at 298.15 K for BerCl, SBA and BER-SBA salt using B3LYP/6-311++G(d,p).

Molecules	EH (eV)	EL (eV)	EL-EH (eV)	χ (eV)	μ (eV)	η (eV)	S (eV)	ω (eV)	ΔNmax
BerCl (API)	-5.2897	-1.5464	3.7432	3.417	-3.417	1.872	0.267	3.1194	1.8257
SBA (coformer)	-7.954	-2.3157	5.6382	5.135	-5.135	2.819	0.177	4.6764	1.8214
BER-SBA salt	-5.7542	-3.4488	2.3054	4.602	-4.602	1.153	0.434	9.1844	3.9919

#### 3.6.2 Global reactivity descriptors

Global reactivity descriptors are used in determining the chemical reactivity, stability, and charge transfer of the investigated system (Parr et al., 1999; Lesar and Milošev, 2009). The HOMO and LUMO energy levels (E_H_ and E_L_, respectively) play an important role in calculating the global reactivity descriptors, such as electro negativity (χ), chemical potential (μ), global hardness (η), global electrophilicity index (ω), and global softness (S) (Rijal et al., 2023; Vijayaraj et al., 2009). These parameters for Ber, SBA, and the BER–SBA salt systems are illustrated in Table 3.

These parameters were derived on the basis of Koopman’s theory and calculated using energies of HOMO and LUMO as follows (Parr et al., 1999):

$$\chi =-\frac{1}{2}\left(E_{HOmo}+E_{LUMO}\right),$$

$$\mu =-\chi =-\frac{1}{2}\left(E_{HOmo}+E_{LUMO}\right),$$

$$\eta =\frac{1}{2}\left(E_{LUMO}+E_{HOMO}\right),$$

$$\omega =\frac{\mu^{2}}{2\eta },$$

$$S=\frac{1}{2\eta }.$$

The values of η and ΔE are inversely proportional to intramolecular charge transfer, and therefore, there is low charge transfer or less exchange of electrons if it is large (Sert et al., 2018). The salt system has smaller values of η and ΔE than the API and conformer, demonstrating that there is considerable charge transfer in the salt system and it is biologically active. The value of ω signifies the extent of the energy reduction produced by the charge transfer between the donor and the acceptor (Abdulridha et al., 2020). Additionally, the high value of ω and S for the salt system indicates its electrophilic character and soft nature, respectively (Pearson, 2005).

Electrophilicity charge transfer (ECT) is particularly determined using the ΔN_max_ values of the interacting molecules, i.e., A and B, and is calculated as [(ΔN_max_)A − (ΔN_max_)B]. ECT evaluation suggests that if (ECT <0), then the charge flows from A to B, and if (ECT >0), the charge flows from B to A. In our present study, the two interacting moieties are SBA (A) and BerCl (B), and from Table 3, it is clearly observed that ECT <0 (−0.0043), which indicates that charge flows from SBA (coformer) to BerCl (API). Therefore, SBA behaves as an electron donor, and BerCl acts like an electron acceptor.

### 3.7 Molar refractivity (drug-likeness)

The structural property of ligands, i.e., drug-likeness, is a crucial parameter used to adopt efficient and complete pathways in the discovery and development of new oral dosage forms (Mir, 2023). The molecular size and polarizability of molecular systems were evaluated based on molar refractivity, which are significantly used for predicting how a molecule interacts with biological targets and membranes. Molar refractivity (MR) represents the real volume and is also related to the London-dispersive forces that plays significant role in drug–receptor interactions (Ali et al., 2006). The drug-likeness of BerCl and the BER–SBA salt was estimated using the Lorentz–Lorenz equation (Ali et al., 2006). The molar refractivity values of BerCl and the BER–SBA salt were calculated to be 54.51 and 104.43 e.s.u, respectively, confirming that both modifications fall within the acceptable drug-like chemical space (Ali et al., 2006).

### 3.8 Molecular docking studies

Molecular docking simulations were employed as an efficient *in silico* approach to evaluate the interactions between a target protein and a ligand (molecule or drug) (Sadeghian et al., 2025). The AutoDock Tool (version 1.5.6) was used for docking simulations to provide insights into ligand–receptor interactions (Millan-Casarrubias et al., 2025). Grid maps were generated with a grid spacing of 0.5 Å to facilitate ligand binding. The active site of each macromolecule was defined within a grid box of 60 Å × 60 Å × 60 Å prior to docking; the protein structures were prepared by removing water molecules, co-crystallized ligands, and cofactors. The Lamarckian Genetic Algorithm (LGA), implemented in AutoDock (Millan-Casarrubias et al., 2025), was used for the docking calculations.

The present study aimed to investigate the biological activities of Ber using various protein targets. Ber was evaluated individually against the selected biological targets because conventional tools are designed for discrete protein–ligand systems, and non-covalently bound complexes may dissociate under biological conditions (Roy et al., 2023). Three protein targets were chosen: 3-hydroxy-3-methylglutaryl-coenzyme A (HMG-CoA; PDB code: 1T02), mitogen-activated protein kinase (MAPK; PDB code: 1HZM), and Nuclear factor erythroid 2-related factor (Nrf2; PDB code: 7K2F) to investigate the potential lipid- and cholesterol-lowering, anti-inflammatory, and antioxidant activities (Sun et al., 2024) of the studied drug molecule, respectively. Out of all the docked conformations, one which bound well at the active site was examined for detailed interactions using Discovery Studio Visualizer 4.5 software (BIOVIA, 2020). Ligand–protein interactions and their binding affinities are illustrated in Figure 9. The figure identifies the specific amino acids that interact with Ber and classifies different types of molecular interactions. The ligand formed stable complexes with HMG-CoA through three conventional hydrogen bonds, as shown in Supplementary Table S9. The interacting residues were ALA B: 331 (3.31 Å), GLY B: 330 (3.13 Å), and ASP B: 283 (3.05 Å), with a binding energy of −5.79 kcal/mol. Ber also interacted with MAPK and Nrf2 through conventional hydrogen bonding. For MAPK, the key interacting residues were CYS A: 147 (1.94 Å), GLU A: 148 (2.16 Å), and ARG B: 470 (2.63 Å), while for Nrf2, the major interacting residues were ARG B: 470 (2.63 Å) and HIS B: 516 (2.66 Å). The corresponding binding energies were −7.14 and −9.28 kcal/mol, respectively. Overall, the docking results indicate favorable interactions of Ber with the selected protein targets and provide *in silico* support for its reported biological activities (Doggrell, 2005; Kupeli et al., 2002; Yesilada et al., 2002; Misik et al., 1995; Sun et al., 2024).

**FIGURE 9:**
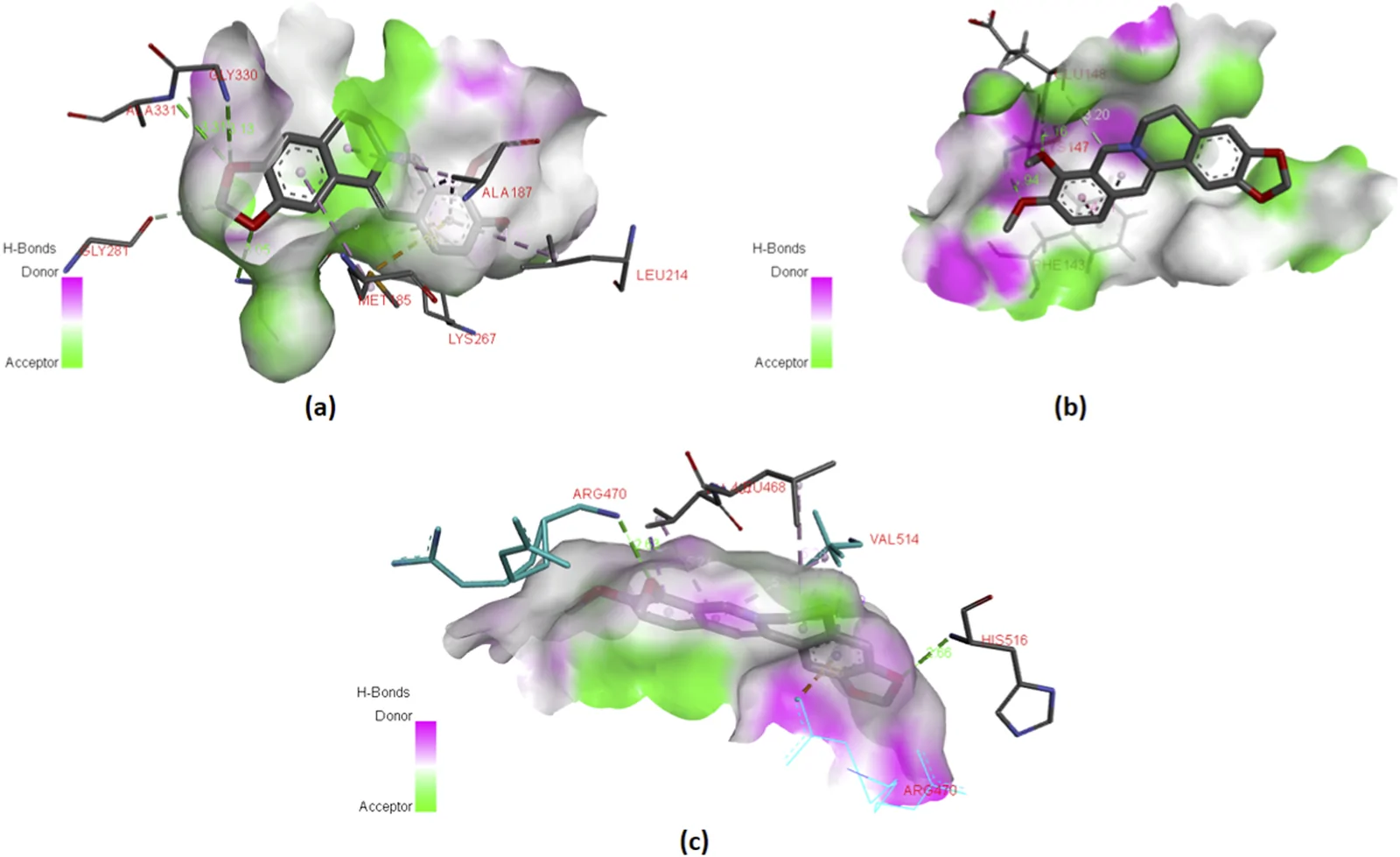
Ligand-binding interactions of berberine with **(a)** 3-hydroxy-3-methylglutaryl-coenzyme A (HMG-CoA), **(b)** mitogen-activated protein kinase (MAPK), and **(c)** nuclear factor erythroid 2-related factor (Nrf2).

## 4 Conclusion

A new crystalline BER–SBA salt was successfully obtained by hydrothermal crystallization and structurally established by single-crystal X-ray diffraction as a 1:1 ionic solid. The crystal structure comprises a well-defined supramolecular architecture sustained by C–H···O interactions between BER and SBA, together with prominent O–H···O interactions involving neighboring SBA units. The close agreement between the experimental bulk PXRD pattern and that simulated from the single-crystal structure confirms the formation of a distinct and phase-pure crystalline form. FT–Raman spectroscopy further supports salt formation through characteristic vibrational changes associated with the intermolecular interaction network.

BER–SBA also exhibits a markedly different thermal profile from the BerCl sample investigated. The salt shows a substantially higher DSC onset temperature (257.6 °C ± 0.5 °C) and peak temperature (264.2 °C ± 0.5 °C), together with the absence of an initial mass-loss event and a single-step decomposition following melting. These observations indicate greater apparent thermal robustness of BER–SBA up to its melting transition. From a pharmaceutical solid-form perspective, the formation of a phase-pure crystalline salt with a well-defined lattice and higher thermal transition is particularly relevant because such characteristics may provide advantages during solid-form selection, processing, storage, and formulation development, subject to confirmation by further pharmaceutical performance studies.

The computational analyses performed using a 1:2 BER–SBA cluster selected to represent key local interactions within the experimentally established 1:1 crystal lattice, providing a molecular-level interpretation of the observed solid-state behavior. The calculated vibrational shifts, QTAIM and RDG/NCI analyses, NBO donor–acceptor interactions, and MEP redistribution consistently support a cooperative interaction network dominated by O–H···O interactions between SBA units and supplemented by C–H···O interactions between BER and SBA. The smaller calculated HOMO–LUMO gap and associated global reactivity descriptors further demonstrate that salt formation modifies the electronic environment, softness, and charge-transfer characteristics of the molecular assembly. These descriptors provide mechanistic insight into the electronic consequences of salt formation. Furthermore, molecular docking results provide information regarding the potential interactions of Ber with the selected biological targets.

Overall, BER–SBA represents a promising new solid form of Ber with a distinct crystal structure and enhanced apparent thermal robustness relative to the investigated BerCl material. The combined experimental and computational findings demonstrate how salt formation can modify both the crystal packing and solid-state properties of ber and provide a rational basis for further pharmaceutical evaluation.

## Data Availability

The original contributions presented in the study are included in the article/Supplementary Material; further inquiries can be directed to the corresponding authors.

## References

[B1] Abdulridha A. A. Mahmood A. Albo H. A. Sajjad Q. M. Sert Y. Salman H. E. (2020). Corrosion inhibition of carbon steel in 1M H2SO4 using new azo schiff compound: electrochemical, gravimetric, adsorption, surface and DFT studies. J. Mol. Liq. 315, 113690. 10.1016/j.molliq.2020.113690

[B2] Ahmed H. Shimpi M. R. Velaga S. P. (2017). Relationship between mechanical properties and crystal structure in cocrystals and salt of paracetamol. Drug Dev. Ind. Pharm. 43 (1), 89–97. 10.1080/03639045.2016.1220568 27486671

[B3] Akman M. Ata A. C. Yildiko Ü. Cakmak İ. (2020). Molecular structure, frontier molecular orbitals, NBO, MESP and thermodynamic properties of 5,12-dibromo perylene with DFT calculation methods. Int. J. Chem. Technol. 4 (1), 49–59. 10.32571/ijct.695754

[B4] Ali A. S. Sabir C. D. Nain A. K. (2006). Volumetric, viscometric, and refractive index behaviour of α-amino acids and their groups’ contribution in aqueous D-glucose. J. Chem. Thermodyn. 38 (2), 136–143. 10.1016/j.jct.2005.04.011

[B5] Bader R. F. (1985). Atoms in molecule. Acc. Chem. Res. 18 (1), 9–15. 10.1021/ar00109a003

[B6] Battu S. K. Repka M. A. Maddineni S. Chittiboyina A. G. Avery M. A. Majumdar S. (2010). Physicochemical characterization of berberine chloride: a perspective in the development of a solution dosage form for oral delivery. AAPS PharmSciTech 11 (3), 1466–1475. 10.1208/s12249-010-9520-y 20842541 PMC2974104

[B7] Becke A. D. (1993). Density-functional thermochemistry. III. The role of exact exchange. J. Chem. Phys. 98, 5648–5652. 10.1063/1.464913

[B8] BIOVIA (2020). BIOVIA Discovery Studio Visualizer. San Diego: Dassault Systèmes. Available online at: https://www.3ds.com/products/biovia/discovery-studio/visualization (Accessed September 23, 2026).

[B9] Bloino J. Biczysko M. Barone V. (2015). Anharmonic effects on vibrational spectra intensities: Infrared, raman, vibrational circular dichroism, and raman optical activity. J. Phys. Chem. A 119 (49), 11862–11874. 10.1021/acs.jpca.5b10067 26580121 PMC5612400

[B10] Boto R. A. Contreras-Garcíz J. Tierny J. Piquemal J. P. (2016). Interpretation of the reduced density gradient. Mol. Phys. 114 (7-8), 1406–1414. 10.1080/00268976.2015.1123777

[B11] Boys S. F. Bernardi F. (1970). The calculation of small molecular interactions by the differences of separate total energies. Some procedures with reduced errors. Mol. Phys. 19 (4), 553–566. 10.1080/00268977000101561

[B12] Chahine J. Saffon N. Cantuel M. Fery-Forgues S. (2025). Spontaneous formation of fluorescent nanofibers and reticulated solid from berberine palmitate: a new example of aggregation-induced emission enhancement in organic ion pairs. Langmuir 27 (6), 2844–2853. 10.1021/la104302d 21338124

[B13] Contreras Garcia J. Boto R. A. Izquierdo Ruiz V. Reva I. Woller T. Alonso M. A. (2016). A benchmark for the non-covalent interaction (NCI) index or is it really all in the geometry? Theor. Chem. Acc. 135 (1), 1–14. 10.1007/s00214-016-1977-7

[B14] Crystal-Impact (2008). Brandenburg & Putz. Bonn.

[B15] Dennington R. Keith T. Millam J. (2016). Gaussview, Version 6. Shawnee Mission, K S: Semichem Inc.

[B16] Desiraju G. R. (2000). Hydrogen bonds and other intermolecular interactions in organometallic crystals. J. Chem. Soc. Dalton Trans. 21, 3745–3751. 10.1039/b003285i

[B17] Doggrell S. A. (2005). Berberine−a novel approach to cholesterol lowering. Expert Opin. Investig. Drugs 14 (5), 683–685. 10.1517/13543784.14.5.683 15926873

[B18] Dostál J. Man S. Seckárová P. Hulová D. Necas M. Potácek M. (2004). Berberine and coptisine free bases. J. Mol. Struct. 687 (1-3), 135–142. 10.1016/j.molstruc.2003.09.018

[B19] Drissi M. Benhalima N. Megrouss Y. Rahmani R. Chouaih A. Hamzaoui F. (2015). Theoretical and experimental electrostatic potential around the m-Nitrophenol. Molecules 20 (3), 4042–4054. 10.3390/molecules20034042 25741898 PMC6272371

[B20] Fatimaa M. Siddiqui W. A. Choudhary M. I. Ashraf A. Niaz S. Raza M. A. (2024). Synthesis of dimeric 1, 2-benzothiazine 1, 1-dioxide scaffolds: molecular structures, hirshfeld surface analysis, DFT and enzyme inhibition studies. RSC Adv. 14 (24), 16935–16944. 10.1039/d4ra02009j PMC11130764 38808235

[B21] Foster J. P. Weinhold F. (1980). Natural hybrid orbitals. J. Am. Chem. Soc. 102 (24), 7211–7218. 10.1021/ja00544a007

[B22] Frisch M. J. Choudhary M. I. Ashraf A. (2016). Gaussian 16, Revision C.01. Wallingford CT: Gaussian Inc.

[B23] Gilli G. Bellucci F. Ferretti V. Bertolasi V. (1989). Evidence for resonance -assisted hydrogen bonding from crystal-structure correlations on the enol form of the.beta.-diketone fragment. J. Am. Chem. Soc. 111 (3), 1023–1028. 10.1021/ja00185a035

[B24] Gokce H. Sen F. Sert Y. Abdel-Wahab B. F. Kariuki B. M. El-Hiti G. A. (2022). Quantum computational investigation of (E)-1-(4-methoxyphenyl)-5-methyl-N0-(3- phenoxybenzylidene)- 1H-1,2,3-triazole-4-carbohydrazide. Molecules 27 (7), 2193. 10.3390/molecules27072193 PMC9000758 35408592

[B25] Grabowski S. J. (2001). A new measure of hydrogen bonding strength – *ab initio* and atoms in molecules studies. Chem. Phy Lett. 338 (4-6), 361–366. 10.1016/s0009-2614(01)00265-2

[B26] Hammami F. Issaoui N. (2022). A DFT study of the hydrogen bonded structures of pyruvic acid-water complexes. Front. Phys. 10, 901736. 10.3389/fphy.2022.901736

[B27] Hasa D. Marosa M. Bucar D. Corpinot M. K. Amin D. Patel B. (2020). Mechanochemical formation and disappearance of caffeine-citric-acid cocrystal polymorphs. Cryst. Growth Des. 20 (2), 1119–1129. 10.1021/acs.cgd.9b01431

[B28] Hohenberg P. Kohn W. (1964). Inhomogeneous electron gas. Phys. Rev. B 136, 864–871. 10.1103/physrev.136.b864

[B29] Hubschle C. B. Sheldrick G. M. Dittrich B. (2011). ShelXle: a Qt graphical user interface for SHELXL. J. Appl. Crystallogr. 44 (6), 1281–1284. 10.1107/S0021889811043202 22477785 PMC3246833

[B30] Jamal A. Haque Faizi M. S. Dege N. (2024). Synthesis, structural characterization, DFT calculations, and molecular docking of a novel quinoline derivative. J. Mol. Struct. 1300, 137251. 10.1016/j.molstruc.2023.137251

[B31] Jantova S. Cipak L. Cernakova M. Kost'alova D. (2003). Effect of berberine on proliferation, cell cycle and apoptosis in HeLa andL1210 cells. J. Pharm. Pharmacol. 55 (8), 1143–1149. 10.1211/002235703322277186 12956905

[B32] Johnson E. R. Keinan S. Mori-Sa´ nchez P. Contreras-Garcı´a J. Cohen A. J. Yang W. (2010). Revealing noncovalent interactions. J. Am. Chem. Soc. 132, 6498–6506. 10.1021/ja100936w PMC2864795 20394428

[B33] Karaman R. (2015). From conventional prodrugs to prodrugs designed by molecular orbital methods. Front. Comput. Chem. 1, 187–249. 10.2174/9781608059782115020007

[B34] Kariuki B. M. Jones W. (1995). Five salts of berberine. Acta Cryst. C51, 1234–1240. 10.1107/s010827019401259x

[B35] Kavanagh O. N. Croker D. M. Walker G. M. Zaworotko M. J. (2019). Pharmaceutical cocrystals: from serendipity to design to application. Drug Discov. Today 24 (3), 796–804. 10.1016/j.drudis.2018.11.023 30521935

[B36] Keith T. A. (2009). AIMALL, Version 090201. Overland Park, KS: TK Gristmill Software.

[B37] Kettmann V. Kosfalova D. Jantova S. Cernakova M. Drimal J. (2004). *In-vitro* cytotoxicity of berberine against HeLa and L1210 cancer cell lines. Pharmazie 59 (7), 548–551. 15296093

[B38] Khanum G. Ali A. Shabbir S. Fatima A. Alsaiari N. Fatima Y. (2022). Vibrational spectroscopy, quantum computational and molecular docking studies on 2-[(1h-benzimidazol-1-yl)-methyl]benzoic acid. Crystals 12 (3), 337. 10.3390/cryst12030337

[B39] Ko W. H. Yao X. Q. Lau C. W. Law W. I. Chen Z. Y. Wok W. K. (2009). Vasorelaxant and antiproliferative effects of berberine. Eur. J. Pharmacol. 399 (2-3), 187–196. 10.1016/s0014-2999(00)00339-3 10884519

[B40] Koch U. Popelier P. L. (1995). Characterization of CHO hydrogen bonds based on the charge density. J. Phys. Chem. 99 (24), 9747–9754. 10.1021/j100024a016

[B41] Kornilova T. Glebov V. Castañeda R. Timofeeva T. V. (2022). A new pseudopolymorph of berberine chloride: crystal structure and hirshfeld surface analysis. Acta Crystallogr. E Crystallogr. Commun. 78 (5), 468–472. 10.1107/s2056989022003309 PMC9069517 35547800

[B42] Kupeli E. Kosar M. Yesilada E. Husnu K. Baser C. A. (2002). Comparative study on the anti-inflammatory, antinociceptive and antipyretic effects of isoquinoline alkaloids from the roots of Turkish berberis specie. Life Sci. 72 (6), 645–657. 10.1016/s0024-3205(02)02200-2 12467905

[B43] Laplaza R. Peccati F. Boto R. A. Quan C. Carbo A. Piquemal J. P. (2020). NCIPLOT and the analysis of non-covalent interactions using the reduced density gradient. WIREs Comput. Mol. Sci. 11 (2), 1497. 10.1002/wcms.1497

[B44] Lee C. T. Yang W. T. Parr R. G. (1988). Development of the colle-salvetti correlation-energy formula into a functional of the electron density. Phys. Rev. B Condens Matter 37 (2), 785–789. 10.1103/physrevb.37.785 9944570

[B45] Lesar A. Milošev I. (2009). Density functional study of the corrosion inhibition properties of 1,2,4-triazole and its amino derivatives. Chem. Phy Lett. 483 (4-6), 198–203. 10.1016/j.cplett.2009.10.082

[B46] Lindquist-Kleissler B. Wenger J. S. Johnstone T. C. (2021). Analysis of oxygen-pnictogen bonding with full bond path topological analysis of the electron density. Inorg. Chem. 60 (3), 1846–1856. 10.1021/acs.inorgchem.0c03308 33471517

[B47] Liu S. Schauer C. K. (2015). Origin of molecular conformational stability: perspectives from molecular orbital interactions and density functional reactivity theory. J. Chem. Phys. 142, 054107. 10.1063/1.4907365 25662636

[B48] Lu T. Chen F. (2012). Multiwfn: a multifunctional wavefunction analyzer. J. Comput. Chem. 33 (5), 580–592. 10.1002/jcc.22885 22162017

[B49] Lu R. Gan W. Wu B. Zhang Z. Guo Y. Wang H. (2005). C-H stretching vibrations of methyl, methylene and methine groups at the vapor/alcohol (N = 1-8) interfaces. J. Phys. Chem. B 109 (29), 14118–14129. 10.1021/jp051565q 16852773

[B50] Lu Y. C. Lin Q. Luo G. S. Dai Y. Y. (2006). Solubility of berberine chloride in various solvents. J. Chem. Eng. Data 51 (2), 642–644. 10.1021/je0504360

[B51] Martin J. M. L. Alsenoy V. (1995). Gar2ped, C. Antwerp. Belgium: University of Antwerp.

[B52] Megrouss Y. Baara F. T. Boukabcha N. Chouaih A. Hatzidimitriou A. Djafri A. (2019). Synthesis, X-ray structure determination and related physical properties of thiazolidinone derivative by DFT quantum chemical method. Acta Chim. Slov. 66 (2), 490–500. 10.17344/acsi.2019.5066 33855506

[B53] Millan-Casarrubias E. J. García-Tejeda Y. V. González-De la Rosa H. G. Ruiz-Mazón L. Hernández-Rodríguez Y. M. Cigarroa-Mayorga O. E. (2025). Molecular docking and pharmacological *in silico* evaluation of camptothecin and related ligands as promising HER2-Targeted therapies for breast cancer. Curr. Issues Mol. Biol. 47 (3), 193. 10.3390/cimb47030193 40136447 PMC11941075

[B54] Mir M. A. (2023). DFT, hirshfeld surface, molecular docking and drug likeness studies of medicinally important coumarin molecule. Arabian J. Sci. Eng. 48 (6), 7445–7462. 10.1007/s13369-022-07476-z

[B55] Misik V. Bezakova L. Malekova L. Kostalova D. (1995). Lipoxygenase inhibition and antioxidant properties of protoberberine and aporphine alkaloids isolated from Mahonia aquifolium. Planta Med. 61 (4), 372–373. 10.1055/s-2006-958107 7480190

[B56] Muthu S. Ramachandran S. G. Maheswari J. U. (2012). Vibrational spectroscopic investigation on the structure of 2-ethylpyridine-4-carbothioamide. Spectrochim. Acta A Mol. Biomol. Spectrosc. 93, 214–222. 10.1016/j.saa.2012.02.107 22481178

[B57] Nagy P. (2014). Competing intramolecular vs. intermolecular hydrogen bonds in solution. Int. J. Mol. Sci. 15 (11), 19562–19633. 10.3390/ijms151119562 25353178 PMC4264129

[B58] NIH (2026). 2-Sulfobenzoic Acid. Available online at: https://pubchem.ncbi.nlm.nih.gov/compound/2-Sulfobenzoic-acid.

[B59] Nishio M. (2012). The CH/π hydrogen bond: implication in chemistry. J. Mol. Struct. 1018, 2–7. 10.1016/j.molstruc.2012.03.012

[B60] Origin (Pro) Version (2020). Origin Lab Corporation. MA, USA: Northampton.

[B61] Pal R. Chattaraj P. K. (2021). Chemical reactivity from a conceptual density functional theory perspective. J. Ind. Chem. Soc. 98 (1), 100008. 10.1016/j.jics.2021.100008

[B62] Pan G. Y. Huang Z. J. Wang G. J. Fawcett J. P. Liu X. D. Zhao X. C. (2003). The antihyperglycaemic activity of berberine arises from a decrease of glucose absorption. Planta Med. 69 (7), 632–636. 10.1055/s-2003-41121 12898419

[B63] Panzade P. S. Shendarkar G. R. (2020). Pharmaceutical cocrystal: a game changing approach for the administration of old drugs in new crystalline form. Drug Dev. Ind. Pharm. 46 (10), 1559–1568. 10.1080/03639045.2020.1810270 32799687

[B64] Parr R. G. Szentpály L. V. Liu S. (1999). Electrophilicity index. Chem. Rev. 121 (9), 1922–1924. 10.1021/ja983494x

[B65] Patel N. A. Patel N. J. Patel R. P. Patel R. K. (2010). The formulation and evaluation of topical berberine hydrochloride products. Pharm. Technol. 34 (1), 60–69. Available online at: https://www.pharmtech.com/view/formulation-and-evaluation-topical-berberine-hydrochloride-products.

[B66] Pearson R. G. (2005). Chemical hardness and density functional theory. J. Chem. Sci. 117 (5), 369–377. 10.1007/bf02708340

[B67] Perdew J. P. Wang Y. (1992). Accurate and simple analytic representation of the electron-gas correlation energy. Phys. Rev. B Condens Matter 45 (23), 13244–13253. 10.1103/physrevb.45.13244 10001404

[B68] Petersson G. A. Allaham M. A. (1991). A complete basis set model chemistry. II. Open‐shell systems and the total energies of the first‐row atoms. J. Chem. Phys. 94, 6081–6090. 10.1063/1.460447

[B69] Petersson G. A. Bennett A. Tensfeldt T. G. Allaham M. A. Shirley W. A. Mantzaris J. (1988). A complete basis set model chemistry. I. The total energies of closed-shell atoms and hydrides of the first-row elements. J. Chem. Phys. 89, 2193–2218. 10.1063/1.455064

[B70] Pir N. G. Avci D. Atalay Y. (2012). Molecular structure, vibrational spectra, NLO and NBO analysis of bis (8-Oxy-1-methylquinolinium) hydroiodide. Spectrochim. Acta A Mol. Biomol. Spectrosc. 96, 916–924. 10.1016/j.saa.2012.07.044 22954808

[B71] Politzer P. Murray J. S. (2002). The fundamental nature and role of the electrostatic potential in atoms and molecules. Theor. Chem. Acc. 108 (3), 134–142. 10.1007/s00214-002-0363-9

[B72] Prajapati P. Pandey J. Tandon P. Sinha K. Shimpi M. R. (2022). Molecular structural, hydrogen bonding interactions and chemical reactivity studies of ezetimibe-L-proline cocrystal using a spectroscopic and quantum chemical approach. Front. Chem. 10, 848014. 10.3389/fchem.2022.848014 35242745 PMC8885513

[B73] Pulay P. Fogarasi G. Pang F. Boggs J. E. (1979). Systematic *ab initio* gradient calculation of molecular geometries, force constants, and dipole moment derivatives. J. Am. Chem. Soc. 101 (10), 2550–2560. 10.1021/ja00504a009

[B74] Reddy C. M. Krishna G. R. Ghosh S. (2010). Mechanical properties of molecular crystals-applications to crystal engineering. CrystEngComm 12 (8), 2296–2314. 10.1039/c003466e

[B75] Rejnhardt P. Gajda R. Woińska M. Parafiniuk J. Giester G. Miletich R. (2025). Symmetrization of strong hydrogen bond under high pressure in bihydroxide-ion-containing NaCu2(SO4)2•H3O2 revealed by experimental charge density, single-crystal electron diffraction, and neutron diffraction studies. J. Am. Chem. Soc. 147 (30), 26830–26843. 10.1021/jacs.5c08310 40674283 PMC12314919

[B76] Rijal R. Sah M. Lamichhane H. P. (2023). Molecular simulation, vibrational spectroscopy and global reactivity descriptors of pseudoephedrine molecule in different phases and states. Heliyon 9 (3), 14801–14811. 10.1016/j.heliyon.2023.e14801 PMC10123198 37101481

[B77] Roy P. Pandey N. Ghosh A. (2023). Molecular docking of pharmaceutical cocrystal as a ligand: do we need to rethink? Cryst. Growth Des. 23 (1), 1–5. 10.1021/acs.cgd.2c01379

[B78] Rozas I. Alkorta I. Elguero J. (2000). Behavior of ylides containing N, O, and C atoms as hydrogen bond acceptors. J. Am. Chem. Soc. 122 (45), 11154–11161. 10.1021/ja0017864

[B79] Sadeghian Z. Bayat M. Gheidari D. (2025). Synthesis, molecular docking, pharmacological evaluation, MD simulation, and DFT calculations of quinazolin-12-one derivatives as PDK1 inhibitors. Nanoscale Adv. 7 (18), 5760–5783. 10.1039/d5na00182j 40808821 PMC12341467

[B80] Sahin S. Dege N. (2021). Synthesis, characterization, X-ray, HOMO-LUMO, MEP, FT-IR, NLO, hirshfeld surface, admet, boiled-egg model properties and molecular docking studies with human cyclophilin d (cypd) of a schiff base compound: (e)-1-(5-nitro-2-(piperidin-1-yl)phenyl)-n-(3-nitrophenyl)methanimine. Polyhedron 205 (10), 115320. 10.1016/j.poly.2021.115320

[B81] Saleh G. Gatti C. Lo P. (2012). Non-covalent interaction via the reduced density gradient: independent atom model vs experimental multipolar electron densities. Comput. Theor. Chem. 998, 148–163. 10.1016/j.comptc.2012.07.014

[B82] Sanchez-Chapula J. (1996). Increase in action potential duration and inhibition of the delayed rectifier outward current IK by berberine in cat ventricular myocytes. Br. J. Pharmacol. 117 (7), 1427–1434. 10.1111/j.1476-5381.1996.tb15302.x 8730735 PMC1909453

[B83] Schultheiss N. Newman A. (2009). Pharmaceutical cocrystals and their physicochemical properties. Cryst. Growth Des. 9 (6), 2950–2967. 10.1021/cg900129f 19503732 PMC2690398

[B84] Sert Y. Gumus M. Gokce H. Kani I. Koca I. (2018). Molecular docking, hirshfeld surface, structural, spectroscopic, electronic, NLO and thermodynamic analyses on novel hybrid compounds containing pyrazole and coumarin cores. J. Mol. Struct. 1171, 850–866. 10.1016/j.molstruc.2018.06.069

[B85] Shamina A. H. Jothy V. B. Asif M. Nasibullah M. Kadaikunnan S. Alharbi N. S. (2024). Molecular structure prediction, experimental and theoretical properties, and biological activities of (e)-5-nitro-3-(phenylimino) indolin-2-one-in-vitro against 60 lethal tumour cell lines. J. Mol. Struct. 1305, 137737. 10.1016/j.molstruc.2024.137737

[B86] Spek A. L. (2003). Single-crystal structure validation with the program PLATON. J. Appl. Crystallogr. 36 (1), 7–13. 10.1107/s0021889802022112

[B87] Srivastava A. Karthick T. Joshi B. D. Mishra R. Tandon P. Ayala A. P. (2017). Spectroscopic (far or terahertz, mid-infrared and raman) investigation, thermal analysis and biological activity of piplartine. Spectrochim. Acta A Mol. Biomol. Spectrosc184 184, 368–381. 10.1016/j.saa.2017.05.007 28538206

[B88] Srivastava A. Prajapati P. Kale D. P. Tandon P. Bansal A. K. (2025). Unveiling spectroscopic behaviour and molecular features (DFT studies) of exemestane-maleic acid cocrystal as model multicomponent system. J. Mol. Struct. 1319 (4), 139471. 10.1016/j.molstruc.2024.139471

[B89] Sun A. Yang H. Li T. Luo J. Zhou L. Chen R. (2024). Molecular mechanisms, targets and clinical potential of berberine in regulating metabolism: a review focussing on databases and molecular docking studies. Front. Pharmacol. 15, 1368950. 10.3389/fphar.2024.1368950 38957396 PMC11217548

[B90] Tong H. H. Y. Chow A. S. F. Chan H. M. Chow A. H. L. Wan Y. K. Y. Williams I. D. (2010). Process-induced phase transformation of berberine chloride hydrates. J. Pharm. Sci. 99 (4), 1942–1954. 10.1002/jps.21983 19894277

[B91] Tran Q. L. Tezuka Y. Ueda J. Y. Nguyen N. T. Maruyama Y. Begum K. (2003). *In vitro* antiplasmodial activity of antimalarial medicinal plants used in Vietnamese traditional medicine. J. Ethnopharmacol. 86 (2-3), 249–252. 10.1016/s0378-8741(03)00045-x 12738095

[B92] Tsai P. L. Tsai T. H. (2004). Hepatobiliary excretion of berberine. Drug Metab. Dispos. 32 (4), 405–412. 10.1124/dmd.32.4.405 15039293

[B93] Vijayaraj R. Subramanian V. Chattaraj P. K. (2009). Comparison of global reactivity descriptors calculated using various density functionals: a QSAR perspective. J. Chem. Theory Comput. 5 (10), 2744–2753. 10.1021/ct900347f 26631787

[B94] Wang C. Perumalla S. R. Lu R. Fang J. Sun C. C. (2016). Sweet berberine. Cryst. Growth Des. 16 (2), 933–939. 10.1021/acs.cgd.5b01484

[B95] Wolff S. K. Grimwood D. J. McKinnon J. J. Turner M. J. Jayatilaka D. Spackman M. A. (2012). Crystal Explorer 3.0. Perth: University of Western Australia.

[B96] Xia M. Y. Zhu B. Q. Wang J. R. Yang Z. E. Mei X. F. (2022). Superior dissolution behavior and bioavailability of pharmaceutical cocrystals and recent regulatory issues. ACS Med. Chem. Lett. 13 (1), 29–37. 10.1021/acsmedchemlett.1c00478 35059121 PMC8762724

[B97] Yesilada E. Kupeli E. Berberis Crataegina D. C. (2002). Root exhibits potent anti-inflammatory, analgesic and febrifuge effects in mice and rats. J. Ethnopharmacol. 79 (2), 237–248. 10.1016/s0378-8741(01)00387-7 11801387

[B98] Yoshida H. Takeda K. Okamura J. Ehara A. Matsuura H. (2002). A new approach to vibrational analysis of large molecules by density functional theory: wavenumber-linear scaling method. J. Phys. Chem. A 106 (14), 3580–3586. 10.1021/jp013084m

[B99] Zuo F. Nakamura N. Akao T. Hattori M. (2006). Pharmacokinetics of berberine and its main metabolites in conventional and pseudo germ-free rats determined by liquid chromatography/ion trap mass spectrometry. Drug Metab. Dispos. 34 (12), 2064–2072. 10.1124/dmd.106.011361 16956957

