# Supplementary material for: Synthesis, crystal structure, and characterization of a new crystalline berberine–2-sulfobenzoate salt, integrating vibrational spectroscopy and quantum chemical investigation
Source: Front Chem. 2026 Oct 21;14:1971429. doi: 10.3389/fchem.2026.1971429 (PMC13731199; doi:10.3389/fchem.2026.1971429)

checkCIF/PLATON report

You have not supplied any structure factors. As a result the full set of tests cannot be run.

THIS REPORT IS FOR GUIDANCE ONLY. IF USED AS PART OF A REVIEW PROCEDURE FOR PUBLICATION, IT SHOULD NOT REPLACE THE EXPERTISE OF AN EXPERIENCED CRYSTALLOGRAPHIC REFEREE.

No syntax errors found. CIF dictionary Interpreting this report

Datablock: shelxl

```
Bond precision:  C-C = 0.0032 A          Wavelength=0.71073

Cell:          a=7.4100(6)        b=11.8890(14)     c=15.1030(11)
               alpha=111.976(4)   beta=100.073(4)   gamma=96.880(6)
Temperature:   293 K

                Calculated                 Reported
Volume          1189.6(2)                  1189.57(19)
Space group     P -1                       P -1
Hall group      -P 1                       -P 1
Moiety formula  C20 H18 N O4, C7 H5 O5 S   C20 H18 N1 O4, C7 H5 O5 S1
Sum formula     C27 H23 N O9 S             C27 H23 N O9 S
Mr              537.53                     537.52
Dx,g cm-3       1.501                      1.501
Z               2                          2
Mu (mm-1)       0.197                      0.197
F000            560.0                      560.0
F000'           560.56
h,k,lmax        9,15,19                    0,0,0
Nref            5429                       5222
Tmin,Tmax       0.945,0.961                0.943,0.961
Tmin'           0.943

Correction method= MULTI-SCAN

Data completeness= 0.962          Theta(max)= 27.435

R(reflections)= 0.0535( 4419)     wR2(reflections)= 0.1509( 5222)

S = 1.038                 Npar= Npar = 419
```

The following ALERTS were generated. Each ALERT has the format
test-name_ALERT_alert-type_alert-level.
Click on the hyperlinks for more details of the test.

Alert level C

```
PLAT029_ALERT_3_C _diffrn_measured_fraction_theta_full Low .......      0.974 Note
PLAT030_ALERT_1_C _diffrn_reflns_number   <   _reflns_number_total     Please Check
PLAT241_ALERT_2_C High      Ueq as Compared to Neighbors for .....         C1 Check
PLAT242_ALERT_2_C Low       Ueq as Compared to Neighbors for .....         S1 Check
PLAT242_ALERT_2_C Low       Ueq as Compared to Neighbors for .....        C21 Check
PLAT411_ALERT_2_C Short Inter H...H Contact  H2A    ..  H26     ..       2.12 Ang.
```

Alert level G

```
PLAT066_ALERT_1_G Predicted and Reported Tmin&Tmax Range Identical          ? Check
PLAT180_ALERT_4_G Check Cell Rounding: # of Values Ending with 0 =          4
PLAT194_ALERT_1_G Missing _cell_measurement_reflns_used datum ....     Please Do !
PLAT195_ALERT_1_G Missing _cell_measurement_theta_max   datum ....     Please Do !
PLAT196_ALERT_1_G Missing _cell_measurement_theta_min   datum ....     Please Do !
PLAT199_ALERT_1_G Reported _cell_measurement_temperature ..... (K)        293 Check
PLAT200_ALERT_1_G Reported   _diffrn_ambient_temperature ..... (K)        293 Check
```

---

0 **ALERT level A** = Most likely a serious problem - resolve or explain
0 **ALERT level B** = A potentially serious problem, consider carefully
6 **ALERT level C** = Check. Ensure it is not caused by an omission or oversight
7 **ALERT level G** = General information/check it is not something unexpected

7 ALERT type 1 CIF construction/syntax error, inconsistent or missing data
4 ALERT type 2 Indicator that the structure model may be wrong or deficient
1 ALERT type 3 Indicator that the structure quality may be low
1 ALERT type 4 Improvement, methodology, query or suggestion
0 ALERT type 5 Informative message, check

It is advisable to attempt to resolve as many as possible of the alerts in all categories. Often the minor alerts point to easily fixed oversights, errors and omissions in your CIF or refinement strategy, so attention to these fine details can be worthwhile. In order to resolve some of the more serious problems it may be necessary to carry out additional measurements or structure refinements. However, the purpose of your study may justify the reported deviations and the more serious of these should normally be commented upon in the discussion or experimental section of a paper or in the "special_details" fields of the CIF. checkCIF was carefully designed to identify outliers and unusual parameters, but every test has its limitations and alerts that are not important in a particular case may appear. Conversely, the absence of alerts does not guarantee there are no aspects of the results needing attention. It is up to the individual to critically assess their own results and, if necessary, seek expert advice.

Publication of your CIF in IUCr journals

A basic structural check has been run on your CIF. These basic checks will be run on all CIFs submitted for publication in IUCr journals (*Acta Crystallographica*, *Journal of Applied Crystallography*, *Journal of Synchrotron Radiation*); however, if you intend to submit to *Acta Crystallographica Section C* or *E*, you should make sure that full publication checks are run on the final version of your CIF prior to submission.

Publication of your CIF in other journals

Please refer to the *Notes for Authors* of the relevant journal for any special instructions relating to CIF submission.

PLATON version of 05/02/2014; check.def file version of 05/02/2014

Datablock shelxl - ellipsoid plot

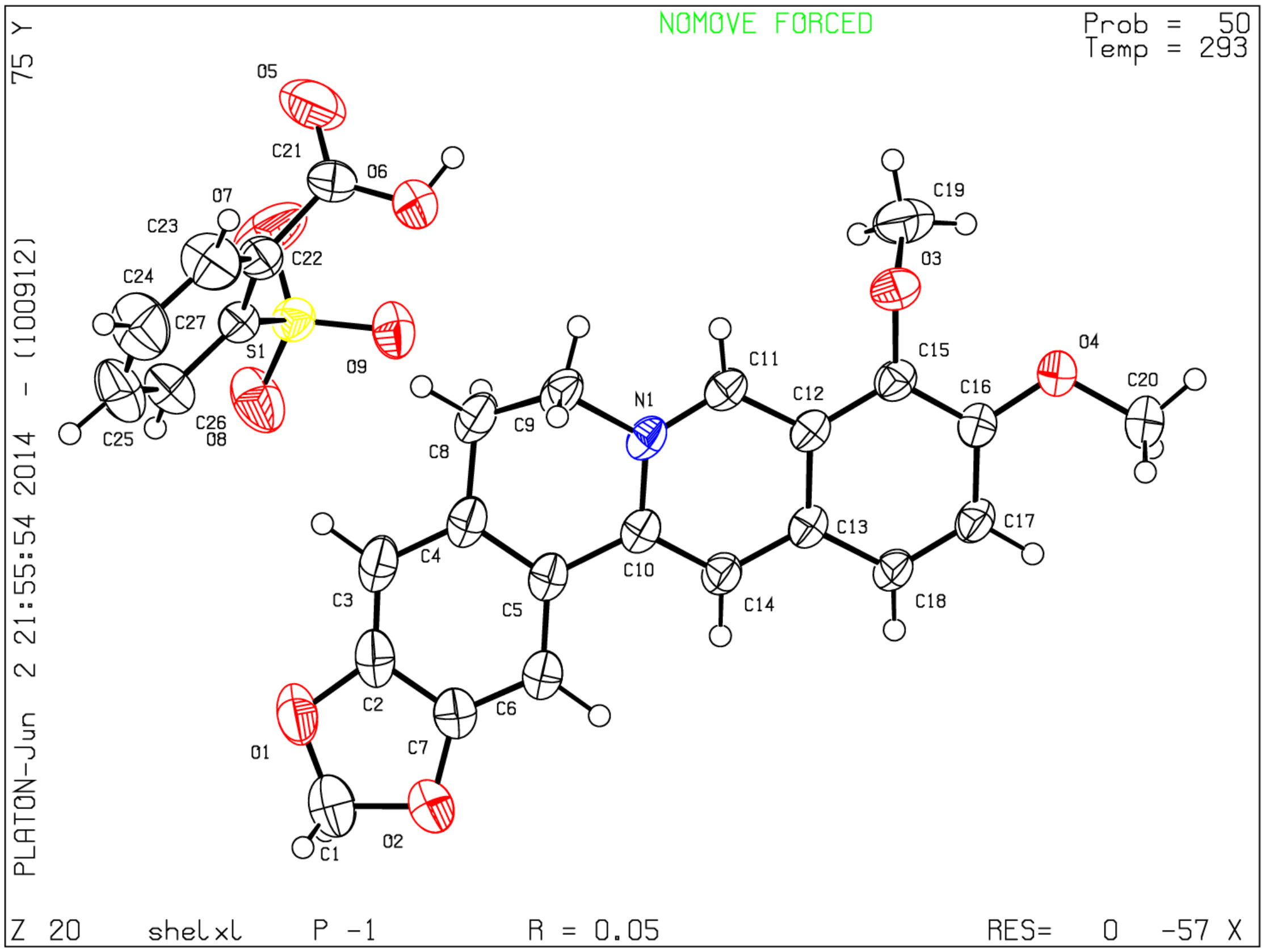

Supplement: Supplementary file 1 [file DataSheet1.pdf]
