# Supplementary material for: Synthesis, crystal structure, and characterization of a new crystalline berberine–2-sulfobenzoate salt, integrating vibrational spectroscopy and quantum chemical investigation
Source: Front Chem. 2026 Oct 21;14:1971429. doi: 10.3389/fchem.2026.1971429 (PMC13731199; doi:10.3389/fchem.2026.1971429)
Supplement: Supplementary file 2 [file DataSheet2.docx]

Supplementary Material

**Synthesis, Crystal Structure and Characterization of a New Crystalline Berberine-2-Sulfobenzoate Salt, Integrating Vibrational Spectroscopy and Quantum Chemical Investigation**

**Anubha Srivastava^1^*, Preeti Prajapati^1^, Manishkumar R. Shimpi^2^*, Poonam Tandon^1,3^***

^1^*Department of Physics, University of Lucknow, Lucknow, 226007, India.

^2*^Chemistryof Interfaces, Luleå University of Technology, Luleå, Sweden.

^3^*Department of Physics, Deen Dayal Upadhyaya Gorakhpur University, Gorakhpur, 273009, India.

*** Correspondence:**

Poonam Tandon: tandon_poonam@lkouniv.ac.in

Manishkumar R. Shimpi: manishkumar.shimpi@astrazeneca.com; manishshimpi@gmail.com

Anubha Srivastava: anubhaphysics@gmail.com

# 1 Supplementary Figures and Tables

XRPD pattern of the received BerCl sample (top) compared with simulated single crystal patterns of BerCldihydrate (middle) and BerCltetrahydrate (bottom) is shown in Fig. S1. Reflections characteristic of both hydrate forms is present in the received BerCl sample, indicating a mixed phase composition. The optical micrographs and structural representations of the crystals used for single crystal data collection is shown in Fig. S2. The optimized structures of BerCland SBA are shown in Figs. S3 and S4, respectively, with atomic numbering adopted in this study. The comparison of experimental and calculated(scaled) Raman scattering spectra of BerCl in the region 100-1000, 1000-1900 and 2600-3300 cm^-1^ is shown in Fig. S5. The comparison of experimental and calculated(scaled) Raman scattering spectra of SBA in the region 100-1900 and 2700-3600 cm^-1^ is shown in Fig. S6. The molecular electrostatic potential (MEP) maps formed by mapping of total density over electrostatic potential in the gas phase for BerCl and SBA are given in Figs. S7 and S8, respectively. The HOMO-LUMO plots with their energy gap (ΔE) for BerCland SBA with orbital involved in electronic transitions are depicted in Figs. S9 and S10, respectively.

Crystallographic data (experimental details) and structure refinement parameters of BER-SBA are shown in Table S1.Characteristics (distances/Å and angles/^o^) of hydrogen bonds in the molecular compounds is given in Table S2.A comparison of the experimental and calculated geometrical parameters of BER-SBA salt using DFT/6-311++G(d,p) basis is given in Table S3; bond lengths in angstroms (Å), bond angles and dihedral angles in degrees (˚). A comparison of theoretical and experimental vibrational wavenumbers (cm^-1^) of BerCl, SBA and BER-SBA saltalong with their assignments using B3LYP/6-311++G(d,p) basis are given in Tables S4, S5 and S6, respectively.The geometrical parameters for intermolecular interaction in BER-SBA salt: bond distance (Å), bond angle (°) and sum of van der Waals radii of interacting atoms (Å) is given in Table S7.The second-order perturbation theory analysis of the Fock matrix, in the NBO basis for intra- and intermolecular interactions in BER-SBA salt is pasted in Table S8. Molecular docking analysis illustrating the binding orientation and key intermolecular interactions of berberine with selected protein targets are presented in Table S9.

## 1.1 Supplementary Figures.

**Fig. S1**XRPD pattern of the received BerCl sample (top) compared with simulated single crystal patterns of BerCldihydrate (middle) and BerCltetrahydrate (bottom). Reflections characteristic of both hydrate forms is present in the received BerCl sample, indicating a mixed phase composition.

**Fig. S2**Optical micrographs and structural representations of the crystals used for single crystal data collection.

**Fig. S3**Optimized structure of BerCl with atomic numbering adopted in this study.

**Fig. S4**Optimized structure of SBA with atomic numbering adopted in this study.

**Fig. S5** Experimental and calculated (scaled) Raman scattering spectra of BerCl in the region 100-1000, 1000-1900 and 2600-3300 cm^-1^.

**Fig. S6**Experimental and calculated(scaled) Raman scattering spectra of SBA in the region 100-1900 and 2700-3600 cm^-1^.

**Fig. S7**Molecular electrostatic potential (MEP) map formed by mapping of total density over electrostatic potential in the gas phase for BerCl.

**Fig. S8**Molecular electrostatic potential (MEP) map formed by mapping of total density over electrostatic potential in the gas phase for SBA.

**Fig. S9**HOMO-LUMO plot with their energy gap (ΔE) for BerCl with orbital involved in electronic transitions.

**Fig. S10**HOMO-LUMO plot with their energy gap (ΔE) for SBA with orbital involved in electronic transitions.

## 1.2 Supplementary Tables

# Table S1. Crystallographic data and structure refinment parameters of BER-SBA.

|  | **BER-SBA** |
| --- | --- |
| empirical formula | C_27_H_23_N_1_O_9_S_1_ |
| formula wt | 537.52 |
| crystal shape | Blocks |
| crystal color | Yellow |
| crystal system | Triclinic |
| space group | *P-1* |
| *a*(Å) | 7.410(6) |
| *b*(Å) | 11.889(1) |
| *c*(Å) | 15.103(1) |
| *α*(°) | 111.97(4) |
| *β*(°) | 100.07(4) |
| *γ*(°) | 96.88(6) |
| *V*(Å^3^) | 1189.6(2) |
| *Z* | 2 |
| *D*_calc_(g cm^-3^) | 1.501 |
| *T* (K) | 293(2) |
| *λ*(Mo_Kα_)[Å] | 0.71073 |
| *μ* (mm^-1^) | 0.197 |
| *F(000)* | 560 |
| No. unique reflns | 5222 |
| No. reflns used | 4419 |
| No. parameters | 419 |
| GOF on *F^2^* | 1.038 |
| *R*1[*I*>2σ(*I*)] | 0.0535 |
| *wR*2 | 0.1509 |
| CCDC No. | 2529913 |

**Table S2**. Characteristics (distances/Å and angles/^o^) of hydrogen bonds in the molecular compounds^$^.

| **BER-SBA** | | | | |
| --- | --- | --- | --- | --- |
| **(D-H^…^A)** | **(D^…^A)** | **(H^…^A)** | **(D-H^…^A)** |  |
| O(6) –H(6O)O(9) | 2.6395 | 1.79 | 170 |  |
| C(8) –H(8B)O(9) | 3.3788 | 2.56 | 146 |  |
| C(11)–H(11)O(7) | 3.1897 | 2.34 | 155 |  |
| C(18)–H(18)O(5) | 3.3417 | 2.54 | 149 |  |
| C(23) –H(23)O(3) | 3.2683 | 2.50 | 141 |  |

^$^The first column represents the atom label for the hydrogen bonds and the three numbers correspond to the distances of H^…^A, D^…^A and angle of D-H^…^A for each interaction.

**Table S3.** The experimental and calculated geometrical parameters of BerCl, SBA and BER-SBA salt using DFT/6-311++G(d,p), bond lengths in angstroms (Å), bond angles and dihedral angles in degrees (˚).

| **Parameters** | **Experimental BerCl** | **Optimized**  **BerCl** | **Experimental SBA** | **Optimized**  **SBA** | **Experimental BER-SBA** | **Optimized BER-SBA** |
| --- | --- | --- | --- | --- | --- | --- |
| **Bond length (Å)** | | | | | | |
| R(S1−O5) | - | - | 1.6340 | 1.6432 | 1.4213 | 1.5776 |
| R(S1−O6) | - | - | 1.4538 | 1.4586 | 1.4370 | 1.6006 |
| R(S1−O7) | - | - | 1.4536 | 1.4463 | 1.4548 | 1.694 |
| R(S1−C18) | - | - | 1.7516 | 1.8090 | 1.7897 | 1.8779 |
| R(O2=C8) | - | - | 1.3606 | 1.2049 | 1.1891 | 1.2357 |
| R(O3-H4) | - | - | 0.9813 | 0.9694 | 0.8630 | 1.0424 |
| R(O3−C8) | - | - | 1.2340 | 1.3432 | 1.2821 | 1.3414 |
| R(H4−O25) | - | - | - | - | 1.7981 | 1.5347 |
| R(O6−H69) | - | - | - | - | 2.6933 | 2.8909 |
| R(O7−H22) | - | - | - | - | 1.7968 | 1.081 |
| R(O7−H69) | - | - | - | - | 2.6751 | 2.0905 |
| R(O7−H71) | - | - | - | - | 2.6666 | 2.5117 |
| R(C8−C9) | - | - | 1.4573 | 1.5019 | 1.5075 | 1.4933 |
| R(C9−C10) | - | - | 1.3950 | 1.3971 | 1.3900 | 1.4008 |
| R(C9−C18) | - | - | 1.3942 | 1.4050 | 1.3911 | 1.3903 |
| R(C10−H11) | - | - | 1.0874 | 1.0829 | 0.9266 | 1.0807 |
| R(C10−C12) | - | - | 1.3949 | 1.3917 | 1.3810 | 1.3924 |
| R(C12−H13) | - | - | 1.0854 | 1.0836 | 0.9410 | 1.0837 |
| R(C12−C14) | - | - | 1.3951 | 1.3909 | 1.3760 | 1.3968 |
| R(C14−H15) | - | - | 1.0850 | 1.0835 | 0.9395 | 1.0836 |
| R(C14−C16) | - | - | 1.3948 | 1.3928 | 1.3721 | 1.3941 |
| R(C16−H17) | - | - | 1.0865 | 1.0820 | 0.8821 | 1.0815 |
| R(C16−C18) | - | - | 1.3949 | 1.3933 | 1.3910 | 1.3886 |
| R(S19−O23) | - | - | - | - | 1.4218 | 1.6899 |
| R(S19−O24) | - | - | - | - | 1.4370 | 1.5861 |
| R(S19−O25) | - | - | - | - | 1.4544 | 1.6004 |
| R(S19−C36) | - | - | - | - | 1.7890 | 1.8379 |
| R(O20−C26) | - | - | - | - | 1.1903 | 1.3099 |
| R(O20−H65) | - | - | - | - | 2.6863 | 2.8306 |
| R(O20−H71) | - | - | - | - | 2.5417 | 2.0825 |
| R(O21−H22) | - | - | - | - | 0.8629 | 1.446 |
| R(O21−C26) | - | - | - | - | 1.2809 | 1.2664 |
| R(C26−C27) | - | - | - | - | 1.5073 | 1.4909 |
| R(C27−C28) | - | - | - | - | 1.3893 | 1.3976 |
| R(C27−C36) | - | - | - | - | 1.3918 | 1.3874 |
| R(C28−H29) | - | - | - | - | 0.9266 | 1.0819 |
| R(C28−C30) | - | - | - | - | 1.3811 | 1.3937 |
| R(C30−H31) | - | - | - | - | 0.9416 | 1.0831 |
| R(C30−C32) | - | - | - | - | 1.3761 | 1.3979 |
| R(C32−H33) | - | - | - | - | 0.9396 | 1.0832 |
| R(C32−C34) | - | - | - | - | 1.3721 | 1.396 |
| R(C34−H35) | - | - | - | - | 0.8819 | 1.0826 |
| R(C34−C36) | - | - | - | - | 1.3910 | 1.3834 |
| R(O37−C39) | 1.4348 | 1.4356 | - | - | 1.4130 | 1.4897 |
| R(O37−C42) | 1.3772 | 1.3717 | - | - | 1.3740 | 1.392 |
| R(N38−C56) | 1.4908 | 1.4679 | - | - | 1.4917 | 1.5025 |
| R(N38−C59) | 1.3992 | 1.4028 | - | - | 1.3893 | 1.4031 |
| R(N38−C60) | 1.3275 | 1.3771 | - | - | 1.3275 | 1.3447 |
| R(C39−H40) | 0.9893 | 1.0890 | - | - | 1.0261 | 1.0895 |
| R(C39−H41) | 1.4342 | 1.4319 | - | - | 1.4269 | 1.4742 |
| R(C39−H43) | 0.9905 | 1.0969 | - | - | 1.0866 | 1.0909 |
| R(O41−C52) | 1.3809 | 1.3757 | - | - | 1.3743 | 1.3967 |
| R(C42−C45) | 1.3646 | 1.3755 | - | - | 1.3591 | 1.3783 |
| R(C42−C52) | 1.3883 | 1.3920 | - | - | 1.3866 | 1.3984 |
| R(O44−C66) | 1.3693 | 1.3654 | - | - | 1.3712 | 1.384 |
| R(C44−C72) | 1.4397 | 1.4348 | - | - | 1.4176 | 1.4838 |
| R(C45−H46) | 0.9502 | 1.0832 | - | - | 0.9854 | 1.0815 |
| R(C45−C48) | 1.4033 | 1.4062 | - | - | 1.4032 | 1.404 |
| R(O47−C67) | 1.3571 | 1.3657 | - | - | 1.3589 | 1.3752 |
| R(O47−C76) | 1.4336 | 1.4223 | - | - | 1.4310 | 1.4827 |
| R(C48−C49) | 1.4027 | 1.4057 | - | - | 1.4003 | 1.4105 |
| R(C48−C53) | 1.5002 | 1.5078 | - | - | 1.4990 | 1.5174 |
| R(C49−C50) | 1.4178 | 1.4165 | - | - | 1.4104 | 1.4188 |
| R(C49−C59) | 1.4727 | 1.4762 | - | - | 1.4743 | 1.4746 |
| R(C50−H51) | 0.9493 | 1.0809 | - | - | 0.9942 | 1.082 |
| R(C50−C52) | 1.3625 | 1.3713 | - | - | 1.3670 | 1.3726 |
| R(C53−H54) | 0.9903 | 1.0929 | - | - | 1.0028 | 1.0945 |
| R(C53−H55) | 0.9896 | 1.0975 | - | - | 0.9349 | 1.098 |
| R(C53−H56) | 1.5102 | 1.5219 | - | - | 1.5034 | 1.5303 |
| R(C56−H57) | 0.9897 | 1.0958 | - | - | 0.9434 | 1.0944 |
| R(C56−H58) | 0.9909 | 1.0911 | - | - | 0.9726 | 1.0913 |
| R(C59−C64) | 1.3750 | 1.3623 | - | - | 1.3752 | 1.3813 |
| R(C60−H61) | 0.9501 | 1.0794 | - | - | 0.9103 | 1.0804 |
| R(C60−C62) | 1.4026 | 1.4581 | - | - | 1.4065 | 1.3921 |
| R(C62−C63) | 1.4202 | 1.4128 | - | - | 1.4209 | 1.4388 |
| R(C62−C66) | 1.4100 | 1.4026 | - | - | 1.4130 | 1.4226 |
| R(C63−C64) | 1.4114 | 1.4340 | - | - | 1.4048 | 1.4135 |
| R(C63−C70) | 1.4141 | 1.4020 | - | - | 1.4150 | 1.4081 |
| R(C64−H65) | 0.9497 | 1.0807 | - | - | 0.9346 | 1.0849 |
| R(C66−C67) | 1.3846 | 1.4057 | - | - | 1.3817 | 1.3968 |
| R(C67−C68) | 1.4133 | 1.4023 | - | - | 1.4105 | 1.4149 |
| R(C68−H69) | 0.9498 | 1.0818 | - | - | 0.9820 | 1.0804 |
| R(C68−C70) | 1.3751 | 1.3863 | - | - | 1.3656 | 1.3767 |
| R(C70−H71) | 0.9505 | 1.0841 | - | - | 0.9002 | 1.0819 |
| R(C72−H73) | 0.9803 | 1.0894 | - | - | 0.9602 | 1.0871 |
| R(C72−H74) | 0.9799 | 1.0899 | - | - | 0.9594 | 1.0899 |
| R(C72−H75) | 0.9793 | 1.0957 | - | - | 0.9606 | 1.0957 |
| R(C76−H77) | 0.9800 | 1.0888 | - | - | 0.9603 | 1.0949 |
| R(C76−H78) | 0.9794 | 1.0948 | - | - | 0.9593 | 1.0895 |
| R(C76−H79) | 0.9809 | 1.0954 | - | - | 0.9607 | 1.0951 |
| **Bond angle (°)** | | | | | | |
| A(O5−S1−O6) | - | - | 107.08 | 106.08 | 114.77 | 116.31 |
| A(O5−S1−O7) | - | - | 108.21 | 107.25 | 112.93 | 112.60 |
| A(O5−S1−C18) | - | - | 105.01 | 103.61 | 106.49 | 116.29 |
| A(O6−S1−O7) | - | - | 120.90 | 121.58 | 110.48 | 105.04 |
| A(O6−S1−C18) | - | - | 107.83 | 107.77 | 105.41 | 101.78 |
| A(O7−S1−C18) | - | - | 106.76 | 109.10 | 106.01 | 103.24 |
| A(H4−O3−C8) | - | - | 111.82 | 107.88 | 110.67 | 114.97 |
| A(S1−O6−H69) | - | - | - | - | 97.09 | 78.20 |
| A(S1−O7−H22) | - | - | - | - | 131.04 | 116.83 |
| A(S1−O7−H69) | - | - | - | - | 97.39 | 104.61 |
| A(S1−O7−H71) | - | - | - | - | 144.61 | 136.10 |
| A(H22−O7−H69) | - | - | - | - | 126.08 | 138.43 |
| A(H22−O7−H71) | - | - | - | - | 76.64 | 88.29 |
| A(H69−O7−H71) | - | - | - | - | 49.89 | 62.20 |
| A(O2−C8−O3) | - | - | 122.30 | 123.98 | 123.11 | 126.14 |
| A(O2−C8−C9) | - | - | 125.33 | 123.42 | 122.66 | 122.38 |
| A(O3−C8−C9) | - | - | 112.36 | 112.49 | 114.22 | 111.48 |
| A(C8−C9−C10) | - | - | 118.42 | 115.78 | 117.08 | 119.34 |
| A(C8−C9−C18) | - | - | 121.56 | 125.99 | 124.01 | 122.13 |
| A(C10−C9−C18) | - | - | 120.02 | 118.23 | 118.86 | 118.52 |
| A(C9−C10−H11) | - | - | 120.71 | 118.23 | 117.49 | 117.47 |
| A(C9−C10−C12) | - | - | 119.99 | 121.12 | 120.98 | 120.71 |
| A(H11−C10−C12) | - | - | 119.30 | 120.65 | 121.50 | 121.82 |
| A(C10−C12−H13) | - | - | 120.01 | 119.72 | 120.25 | 119.93 |
| A(C10−C12−C14) | - | - | 120.00 | 119.97 | 120.12 | 119.88 |
| A(H13−C12−C14) | - | - | 119.99 | 120.31 | 119.51 | 120.19 |
| A(C12−C14−H15) | - | - | 119.99 | 120.51 | 122.58 | 120.49 |
| A(12−C14−C16) | - | - | 119.97 | 119.88 | 119.30 | 119.73 |
| A(H15−C14−C16) | - | - | 120.05 | 119.60 | 117.96 | 119.78 |
| A(C14−C16−H17) | - | - | 118.06 | 120.75 | 119.67 | 122.52 |
| A(C14−C16−C18) | - | - | 120.03 | 119.95 | 121.51 | 119.77 |
| A(H17−C16−C18) | - | - | 121.91 | 119.28 | 118.71 | 117.70 |
| A(S1−C18−C9) | - | - | 121.37 | 123.35 | 122.57 | 126.62 |
| A(S1−C18−C16) | - | - | 118.64 | 115.55 | 118.18 | 112.04 |
| A(C9−C18−C16) | - | - | 119.99 | 120.82 | 119.19 | 121.33 |
| A(O23−S19−O24) | - |  | - | - | 114.73 | 110.32 |
| A(O23−S19−O25) | - | - | - | - | 112.92 | 113.15 |
| A(O23−S19−C36) | - | - | - | - | 106.50 | 100.07 |
| A(O24−S19−O25) | - | - | - | - | 110.46 | 114.32 |
| A(O24−S19−C36) | - | - | - | - | 105.40 | 106.01 |
| A(O25−S19−C36) | - | - | - | - | 106.06 | 111.85 |
| A(C26−O20−H65) | - | - | - | - | 125.51 | 163.31 |
| A(C26−O20−H71) | - | - | - | - | 117.48 | 126.48 |
| A(H65−O20−H71) | - | - | - | - | 57.47 | 59.45 |
| A(H22−O21−C26) | - | - | - | - | 110.75 | 116.14 |
| A(H4−O25−S19) | - | - | - | - | 131.07 | 128.45 |
| A(O20−C26−O21) | - | - | - | - | 123.11 | 121.82 |
| A(O20−C26−C27) | - | - | - | - | 122.60 | 119.78 |
| A(O21−C26−C27) | - | - | - | - | 114.28 | 118.40 |
| A(C26−C27−C28) | - | - | - | - | 117.12 | 118.03 |
| A(C26−C27−C36) | - | - | - | - | 123.97 | 123.48 |
| A(C28−C27−C36) | - | - | - | - | 118.86 | 118.49 |
| A(C27−C28−H29) | - | - | - | - | 117.47 | 117.57 |
| A(27−C28−C30) | - | - | - | - | 121.01 | 120.26 |
| A(C29−C28−C30) | - | - | - | - | 121.49 | 122.16 |
| A(C28−C30−H31) | - | - | - | - | 120.23 | 119.82 |
| A(28−C30−C32) | - | - | - | - | 120.10 | 120.17 |
| A(H31−C30−C32) | - | - | - | - | 119.53 | 120.01 |
| A(C30−C32−H33) | - | - | - | - | 122.62 | 120.35 |
| A(C30−C32−C34) | - | - | - | - | 119.31 | 119.83 |
| A(H33−C32−C34) | - | - | - | - | 117.93 | 119.82 |
| A(C32−C34−H35) | - | - | - | - | 119.66 | 122.79 |
| A(C32−C34−C36) | - | - | - | - | 121.51 | 119.00 |
| A(H35−C34−C36) | - | - | - | - | 118.73 | 118.21 |
| A(S19−C36−C27) | - | - | - | - | 122.58 | 122.57 |
| A(S19−C36−C34) | - | - | - | - | 118.19 | 115.14 |
| A(C27−C36−C34) | - | - | - | - | 119.18 | 122.24 |
| A(C39−O37−C42) | 104.22 | 105.09 | - | - | 105.36 | 105.64 |
| A(C56−N38−C59) | 120.07 | 119.15 | - | - | 118.83 | 118.63 |
| A(C56−N38−C60) | 117.45 | 116.78 | - | - | 118.38 | 119.01 |
| A(C59−N38−C60) | 122.41 | 120.38 | - | - | 122.71 | 122.30 |
| A(O37−C39−H40) | 110.32 | 109.40 | - | - | 107.46 | 108.90 |
| A(O37−C39−O41) | 107.09 | 107.30 | - | - | 109.52 | 106.74 |
| A(O37−C39−H43) | 110.32 | 109.16 | - | - | 113.48 | 108.95 |
| A(H40−C39−O41) | 110.27 | 109.52 | - | - | 107.77 | 109.44 |
| A(H40−C39−H43) | 108.56 | 111.89 | - | - | 115.98 | 112.97 |
| A(O41−C39−H43) | 110.27 | 109.45 | - | - | 102.30 | 109.66 |
| A(C39−O41−C52) | 104.07 | 105.15 | - | - | 104.69 | 106.12 |
| A(O37−C42−C45) | 128.12 | 128.88 | - | - | 128.05 | 127.88 |
| A(O37−C42−C52) | 109.63 | 109.63 | - | - | 109.66 | 110.81 |
| A(C45−C42−C52) | 122.23 | 121.49 | - | - | 122.26 | 121.30 |
| A(C66−O44−C72) | 113.44 | 117.70 | - | - | 115.06 | 118.34 |
| A(C42−C45−H46) | 121.47 | 121.24 | - | - | 123.35 | 120.86 |
| A(C42−C45−C48) | 117.10 | 117.64 | - | - | 117.75 | 117.93 |
| A(H46−C45−C48) | 121.43 | 121.11 | - | - | 118.87 | 121.22 |
| A(C67−O47−C76) | 117.96 | 118.72 | - | - | 117.68 | 118.53 |
| A(C45−C48−C49) | 120.91 | 121.10 | - | - | 120.24 | 120.92 |
| A(C45−C48−C53) | 120.25 | 120.71 | - | - | 120.71 | 120.70 |
| A(C49−C48−C53) | 118.82 | 118.18 | - | - | 119.03 | 118.37 |
| A(C48−C49−C50) | 120.61 | 119.95 | - | - | 120.82 | 120.13 |
| A(C48−C49−C59) | 120.13 | 119.96 | - | - | 119.52 | 119.68 |
| A(C50−C49−C59) | 119.24 | 120.08 | - | - | 119.50 | 120.16 |
| A(C49−C50−H51) | 121.61 | 121.79 | - | - | 122.10 | 122.60 |
| A(C49−C50−C52) | 116.75 | 117.69 | - | - | 117.23 | 117.61 |
| A(H51−C50−C52) | 121.64 | 120.50 | - | - | 120.67 | 119.78 |
| A(O41−C52−C42) | 109.30 | 109.39 | - | - | 110.02 | 110.34 |
| A(O41−C52−C50) | 128.33 | 128.49 | - | - | 128.29 | 127.54 |
| A(C42−C52−C50) | 122.37 | 122.11 | - | - | 121.67 | 122.11 |
| A(C48−C53−H54) | 109.44 | 110.52 | - | - | 114.00 | 110.50 |
| A(C48−C53−H55) | 109.50 | 110.53 | - | - | 109.46 | 110.75 |
| A(C48−C53−C56) | 110.87 | 109.76 | - | - | 111.23 | 108.77 |
| A(H54−C53−H55) | 107.99 | 107.12 | - | - | 106.84 | 108.30 |
| A(H54−C53−C56) | 109.52 | 109.52 | - | - | 106.45 | 108.97 |
| A(H55−C53−C56) | 109.46 | 109.35 | - | - | 108.63 | 109.52 |
| A(N38−C56−C53) | 110.78 | 110.15 | - | - | 110.44 | 109.23 |
| A(N38−C56−H57) | 109.56 | 108.99 | - | - | 104.17 | 107.65 |
| A(N38−C56−H58) | 109.44 | 107.51 | - | - | 104.45 | 107.31 |
| A(C53−C56−H57) | 109.48 | 111.08 | - | - | 110.66 | 110.24 |
| A(C53−C56−H58) | 109.50 | 111.18 | - | - | 117.74 | 112.38 |
| A(H57−C56−H58) | 108.03 | 107.82 | - | - | 108.38 | 109.87 |
| AN(38−C59−C49) | 118.00 | 117.73 | - | - | 118.55 | 118.24 |
| A(N38−C59−C64) | 117.45 | 118.63 | - | - | 117.66 | 117.61 |
| A(C49−C59−C64) | 124.56 | 123.56 | - | - | 123.75 | 124.14 |
| A(N38−C60−H61) | 119.12 | 113.88 | - | - | 114.48 | 118.11 |
| A(N38−C60−C62) | 121.69 | 116.55 | - | - | 121.37 | 121.92 |
| A(H61−C60−C62) | 119.19 | 116.24 | - | - | 124.14 | 119.96 |
| A(C60−C62−C63) | 118.13 | 118.06 | - | - | 117.76 | 117.80 |
| A(C60−C62−C66) | 121.12 | 120.78 | - | - | 121.78 | 120.49 |
| A(C63−C62−C66) | 120.74 | 121.14 | - | - | 120.45 | 121.72 |
| A(C62−C63−C64) | 118.10 | 118.31 | - | - | 118.52 | 118.54 |
| A(C62−C63−C70) | 118.45 | 118.48 | - | - | 118.44 | 118.05 |
| A(C64−C63−C70) | 123.45 | 123.17 | - | - | 123.03 | 123.40 |
| A(C59−C64−C63) | 122.18 | 121.54 | - | - | 121.87 | 121.82 |
| A(C59−C64−H65) | 118.87 | 119.69 | - | - | 120.11 | 119.77 |
| A(C63−C64−H65) | 118.94 | 118.61 | - | - | 118.02 | 118.41 |
| A(O20−H65−C64) | - | - | - | - | 145.80 | 135.05 |
| A(O44−C66−C62) | 119.15 | 118.03 | - | - | 118.73 | 115.34 |
| A(O44−C66−C67) | 121.18 | 122.55 | - | - | 121.60 | 126.27 |
| A(C62−C66−C67) | 119.57 | 119.29 | - | - | 119.55 | 118.36 |
| A(O47−C67−C66) | 115.90 | 116.47 | - | - | 116.56 | 118.22 |
| A(O47−C67−C68) | 124.34 | 124.04 | - | - | 123.61 | 122.49 |
| A(C66−C67−C68) | 119.76 | 119.48 | - | - | 119.82 | 119.28 |
| A(C67−C68−C69) | 119.37 | 119.90 | - | - | 120.80 | 120.48 |
| A(C67−C68−C70) | 121.28 | 120.94 | - | - | 121.48 | 123.02 |
| A(C69−C68−C70) | 119.35 | 119.15 | - | - | 117.72 | 116.48 |
| A(O6−H69−O7) | - | - | - | - | 52.53 | 60.84 |
| A(O6−H69−C68) | - | - | - | - | 154.48 | 144.86 |
| A(O7−H69−C68) | - | - | - | - | 124.78 | 128.21 |
| A(C63−C70−C68) | 120.17 | 120.62 | - | - | 120.22 | 119.55 |
| A(C63−C70−H71) | 119.93 | 119.72 | - | - | 121.33 | 120.15 |
| A(C68−C70−H71) | 119.90 | 119.66 | - | - | 118.44 | 120.30 |
| A(O7−C71−O20) | - | - | - | - | 82.20 | 87.27 |
| A(O7−H71−C70) | - | - | - | - | 128.16 | 109.42 |
| A(O20−H71−C70) | - | - | - | - | 148.78 | 163.29 |
| A(O44−C72−H73) | 109.44 | 105.58 | - | - | 109.43 | 109.95 |
| A(O44−C72−H74) | 109.43 | 111.44 | - | - | 109.49 | 104.59 |
| A(O44−C72−H75) | 109.53 | 110.40 | - | - | 109.45 | 110.10 |
| A(H73−C72−H74) | 109.44 | 110.00 | - | - | 109.46 | 111.72 |
| A(H73−C72−H75) | 109.51 | 109.49 | - | - | 109.49 | 110.09 |
| A(H74−C72−H75) | 109.48 | 109.85 | - | - | 109.52 | 110.26 |
| A(O47−C76−H77) | 109.46 | 105.72 | - | - | 109.52 | 109.86 |
| A(O47−C76−H78) | 109.56 | 111.27 | - | - | 109.54 | 103.93 |
| A(O47−C76−H79) | 109.44 | 111.54 | - | - | 109.46 | 112.91 |
| A(O77−C76−H78) | 109.48 | 109.32 | - | - | 109.46 | 110.02 |
| A(H77−C76−H79) | 109.40 | 109.28 | - | - | 109.39 | 109.30 |
| A(H78−C76−H79) | 109.50 | 109.61 | - | - | 109.46 | 110.70 |
| **Dihedral angle (°)** | | | | | | |
| D(O5−S1−O6−H69) | - | - | - | - | 144.14 | 149.90 |
| D(O7−S1−O6−H69) | - | - | - | - | 15.06 | 24.68 |
| D(C18−S1−O6−H69) | - | - | - | - | -99.03 | -82.67 |
| D(O5−S1−O7−H22) | - | - | - | - | 8.97 | 13.32 |
| D(O5−S1−O7−H69) | - | - | - | - | -145.23 | -163.26 |
| D(O5−S1−O7−H71) | - | - | - | - | -125.19 | 131.83 |
| D(O6−σ1−O7−H22) | - | - | - | - | 139.03 | 140.83 |
| D(O6−S1−O7−H69) | - | - | - | - | -15.18 | -35.75 |
| D(O6−S1−O7−H71) | - | - | - | - | 4.87 | -100.65 |
| D(C18−S1−O7−H22) | - | - | - | - | -107.26 | -112.89 |
| D(C18−S1−O7−H69) | - | - | - | - | 98.53 | 70.54 |
| D(C18−S1−O7−H71) | - | - |  |  | 118.58 | 5.63 |
| D(O5−S1−C18−C9) | - | - | 70.03 | 81.72 | -61.34 | -74.57 |
| D(O5−S1−C18−C16) | - | - | -110.01 | -104.25 | 115.85 | 106.57 |
| D(O6−S1−C18−C9) | - | - | -176.04 | -166.13 | 176.34 | 157.99 |
| D(O6−S1−C18−C16) | - | - | 3.92 | 7.89 | -6.48 | -20.87 |
| D(O7−S1−C18−C9) | - | - | -44.72 | -32.28 | 59.17 | 49.25 |
| D(O7−S1−C18−C16) | - | - | 135.24 | 141.75 | -123.65 | -129.61 |
| D(C8−O3−O25−S19) | - | - | - | - | -174.59 | -66.72 |
| D(H4−O3−C8−O2) | - | - | -179.99 | -7.73 | 2.61 | -6.71 |
| D(H4−O3−C8−C9) | - | - | -0.07 | 176.08 | -176.12 | 173.49 |
| D(S1−O6−H69−O7) | - | - | - | - | -9.60 | -21.98 |
| D(S1−O6−H69−C68) | - | - | - | - | -103.20 | 94.00 |
| D(S1−O7−O21−C26) | - | - | - | - | 174.67 | 116.08 |
| D(H69−O7−O21−C26) | - | - | - | - | -37.56 | -65.92 |
| D(H71−O7−O21−C26) | - | - | - | - | -29.74 | -25.16 |
| D(S1−O7−H69−O6) | - | - | - | - | 9.49 | 20.96 |
| D(S1−O7−H69−C68) | - | - | - | - | 157.93 | -117.86 |
| D(H22−O7−H69−O6) | - | - | - | - | -146.55 | -154.43 |
| D(H22−O7−H69−C68) | - | - | - | - | 1.89 | 66.75 |
| D(H71−O7−H69−O6) | - | - | - | - | -155.47 | 155.74 |
| D(H71−O7−H69−C68) | - | - | - | - | -7.03 | 16.92 |
| D(S1−O7−H71−O20) | - | - | - | - | 171.02 | -110.51 |
| D(S1−O7−H71−C70) | - | - | - | - | -17.03 | 68.61 |
| D(H22−O7−H71−C20) | - | - | - | - | 24.81 | 17.82 |
| D(H22−O7−H71−C70) | - | - | - | - | -163.24 | -163.05 |
| D(H69−O7−H71−O20) | - | - | - | - | -162.59 | 167.33 |
| D(H69−O7−H71−C70) | - | - | - | - | 9.36 | -13.54 |
| D(O2−C8−C9−C10) | - | - | -89.96 | -41.18 | -86.40 | -161.70 |
| D(O2−C8−C9−C18) | - | - | 90.04 | 138.41 | 90.95 | 18.82 |
| D(O3−C8−C9−C10) | - | - | 89.95 | 135.03 | 92.33 | 18.11 |
| D(O3−C8−C9−C18) | - | - | -90.05 | 45.38 | -90.32 | -161.37 |
| D(C8−C9−C10−H11) | - | - | -0.02 | 1.44 | -5.59 | -1.73 |
| D(C8−C9−C10−C12) | - | - | -180.00 | -178.34 | 176.40 | 177.96 |
| D(C18−C9−C10−H11) | - | - | 179.97 | -178.18 | 176.91 | 177.77 |
| D(C18−C9−C10−C12) | - | - | 0.00 | 2.04 | -1.09 | -2.54 |
| D(C8−C9−C18−S1) | - | - | -0.05 | -6.92 | -1.00 | 2.07 |
| D(C8−C9−C18−C16) | - | - | 180.00 | 179.36 | -178.16 | -179.16 |
| D(C10−C9−C18−S1) | - | - | 179.96 | 172.67 | 176.31 | -177.41 |
| D(C10−C9−C18−C16) | - | - | 0.00 | -1.06 | -0.85 | 1.35 |
| D(C9−C10−C12−H13) | - | - | 179.96 | 179.30 | -174.06 | -178.57 |
| D(C9−C10−C12−C14) | - | - | 0.00 | -1.30 | 1.91 | 1.67 |
| D(H11−C10−C12−H13) | - | - | -0.01 | -0.47 | 8.01 | 1.11 |
| D(H11−C10−C12−C14) | - | - | -179.97 | 178.92 | -176.02 | -178.65 |
| D(C10−C12−C14−H15) | - | - | -179.99 | -179.26 | -176.09 | 179.77 |
| D(C10−C12−C14−C16) | - | - | 0.00 | -0.46 | -0.72 | 0.43 |
| D(H13−C12−C14−H15) | - | - | 0.05 | 0.13 | -0.10 | 0.01 |
| D(H13−C12−C14−C16) | - | - | -179.96 | 178.93 | 175.28 | -179.33 |
| D(C12−C14−C16−H17) | - | - | 179.86 | -176.77 | -177.30 | 176.94 |
| D(C12−C14−C16−C18) | - | - | 0.00 | 1.42 | -1.26 | -1.61 |
| D(H15−C14−C16−H17) | - | - | -0.15 | 2.04 | -1.72 | -2.41 |
| D(H15−C14−C16−C18) | - | - | 179.99 | -179.77 | 174.33 | 179.05 |
| D(C14−C16−C18−S1) | - | - | 179.96 | -174.84 | -175.23 | 179.64 |
| D(C14−C16−C18−C9) | - | - | 0.00 | -0.65 | 2.05 | 0.71 |
| D(H17−C16−C18−S1) | - | - | 0.19 | 3.37 | 0.85 | 1.03 |
| D(H17−C16−C18−C9) | - | - | -179.86 | 177.56 | 178.13 | -177.90 |
| D(O23−S19−O25−H4) | - | - | - | - | -9.02 | -25.34 |
| D(O24−S19−O25−H4) | - | - | - | - | -139.01 | -152.76 |
| D(C36−S19−O25−H4) | - | - | - | - | 107.26 | 86.76 |
| D(O23−S19−C36−C27) | - | - | - | - | 61.41 | 49.92 |
| D(O23−S19−C36−C34) | - | - | - | - | -115.83 | -132.67 |
| D(O24−S19−C36−C27) | - | - | - | - | -176.30 | 164.62 |
| D(O24−S19−C36−C34) | - | - | - | - | 6.46 | -17.97 |
| D(O25−S19−C36−C27) | - | - | - | - | -59.13 | -70.17 |
| D(O25−S19−C36−C34) | - | - | - | - | 123.64 | 107.24 |
| D(H65−O20−C26−O21) | - | - | - | - | 103.25 | 120.87 |
| D(H65−O20−C26−C27) | - | - | - | - | -75.38 | -59.90 |
| D(H71−O20−C26−O21) | - | - | - | - | 35.18 | 15.49 |
| D(H71−O20−C26−C27) | - | - | - | - | -143.44 | -165.28 |
| D(C26−O20−H65−C64) | - | - | - | - | -126.19 | -112.59 |
| D(H71−O20−H65−C64) | - | - | - | - | -23.65 | 3.23 |
| D(C26−O20−H71−O7) | - | - | - | - | -47.48 | -30.83 |
| D(C26−O20−H71−C70) | - | - | - | - | 144.78 | 152.03 |
| D(H65−O20−H71−O7) | - | - | - | - | -163.90 | 167.93 |
| D(H65−O20−H71−O70) | - | - | - | - | 28.37 | -9.21 |
| D(H22−O21−C26−O20) | - | - | - | - | -2.59 | 16.39 |
| D(H22−O21−C26−C27) | - | - | - | - | 176.14 | -162.85 |
| D(O20−C26−C27−C28) | - | - | - | - | 86.40 | 171.14 |
| D(O20−C26−C27−C36) | - | - | - | - | -90.94 | -8.90 |
| D(O21−C26−C27−C28) | - | - | - | - | -92.34 | -9.61 |
| D(O21−C26−C27−C36) | - | - | - | - | 90.33 | 170.36 |
| D(C26−C27−C28−H29) | - | - | - | - | 5.59 | 1.01 |
| D(C26−C27−C28−C30) | - | - | - | - | -176.50 | -179.34 |
| D(C36−C27−C28−H29) | - | - | - | - | -176.94 | -178.96 |
| D(C36−C27−C28−C30) | - | - | - | - | 0.97 | 0.70 |
| D(C26−C27−C36−S19) | - | - | - | - | 0.99 | -3.46 |
| D(C26−C27−C36−C34) | - | - | - | - | 178.19 | 179.31 |
| D(C28−C27−C36−S19) | - | - | - | - | -176.30 | 176.51 |
| D(C28−C27−C36−C34) | - | - | - | - | 0.91 | -0.72 |
| D(C27−C28−C30−H31) | - | - | - | - | 174.03 | -179.92 |
| D(C27−C28−C30−C32) | - | - | - | - | -1.74 | -0.01 |
| D(C29−C28−C30−H31) | - | - | - | - | -8.14 | -0.27 |
| D(C29−C28−C30−C32) | - | - | - | - | 176.09 | 179.64 |
| D(C28−C30−C32−H33) | - | - | - | - | 176.09 | 179.60 |
| D(C28−C30−C32−C34) | - | - | - | - | 0.59 | -0.68 |
| D(H31−C30−C32−H33) | - | - | - | - | 0.28 | -0.49 |
| D(H31−C30−C32−C34) | - | - | - | - | -175.22 | 179.23 |
| D(C30−C32−C34−H35) | - | - | - | - | 177.47 | 179.65 |
| D(C30−C32−C34−C36) | - | - | - | - | 1.33 | 0.66 |
| D(H33−C32−C34−H35) | - | - | - | - | 1.76 | -0.63 |
| D(H33−C32−C34−C36) | - | - | - | - | -174.38 | -179.62 |
| D(C32−C34−C36−S19) | - | - | - | - | 175.25 | -177.38 |
| D(C32−C34−C36−C27) | - | - | - | - | -2.08 | 0.05 |
| D(H35−C34−C36−S19) | - | - | - | - | -0.93 | 3.59 |
| D(H35−C34−C36−C27) | - | - | - | - | -178.26 | -178.99 |
| D(C42−O37−C39−H40) | 142.76 | 136.79 | - | - | -125.02 | -123.37 |
| D(C42−O37−C39−O41) | 22.72 | 18.05 | - | - | -8.22 | -5.31 |
| D(C42−O37−C39−H43) | -97.31 | -100.47 | - | - | 105.38 | 113.03 |
| D(C39−O37−C42−C45) | 168.06 | 169.33 | - | - | -177.39 | -177.85 |
| D(C39−O37−C42−C52) | -13.27 | -11.23 | - | - | 4.51 | 2.82 |
| D(C59−N38−C56−C53) | 38.16 | 43.43 | - | - | 42.54 | 43.32 |
| D(C59−N38−C56−C57) | -82.74 | -78.68 | - | - | -76.31 | -76.38 |
| D(C59−N38−C56−C58) | 158.99 | 164.72 | - | - | 170.05 | 165.40 |
| D(C60−N38−C56−C53) | -144.67 | -158.14 | - | - | -140.41 | -139.30 |
| D(C60−N38−C56−H57) | 94.43 | 79.76 | - | - | 100.74 | 101.00 |
| D(C60−N38−C56−H58) | -23.84 | -36.85 | - | - | -12.89 | -17.21 |
| D(C56−N38−C59−C49) | -3.78 | -5.92 | - | - | -7.13 | -4.09 |
| D(C56−N38−C59−C64) | 176.16 | 177.10 | - | - | 175.07 | 176.82 |
| D(C60−N38−C59−C49) | 179.20 | -163.56 | - | - | 175.95 | 178.61 |
| D(C60−N38−C59−C64) | -0.86 | 19.46 | - | - | -1.85 | -0.47 |
| D(C56−N38−C60−H61) | 5.10 | 31.08 | - | - | 4.20 | 2.73 |
| D(C56−N38−C60−C62) | -175.02 | 170.69 | - | - | -174.39 | -176.62 |
| D(C59−N38−C60−H61) | -177.80 | -170.76 | - | - | -178.86 | -179.98 |
| D(C59−N38−C60−C62) | 2.07 | -31.15 | - | - | 2.54 | 0.66 |
| D(O37−N39−O41−C52) | -23.50 | -18.05 | - | - | 8.71 | 5.86 |
| D(H40−C39−O41−C52) | -143.56 | -136.70 | - | - | 125.32 | 123.56 |
| D(H43−C39−O41−C52) | 96.56 | 100.28 | - | - | -111.94 | -112.01 |
| D(C39−O41−C52−C42) | 15.37 | 11.25 | - | - | -5.85 | -4.29 |
| D(C39−O41−C52−C50) | -165.53 | -169.67 | - | - | 175.83 | 176.72 |
| D(O37−C42−C45−H46) | -0.55 | -1.45 | - | - | 3.12 | 0.55 |
| D(O37−C42−C45−C48) | 179.49 | 178.90 | - | - | -178.84 | -179.55 |
| D(C52−C42−C45−H46) | -179.07 | 179.17 | - | - | -178.99 | 179.82 |
| D(C52−C42−C45−C48) | 0.97 | -0.48 | - | - | -0.95 | -0.28 |
| D(O37−C42−C52−O41) | -1.36 | 0.01 | - | - | 0.92 | 0.93 |
| D(O37−C42−C52−C50) | 179.47 | -179.15 | - | - | 179.36 | 179.99 |
| D(C45−C42−C52−O41) | 177.40 | 179.50 | - | - | -177.32 | -178.45 |
| D(C45−C42−C52−C50) | -1.76 | 0.34 | - | - | 1.13 | 0.60 |
| D(C72−O44−C66−C62) | 104.34 | 122.84 | - | - | 106.31 | 138.46 |
| D(C72−O44−C66−C67) | -79.37 | -61.39 | - | - | -77.70 | -43.45 |
| D(C66−O44−C72−H73) | -67.19 | 67.17 | - | - | 69.44 | 55.39 |
| D(C66−O44−C72−H74) | 172.75 | -173.42 | - | - | -170.60 | 175.47 |
| D(C66−O44−C72−H75) | 52.83 | -55.18 | - | - | -50.55 | -66.09 |
| D(C42−C45−C48−C49) | 0.53 | -0.30 | - | - | -0.39 | -0.16 |
| D(C42−C45−C48−C53) | -177.92 | 179.25 | - | - | -178.65 | -179.06 |
| D(H46−C45−C48−C49) | -179.43 | -179.95 | - | - | 177.74 | 179.74 |
| D(H46−C45−C48−C53) | 2.12 | -0.41 | - | - | -0.53 | 0.83 |
| D(C76−O47−C67−C66) | -174.48 | -174.67 | - | - | -166.43 | -174.60 |
| D(C76−O47−C67−C68) | 5.89 | 4.32 | - | - | 13.23 | 5.63 |
| D(C67−O47−C76−H77) | -59.66 | 58.65 | - | - | 50.33 | 66.73 |
| D(C67−O47−C76−H78) | -179.55 | 177.23 | - | - | 170.39 | -175.58 |
| D(C67−O47−C76−H79) | 60.40 | -64.09 | - | - | -69.59 | -55.56 |
| D(C45−C48−C49−C50) | -1.31 | 1.22 | - | - | 1.59 | 0.31 |
| D(C45−C48−C49−C59) | -179.89 | -177.99 | - | - | -173.81 | -177.44 |
| D(C53−C48−C49−C50) | 177.16 | -178.33 | - | - | 179.88 | 179.24 |
| D(C53−C48−C49−C59) | -1.41 | 2.45 | - | - | 4.49 | 1.49 |
| D(C45−C48−C53−H54) | -24.67 | -24.15 | - | - | -30.16 | -24.02 |
| D(C45−C48−C53−H55) | 93.52 | 94.26 | - | - | 89.42 | 96.00 |
| D(C45−C48−C53−C56) | -145.63 | -144.84 | - | - | -150.53 | -143.60 |
| D(C49−C48−C53−H54) | 156.84 | 155.41 | - | - | 151.55 | 157.05 |
| D(C49−C48−C53−H55) | -84.97 | -86.18 | - | - | -88.87 | -82.94 |
| D(C49−C48−C53−C56) | 35.88 | 34.72 | - | - | 31.18 | 37.47 |
| D(C48−C49−C50−H51) | -179.41 | 177.11 | - | - | 177.90 | -178.43 |
| D(C48−C49−C50−C52) | 0.56 | -1.33 | - | - | -1.41 | -0.01 |
| D(C59−C49−C50−H51) | -0.82 | 3.68 | - | - | -6.71 | -0.70 |
| D(C59−C49−C50−C52) | 179.15 | 177.88 | - | - | 173.99 | 177.73 |
| D(C48−C49−C59−N38) | -15.97 | -18.66 | - | - | -17.76 | -20.01 |
| D(C48−C49−C59−C64) | 164.10 | 158.15 | - | - | 159.89 | 159.01 |
| D(C50−C49−C59−N38) | 165.44 | 162.13 | - | - | 166.78 | 162.25 |
| D(C50−C49−C59−C64) | -14.49 | -21.06 | - | - | -15.56 | -18.73 |
| D(C49−C50−C52−O41) | -178.07 | -178.41 | - | - | 178.22 | 178.44 |
| D(C49−C50−C52−C42) | 0.93 | 0.58 | - | - | 0.08 | -0.44 |
| D(H51−C50−C52−O41) | 1.90 | 3.13 | - | - | -1.09 | -3.09 |
| D(H51−C50−C52−C42) | -179.10 | -177.89 | - | - | -179.23 | 178.03 |
| D(C48−C53−C56−N38) | -52.38 | -56.20 | - | - | -52.93 | -57.95 |
| D(C48−C53−C56−H57) | 68.55 | 64.66 | - | - | 61.90 | 60.14 |
| D(C48−C53−C56−H58) | -173.18 | -175.27 | - | - | -172.72 | -176.93 |
| D(H54−C53−C56−N38) | 173.34 | 177.61 | - | - | -177.66 | -178.48 |
| D(H54−C53−C56−H57) | 65.86 | 65.29 | - | - | -62.84 | -60.39 |
| D(H54−C53−C56−H58) | -52.40 | 63.33 | - | - | 62.55 | 62.54 |
| D(H55−C53−C56−N38) | 68.45 | -53.77 | - | - | 67.62 | 63.21 |
| D(H55−C53−C56−H57) | -170.61 | -173.84 | - | - | -177.56 | -178.69 |
| D(H55−C53−C56−H58) | -52.35 | -56.74 | - | - | -52.17 | -55.76 |
| D(N38−C59−C64−C63) | -1.27 | 0.36 | - | - | -1.02 | -0.48 |
| D(N38−C59−C64−H65) | 178.71 | 175.67 | - | - | 178.33 | 179.25 |
| D(C49−C59−C64−C63) | 178.66 | -176.43 | - | - | -178.69 | -179.50 |
| D(C49−C59−C64−H65) | -1.36 | -1.12 | - | - | 0.65 | 0.23 |
| D(N38−C60−C62−C63) | -1.11 | 23.57 | - | - | -0.35 | 0.10 |
| D(N38−C60−C62−C66) | 177.47 | -158.08 | - | - | 178.27 | 179.64 |
| D(H61−C60−C62−C63) | 178.76 | 162.22 | - | - | -178.81 | -179.25 |
| D(H61−C60−C62−C66) | -2.65 | -19.43 | - | - | -0.19 | 0.30 |
| D(C60−C62−C63−C64) | -0.93 | -5.13 | - | - | -2.35 | -0.99 |
| D(C60−C62−C63−C70) | 178.62 | 176.94 | - | - | 178.24 | -179.73 |
| D(C66−C62−C63−C64) | -179.52 | 176.52 | - | - | 179.01 | 179.46 |
| D(C66−C62−C63−C70) | 0.02 | -1.40 | - | - | -0.39 | 0.73 |
| D(C60−C62−C66−O44) | -0.45 | 0.09 | - | - | -0.99 | -1.02 |
| D(C60−C62−C66−C67) | -176.80 | -175.82 | - | - | -177.07 | -179.28 |
| D(C63−C62−C66−O44) | 178.10 | 178.39 | - | - | 177.60 | 178.51 |
| D(C63−C62−C66−C67) | 1.75 | 2.48 | - | - | 1.52 | 0.25 |
| D(C62−C63−C64−C59) | 2.13 | -6.93 | - | - | 3.07 | 1.20 |
| D(C62−C63−C64−H65) | -177.85 | 177.70 | - | - | -176.29 | -178.53 |
| D(C70−C63−C64−C59) | -177.39 | 170.89 | - | - | -177.56 | 179.86 |
| D(C70−C63−C64−H65) | 2.62 | -4.48 | - | - | 3.09 | 0.13 |
| D(C62−C63−C70−C68) | -1.19 | 0.20 | - | - | -1.16 | -1.31 |
| D(C62−C63−C70−H71) | 178.79 | 179.86 | - | - | 177.67 | 178.11 |
| D(C64−C63−C70−C68) | 178.34 | 178.01 | - | - | 179.46 | -179.97 |
| D(C64−C63−C70−H71) | -1.69 | 2.04 | - | - | -1.71 | -0.55 |
| D(C59−C64−C65−O20) | - | - | - | - | -161.32 | 178.06 |
| D(C63−C64−C65−O20) | - | - | - | - | 18.05 | -2.20 |
| D(O44−C66−C67−O47) | 1.72 | 1.36 | - | - | 2.61 | 1.52 |
| D(O44−C66−C67−C68) | -178.63 | -177.68 | - | - | -177.07 | -178.70 |
| D(C62−C66−C67−O47) | 177.99 | 177.07 | - | - | 178.57 | 179.56 |
| D(C62−C66−C67−C68) | -2.36 | -1.96 | - | - | -1.10 | -0.66 |
| D(O47−C67−C68−H69) | 0.87 | 1.18 | - | - | 0.06 | 1.05 |
| D(O47−C67−C68−C70) | -179.17 | -178.55 | - | - | 179.89 | 179.84 |
| D(C66−C67−C68−H69) | -178.75 | 179.86 | - | - | 179.71 | -178.72 |
| D(C66−C67−C68−C70) | 1.22 | 0.41 | - | - | -0.46 | 0.07 |
| D(C67−C68−H69−O6) | - | - | - | - | -99.21 | 68.74 |
| D(C67−C68−C69−O7) | - | - | - | - | -173.88 | 161.10 |
| D(C70−C68−C69−O6) | - | - | - | - | 80.96 | -110.13 |
| D(C70−C68−H69−O7) | - | - | - | - | 6.28 | -17.77 |
| D(C67−C68−C70−C63) | 0.59 | 0.68 | - | - | 1.61 | 0.95 |
| D(C67−C68−C70−H71) | -179.38 | -179.37 | - | - | -177.25 | -178.47 |
| D(H69−C68−C70−C63) | -179.45 | -179.05 | - | - | -178.56 | 179.78 |
| D(H69−C68−C70−H71) | 0.58 | 0.90 | - | - | 2.59 | 0.36 |
| D(H63−C70−H71−O7) | - | - | - | - | 170.54 | -167.64 |
| D(C63−C70−H71−O20) | - | - | - | - | -24.99 | 9.33 |
| D(C68−C70−H71−O7) |  | - | - | - | -10.62 | 11.77 |
| D(C68−C70−H71−O20) |  | - | - | - | 153.86 | -171.26 |

**Table S4.** Theoretical and experimental vibrational wavenumbers (cm^-1^) of BerCl and their assignments using B3LYP/6-311++G(d,p).

| Scaled  wavenumber | Experimental | Potential Energy Distribution (≥5%) |
| --- | --- | --- |
|  | FT-Raman |  |
| 3080 | 3098 | R2[ν(C11H)](98) |
| 3078 | 3073 | R4[ν(C22H)](74)+R2[ν(C15H)](25) |
| 3067 | -- | R2[ν(C15H)](72)+R4[ν(C22H)](25) |
| 3065 | -- | R1[ν(C27H)](91)+R1[ν(C19H)](6) |
| 3053 | -- | R3[ν(C29H)](99) |
| 3037 | 3019 | R1[ν(C19H)](92)+R1[ν(C27H)](7) |
| 3004 | 3009 | [ν_as_(C37H_3_)](99) |
| 3001 | 3001 | [ν_as_(C41H_3_)](98) |
| 2991 | -- | R5[ν_as_(C34H_2_)](99) |
| 2972 | -- | [ν_as_(C37H_3_)](99) |
| 2964 | 2955 | R3[ν_as_(C31H_2_)](99) |
| 2947 | -- | R3[ν_as_(C24H_2_)](98) |
| 2941 | -- | [ν_as_(C41H_3_)](100) |
| 2906 | 2909 | R3[ν_s_(C31H_2_)](93)+R3[ν_s_(C24H_2_)](7) |
| 2892 | -- | [ν(C37H_3_)](99) |
| 2886 | 2877 | R5[ν_s_(C34H_2_)](87)+R3[ν_s_ (C24H_2_)](9) |
| 2885 | 2849 | [ν_s_(C41H_3_)](93) |
| 2885 | -- | R3[ν_s_(C24H_2_)](82)+R5[ν_s_(C34H_2_)](7)+R3[ν(C31H_2_)](5) |
| 1631 | 1633 | R4[ν(C18C)](51)+R2[ν(C10C15)](10)+R4[δa](6)+R4[δin(CH)](7) |
| 1624 | 1626 | R4[ν(CC)](25)+R4,R5[ν(C18C21)](16)+R3,R4[ν(C9C14)](12)+  R2[ν(C10C15)](11)+R4[δa](5) |
| 1613 | -- | R1[ν(CC)](24)+R2[ν(CC)](16)+R4[ν(C18C29)](11)+R1[ν(C13C17)](6)+R2[δa](5)+R1[δa](5)+R4,R5[ν(C18C21)](5) |
| 1607 | 1594 | R1[ν(CC)](41)+R1[δin(CH)](10)+R2[ν(C10C15)](8)+  R4,R5[ν(C18C21)](6)+R1[δa’](6)+R4[ν(C18C29)](5) |
| 1559 | 1570 | R1[ν(CC)](28)+R1,R2[ν(C7C8)](13)+R2[ν(C10C15)](10)+  R1[ν(C13C17)](8)+R1[δa’](5) |
| 1514 | 1518 | R5[(δ(C34H_2_)](85) |
| 1505 | 1501 | R1[δin(C27H)](24)+R1[ν(CC)](22)+R1[ν(C17O)](8)+  R1,R2[ν(C7C8)](7)+R2[ν(C8C15)](7)+[δs(C41H_3_)](5) |
| 1491 | -- | R4[δin(C22H)](19)+R4[ν(CC)](15)+R3[(δ(C31H_2_)](15)+  R5[(δ(C34H_2_)](8)+R4,R5[ν(C18C21)](6)+R5[ν(C18O)](5) |
| 1489 | 1488 | R3[(δ(C31H_2_)](48)+[δa(C37H_3_)](6)+[δa’(C37H_3_)](6) |
| 1488 | -- | [δa(C37H_3_)](47)+[δa’(C37H_3_)](28)+[ρ(C37H_3_)](7)+R3[(δ(C31H_2_)](5) |
| 1481 | -- | [δa’(C41H_3_)](70)+[δa(C41H_3_)](14)+[ρ(C41H_3_)](11) |
| 1470 | -- | [δa(C41H_3_)](76)+[δa’(C41H_3_)](16)+[ρ’(C41H_3_)](6) |
| 1465 | 1464 | R3[(δ(CH_2_)](18)+[δs(C37H_3_)](8)+R2[ν(CC)](8)+R2,R3[ν(NC10)](5)+  R1[ν(C7C13)](5)+R3[ν(C9C10)](5) |
| 1461 | -- | [δa’(C37H_3_)](51)+[δa(C37H_3_)](35)+[ρ’(C37H_3_)](5) |
| 1458 | -- | [δs(C37H_3_)](28)+R3[(δ(C26H_2_)](25)+[δs(C41H_3_)](24) |
| 1457 | -- | R3[(δ(C26H_2_)](61)+R4[ν(CC)](9)+[δs(C41H_3_)](7)+[δs(C37H_3_)](5) |
| 1450 | 1449 | [δs(C41H_3_)](52)+[δs(C37H_3_)](20) |
| 1438 | 1425 | R4[ν(CC)](13)+[δs(C37H_3_)](10)+R2[ν(C7C11)](7)+R2[(ω(C11ClH)](6)+R3[δtri](5)+R1[ν(C7C13)](5)+R5[(ω(C34H_2_)](5) |
| 1414 | -- | R1[ν(CC)](15)+[δs(C37H_3_)](11)+R3[(ω(C31H_2_)](8)+R1[δin(C19H)](8)+R5[(ω(C34H_2_)](6)+R2,R3[ν(NC10)](5)+R2[δin(C15H)](5)+R2[δtri](5) |
| 1402 | -- | R5[(ω(C34H_2_)](69)+R4[ν(C21C22)](6) |
| 1391 | 1397 | R3[(ω(C31H_2_)](19)+R3,R4[ν(C9C14)](15)+R4[ν(CC)](12)+  R2[ν(NC10)](6)+R2[ν(C8C15)](5) |
| 1377 | 1364 | R2[ν(NC10)](18)+R2[ν(CC)](10)+R2[(ω(C11ClH)](9)+  R3,R4[ν(C9C14)](8)+R3[(ω(C31H_2_)](6)+R1[ν(C7C13)](5) |
| 1348 | 1351 | R3[(ω(C31H_2_)](30)+R3[(γ(C31H_2_)](15)+R1[ν(CC)](10)+  R3[ν(C24C31)](6)+R3[ν(C14C24)](5) |
| 1343 | 1343 | R3[(ω(CH_2_)](49)+R4[ν(C9C22)](6)+R2[ν(NC10)](5) |
| 1327 | 1333 | R1[ν(CC)](37)+R1,R2[ν(C7C8)](10)+R1[δin(C19H)](8)+  R2[ν(C8C15)](7)+R1[ν(C13O)](5) |
| 1318 | 1303 | R1[ν(CC)](30)+R3[(γ(C31H_2_)](9)+R2[ν(NC10)](8)+R2[δin(C15H)](6)+  R2[ν(C8C15)](6) |
| 1289 | -- | R1[ν(C17O)](16)+R4[ν(C14C29)](8)+R3[ν(C9C10)](7)+  R1[ν(C17C27)](5)+R3[(γ(C31H_2_)](5) |
| 1280 | 1276 | R1[ν(C17O)](17)+R1[δin(C27H)](10)+R4[ν(C14C29)](8)+R1[δtri](7)+  R3[ν(C9C10)](6)+R4,R5[ν(C18C21)](5) |
| 1259 | -- | R1[δtri](18)+R2[δin(C15H)](14)+R2[ν(C7C11)](10)+R1[ν(C13O)](9)+  R2[(γ(C11ClH)](6)+R2[(ω(C11ClH)](6) |
| 1252 | 1236 | R4[δin(CH)](57)+R3[(γ(C26H_2_)](11) |
| 1239 | -- | R4[ν(CC)](17)+R3[(γ(C31H_2_)](8)+R4,R5[ν(C18C21)](7)+  R5[ν(C18O)](7)+R3,R4[ν(C9C14)](5) |
| 1224 | -- | R2[δin(C15H)](11)+R1[ν(C13O)](9)+R3[(γ(CH_2_)](9)+R1[δin(C27H)](7)+[ρ(C37H_3_)](6)+R1[ν(C8C19)](5) |
| 1214 | -- | R3[(γ(CH_2_)](32)+R3[ν(NC31)](11)+R2[δin(CH)](10)+R2[δtri](6) |
| 1211 | 1204 | R3[(γ(CH_2_)](18)+R1[δin(C19H)](14)+R1[ν(CC)](12)+R5[(γ(C34H_2_)](10)+R2[ν(C7C11)](6)+[ρ(C41H_3_)](5) |
| 1200 | -- | R5[(γ(C34H_2_)](69)+R4[δin(C22H)](7) |
| 1196 | 1183 | R4[δin(C29H)](12)+R3[(γ(C31H_2_)](11)+R2[ν(NC10)](9)+  R3[ν(C9C10)](7)+[ρ(C41H_3_)](7)+R3[ν(NC31)](6)+[ρ(C37H_3_)](5) |
| 1189 | 1176 | [ρ(C37H_3_)](49)+[ρ(C41H_3_)](26)+R1[δin(C27H)](7) |
| 1177 | -- | [ρ(C41H_3_)](30)+[ρ(C37H_3_)](13)+R1[δin(CH)](9)+R1[ν(C19C27)](5) |
| 1170 | -- | R4[δtri](26)+R3[ν(C14C24)](17)+R4[δin(CH)](15)+R5[ν(C21O)](9)+  R4[ν(C9C22)](9)+R5[(γ(C34H_2_)](6) |
| 1162 | -- | R3[ν(NC31)](24)+R2[δtri](12)+R3[(γ(C26H_2_)](10)+R3[ν(C24C31)](7)+  R4[ν(C14C29)](5) |
| 1156 | 1145 | [ρ’(C37H_3_)](93) |
| 1156 | -- | [ρ’(C41H_3_)](94) |
| 1131 | -- | R5[(ρ(C34H_2_)](89) |
| 1120 | 1119 | R1[δin(CH)](16)+[ν(C41O)](9)+[ν(C37O)](8)+R1[ν(C13O)](7)+  R1[ν(C19C27)](6) |
| 1102 | 1104 | [ν(C41O)](20)+R5[ν(C18O)](9)+R4[δtri](9)+R5[ν(C21O)](7) |
| 1078 | -- | R2[(δ(C11ClH)](49)+R2[(ρ(C11ClH)](14)+[ν(C37O)](8) |
| 1074 | -- | R3[ν(CC)](19)+ν(C41O)](18)+R5[ν(C21O)](7)+R5[ν(C18O)](7)+  R2[(δ(C11ClH)](6)+[ν(C37O)](6) |
| 1061 | 1070 | R3[(ρ(CH_2_)](48)+R3[puck](7)+R3[ν(C24C31)](7)+R3[(γ(C26H_2_)](6) |
| 1049 | 1048 | R5[ν(C34O)](37)+R5[ν(C34O5)](29)+R5[δ](7)+R4,R5[ν(C18C21)](6)+  R5[δ’](5) |
| 1007 | 1002 | [ν(C37O)](32)+R3[ν(C24C31)](11)+R1[δtri](10)+R2[δtri](7)+  [ν(C41O)](7)+R3[(p(C31H_2_)](5) |
| 979 | 978 | R3[ν(C24C31)](24)+[ν(C37O)](19)+R3[(ρ(CH_2_)](9) |
| 964 | 954 | [ν(C41O)](13)+R5[ν(C18O)](7)+R2[δtri](6)+R2,R3[ν(NC10)](6)+  R4[δa](5)+R3[ν(C14C24)](5) |
| 946 | -- | R5[ν(C34O5)](36)+R5[ν(C34O)](32)+R5[δ’](6) |
| 932 | 930 | R1[δoop(CH)](84)+R1[puck](8) |
| 922 | 912 | R3[(ρ(CH_2_)](31)+R3[δtri](11)+R5[ν(C34O)](9)+R5[ν(C34O5)](8) |
| 891 | 896 | [ν(C37O)](7)+R1,R2[ν(C7C8)](7)+R2[ν(C7C11)](7)+  R3[(ρ(C31H_2_)](5)+R4[δoop(C22H)](5)+R4[δtri](5) |
| 878 | 889 | R4[δoop(CH)](76)+R4[τa’](5) |
| 863 | 862 | R4[δoop(CH)](79)+R4[puck](15) |
| 850 | 849 | R2[δoop(CH)](61)+R2[puck](13) |
| 835 | 837 | R4,R5[ν(C18C21)](13)+R5[ν(C21O)](11)+R4[ν(CC)](11)+  R2[δoop(C15H)](10)+R4[δa’](6)+R5[ν(C18O)](5) |
| 801 | 803 | R1[δoop(CH)](41)+R1[δoop(C17O)](14)+R1[puck](14)+  R2[δoop(C15H)](10)+R1[δoop(C13O)](8)+R1[τa’](5) |
| 790 | -- | R1[puck](41)+R1[δoop(C13O)](15)+R2[puck](9)+R1[δoop(C19H)](6)  +R1[δoop(C17O)](5) |
| 778 | 782 | R1[δtri](21)+R1[ν(C17O)](11)+R2[δtri](10)+R1[δa’](7)+  R3[ν(NC31)](5) |
| 758 | 771 | R5[δ’](14)+R4[δtri](13)+R3[δtri](6)+R4[puck](6)+R1[δtri](5)+  R3[ν(C14C24)](5) |
| 749 | 753 | R5[δ’a](14)+R3[ν(NC31)](14)+ R3[ν(CC)](12)+R2,R3[ν(NC10)](8)+  +R3,R4[ν(C9C14)](5) |
| 737 | -- | R4[puck](37)+R3[puck](10)+R2[δa’](6)+R3[τa](5) |
| 731 | 731 | R5[δa](48)+R4[δa’](12)+R5[ν(C34O5)](6) |
| 728 | -- | R2[δa’](16)+R5[δ’](14)+R4[puck](14)+R2,R3[ν(NC10)](6) |
| 713 | 713 | R2[puck](23)+R3[δtri](6)+R3[puck](5)+R3[(p(C26H_2_)](5) |
| 707 | 698 | R4[τa](24)+R4[puck](15)+R5[τ](11)+R2[puck](10)+R3[puck](8)+  R5[τ’](5) |
| 676 | 661 | R1[τa’](9)+R1[δoop(C13O)](8)+R2[puck](7)+R2[τa](6)+R4[τa](6)+  R1[δoop(C17O)](5)+R2[δa’](5)+R1[δ(C13OC)](5) |
| 651 | 646 | R2[puck](11)+R1[δin(C17O)](10)+R1[δ(C17OC)](6)+R1[ν(C17C27)](6)+R1[δoop(C17O)](5)+R4[τa](5)+R1[δin(C13O)](5) |
| 621 | 618 | R2[δa’](12)+R2[τa](10)+R1[τa’](9)+R4[δa](8)+R1[δoop(C17O)](8)  +R3[δa](6) |
| 589 | 578 | R1[τa](22)+R1[δoop(C17O)](14)+R1[puck](14)+R1,R2[τ(C7C8)](9)+  R2[(p(C11ClH)](6)+R1[δoop(C13O)](5) |
| 569 | 562 | R4[τa](11)+R3[δtri](10)+R2[τa’](9)+R4[puck](6)+R2[puck](5) |
| 553 | 554 | R3[δa’](22)+R3[δa](10)+R1[δa](9)+R2,R3[τ(C10N)](5) |
| 540 | 537 | R3[δa](16)+R1[δa](15)+R2[δa](10)+R1[δin(C13O)](7)+  R2[(p(C11ClH)](6)+R2[τa’](5)+R3[δa’](5) |
| 518 | 524 | R1[δa’](27)+R1[δ(C17OC)](10)+R3[δa](6)+R3[δa’](5)+R2[δa](5) |
| 470 | 462 | R2,R3[τ(C10N)](17)+R2[τa](10)+R2[(p(C11ClH)](10)+  R3,R4[τ(C9C14)](8)+R4[τa’](6)+R4[δa](5)+R1[τa](5) |
| 448 | 441 | R4[τa’](11)+R2[τa](9)+R2[(p(C11ClH)](9)+R2[τa’](8)+  R3,R4[τ(C9C14)](8)+R1[τa](6)+R4[τa](5) |
| 442 | -- | R4[δa’](22)+R4[δa](19)+R2,R3[τ(C10N)](8)+R4[τa’](5) |
| 425 | 425 | R2[δa](19)+R1[δ(C17OC)](14)+R1[τa](8)+R2[τa](5) |
| 400 | 400 | R4,R5[τ(C18C21)](25)+R4[τa’](16)+R3[δa’](9)+R4[τa](8)+R4[δa’](5) |
| 393 | 396 | R4,R5[τ(C18C21)](18)+R4[τa’](10)+R1,R2[τ(C7C8)](10)+R4[τa](8)+  R1[δoop(C17O)](7)+R1[puck](6) |
| 381 | 382 | R1[δoop(C17O)](13)+R4,R5[τ(C18C21)](9)+R1[δ(C13OC)](8)+  R2,R3[τ(C10N)](6)+R2[τa](6)+R3[δa’](6)+R1[τa’](5) |
| 351 | -- | R4,R5[τ(C18C21)](21)+R3,R4[τ(C9C14)](13)+R1,R2[τ(C7C8)](7) |
| 340 | 337 | R1[δin(C13O)](16)+R1[δ(C17OC)](8)+R4,R5[τ(C18C21)](7)+  R3,R4[τ(C9C14)](7)+R1,R2[τ(C7C8)](5) |
| 294 | 295 | R3[puck](22)+R5[τ](14)+R1[τa](11)+R4[puck](8)+R2[puck](5) |
| 286 | 288 | R4,R5[τ(C18C21)](12)+R1[δoop(C13O)](10)+R1[δ(C13OC)](8)+  R3,R4[τ(C9C14)](7) |
| 280 | 275 | R3[δa](10)+R1[δin(C13O)](7)+R2[(p(C11ClH)](5)+R4[δa](5) |
| 253 | -- | [τ(C41O)](44)+R3[τa’](8)+R1,R2[τ(C7C8)](7)+R2[τa](5)+R5[τ](5)+  R1[τa’](5) |
| 242 | 239 | R4,R5[τ(C18C21)](16)+R3[τa](10)+R2[ν(C11Cl)](7)+  R2[(p(C11ClH)](7)+R1,R2[τ(C7C8)](7)+R4[τa](6)+R1[τ(C13O)](5) |
| 221 | -- | R1[τa](12)+[τ(C41O)](12)+R4,R5[τ(C18C21)](10)+R3[τa’](9)+  R2[ν(C11Cl)](9)+R5[τ’](7)+R3[τa](6) |
| 201 | 198 | R1[δin(C17O)](16)+R1[δin(C13O)](15)+R1[δ(C17OC)](10)+  R2[ν(C11Cl)](6) |
| 198 | -- | R2[δa’](10)+R1[τ(C13O)](10)+[τ(C37O)](7)+R1[δin(C17O)](6)+  R2[δa](5) |
| 171 | 177 | R2[ν(C11Cl)](27)+R5[τ’](17)+R5[τ](13)+R2[(p(C11ClH)](8)  +R2[(δ(C11ClH)](5) |
| 149 | 152 | [τ(C37O)](12)+R1[τa’](12)+R3,R4[τ(C9C14)](10)+R5[τ’](7)+R4[τa](6)+R4,R5[τ(C18C21)](5)+R2[τa](5)+R2[ν(C11Cl)](5) |
| 144 | -- | R2[ν(C11Cl)](27)+R2[(p(C11ClH)](10)+R5[τ’](7)+[τ(C37O)](7)+  R2[τa](6)+R2[(δ(C11ClH)](5)+R3,R4[τ(C9C14)](5) |
| 136 | -- | R2[ν(C11Cl)](15)+R5[τ](14)+R3[τa’](14)+R5[τ’](9)+R4[τa](6)+  R3,R4[τ(C9C14)](5)+R2[τa](5) |
| 120 | -- | R5[τ’](27)+R5[τ](17)+R2[ν(C11Cl)](12)+R1[τa’](5)+R2[τa’](5) |
| 109 | 112 | R2[ν(C11Cl)](20)+R5[τ’](18)+R5[τ](13)+R2[(δ(C11ClH)](7) |
| 103 | -- | R1[τ(C17O)](29)+R3,R4[τ(C9C14)](9)+[τ(C41O)](9)+R3[τa](5)+  R2[τa](5)+R4[τa](5) |
| 88 | -- | R2,R3[τ(C10N)](24)+R2[τa](17)+R2[puck](9)+R5[τ’](6)+  R2[(δ(C11ClH)](6)+R4[τa](5) |
| 73 | -- | R1[τ(C13O)](30)+[τ(C37O)](13)+R1,R2[τ(C7C8)](6)+  R2[(p(C11ClH)](5) |
| 69 | -- | R1[τ(C13O)](21)+R1[τ(C17O)](17)+R3[τa](11)+R3,R4[τ(C9C14)](9)+  [τ(C41O)](7)+R4[τa](6) |
| 49 | -- | R1[τ(C13O)](47)+R1[δ(C13OC)](11)+[τ(C37O)](9)+R1[δin(C13O)](8)+R1[τ(C17O)](5) |
| 40 | -- | R2[τa](19)+R2[ν(C11Cl)](13)+R2,R3[τ(C10N)](10)+R2[(p(C11ClH)](8)+R1[τ(C17O)](7)+R2[τa’](5)+R2[(δ(C11ClH)](5) |
| 28 | -- | R2,R3[τ(C10N)](41)+R3[τa’](16)+R1,R2[τ(C7C8)](11) |

**Table S5.** Theoretical and experimental vibrational wavenumbers (cm^-1^) of SBA and their assignments using B3LYP/6-311++G(d,p).

| **Scaled**  **wavenumber** | **Experimental** | **Potential Energy Distribution (≥5%)** |
| --- | --- | --- |
|  | **FT-Raman** |  |
| 3569 | -- | [ν(O2H)](100) |
| 3561 | -- | [ν(O5H)](100) |
| 3071 | 3091 | R[ν(CH)](96) |
| 3063 | 3079 | R[ν(CH)](97) |
| 3053 | 3070 | R[ν(CH)](100) |
| 3041 | 3036 | R[ν(CH)](98) |
| 1762 | 1727 | [ν(C13=O6)](80)+[ν(O5C13)](5)+R[ν(C8C13)](5) |
| 1602 | 1592 | R[ν(CC)](67)+R[δin(CH)](18)+R[δa’](6) |
| 1580 | 1577 | R[ν(CC)](67)+R[δin(CH)](15)+R[δa](6) |
| 1479 | 1479 | R[δin(CH)](49)+R[ν(CC)](41) |
| 1446 | 1445 | R[δin(CH)](63)+R[ν(CC)](31) |
| 1355 | 1405 | [ν(O5C13)](22)+[δ(C13OH)](22)+R[ν(C8C13)](17)+[ρ(C13O5)](13) |
| 1331 | -- | [ν(S1=O)](62)+R[ν(CC)](14)+[δ(S1OH)](5) |
| 1309 | 1301 | R[ν(CC)](83)+R[ν(C10C12)](10)+[ν(S1=O)](5) |
| 1276 | 1278 | R[δin(CH)](59)+R[ν(CC)](22)+R[δin(C8C13)](5)+R[δin(S1C7)](5) |
| 1196 | 1201 | [δ(C13OH)](44)+R[ν(CC)](19)+[ν(O5C13)](10)+R[δin(C9H)](7) |
| 1179 | 1176 | R[δin(CH)](80)+R[ν(CC)](18) |
| 1142 | 1150 | [ν(S1=O)](34)+ R[ν(CC)](21)+R[δin(CH)](13)+[δ(S1OH)](11)+  [ν(S1C7)(10) |
| 1129 | 1135 | [ν(S1=O)](22)+[δ(S1OH)](18)+R[ν(CC)](21)+R[δin(CH)](20) |
| 1121 | -- | [ν(O5C13)](39)+R[ν(CC)](18)+R[δin(CH)](9)+[δ(C13OH)](8)+  [δ(S1OH)](6) |
| 1095 | 1079 | [δ(S1OH)](54)+[ν(S1=O)(33) |
| 1055 | 1048 | R[ν(CC)](46)+R[δtri](22)+R[δin(CH)](10)+ν(O5C13)](7) |
| 1041 | 1019 | R[δtri](43)+[ν(S1C7)(12)+R[ν(CC)](30)+[ν(S1=O)(8) |
| 1003 | 992 | R[δoop(CH)](82)+R[puck](14) |
| 980 | -- | R[δoop(CH)](90)+R[τa’](6) |
| 900 | -- | R[δoop(CH)](81)+R[puck](7) |
| 804 | 803 | [ω(C13O5)](29)+R[δoop(CH)](20)+R[δoop(C8C13)](17)  +R[puck](12)+R[δoop(C9H)](5) |
| 790 | 793 | R[δa](16)+R[ν(C8C13)](14)+R[δtri](12)+R[δoop(CH)](9)+  R[δa’](8)+[ν(S1C7)(7)+[ν(S1−O)](5)+[ρ(C13O5)](5) |
| 763 | -- | R[δoop(CH)](44)+R[puck](14)+[ω(C13O5)](11)+R[δoop(S1C7)](7)  +R[δoop(C8C13)](6) |
| 756 | 756 | [ν(S1−O)](55)+[ω(S1=O)](13)+R[δoop(CH)](8) |
| 720 | 725 | R[puck](56)+R[δoop(S1C7)](10)+R[δoop(C8C13)](8)+  [ω(C13O5)](6) |
| 687 | 702 | R[δa](25)+R[puck](16)+[ν(S1−O)](13)+[ρ(C13O5)](10) |
| 637 | 626 | R[δa’](49)+[ρ(C13O5)](33)+[δ(C13OH)](5) |
| 589 | -- | [τ(C13O5)](61)+R[puck](13) |
| 573 | 570 | [ω(S1=O)](35)+[δsci(S1=O)](22)+R[δa](15) |
| 559 | -- | R[puck](16)+[δ(C13=O6C8)](15)+R[τa’](10)+R[τa](10)+  R[δoop(C8C13)](9)+R[δin(C8C13)](7) |
| 524 | 539 | R[τa](29)+R[δoop(S1C7)](24)+[ω(S1=O)](14)+[δsci(S1C7O2)](6)+  R[δoop(C12H)](5) |
| 470 | 485 | [ρ(S1=O)](24)+[δsci(S1=O)](18)+R[δin(S1C7)](10)+R[τa](10)+  R[δoop(C8C13)](5) |
| 435 | 431 | R[τa](25)+[δsci(S1=O)](18)+R[δoop(S1C7)](13)+R[τa’](10)+  [ρ(S1=O)](10)+[δsci(S1C7O2)](5) |
| 402 | 405 | R[τa’](30)+[γ( S1=O)](20)+[ρ(S1=O)](11)+R[δoop(C8C13)](7)  +R[ν(C8C13)](5) |
| 385 | 364 | R[τa’](39)+R[δoop(C8C13)](8)+[ρ(C13O5)](7)+R[ν(C8C13)](6)+  [ν(S1C7)(5)+[δ(C13=O6C8)](5)+R[δa’](5) |
| 341 | 320 | [γ(S1=O)](19)+R[τa’](12)+[δ(C13=O6C8)](9)+[ν(S1C7)(8)+  R[δin(S1C7)](7)+[τ(S1O2)](6)+[δsci(S1=O)](5) |
| 293 | 287 | R[τa](20)+[δsci(S1C7O2)](15)+[δsci(S1=O)](11)+[ν(S1C7)(10)+  [γ(S1=O)](8)+R[δoop(C8C13)](8)+R[δa](6) |
| 255 | 258 | [δsci(S1C7O2)](20)+R[τa](19)+[ν(S1C7)(12)+R[δoop(C8C13)](7)+  R[δa](7)+[γ(S1=O)](6) |
| 227 | 214 | [τ(S1−O)](36)+R[δin(S1C7)](17)+[ρ(S1=O)](15)+R[δin(C8C13)](11)+[δ(C13=O6C8)](5) |
| 203 | 201 | [τ(S1−O)](31)+[γ( S1=O)](22)+[δsci(S1C7O2)](17)+  R[δin(C8C13)](9)+[δsci(S1=O)](5) |
| 155 | 174 | R[δin(C8C13)](18)+R[δoop(C8C13)](18)+R[δin(S1C7)](16)+  R[δoop(S1C7)](10)+[ω(C13O5)](6)+R[τ(C8C13)](6)+[γ( S1=O)](5) |
| 148 | 128 | R[δoop(C8C13)](25)+R[δoop(S1C7)](21)+[δsci(S1C7O2)](14)+  [δ(C13=O6C8)](9)+R[τa’](6)+R[δin(C8C13)](6) |
| 100 | -- | R[τ(S1C)](34)+R[τ(C8C13)](20)+R[τa](12)+R[δoop(C8C13)](10)+  R[δoop(S1C7)](8)+R[τa’](5) |
| 86 | -- | R[δoop(S1C7)](26)+[δsci(S1C7O2)](19)+R[τ(S1C)](11)+  R[δin(S1C7)](10)+R[δin(C8C13)](6)+[ρ(S1=O)](5)+[δsci(S1=O)](5) |
| 46 | -- | R[τ(C8C13)](58)+R[τ(S1C)](25)+R[δin(S1C7)](6) |

**Table S6.** Theoretical and experimental vibrational wavenumbers (cm^-1^) of BER-SBA salt and their assignments using B3LYP/6-311++G(d,p).

| **Scaled wavenumber** | **Experimental** | **Potential Energy Distribution (≥5%)** |
| --- | --- | --- |
|  | **FT-Raman** |  |
| 3113 | -- | R4[ν(CH)](99) |
| 3109 | -- | R[ν(C10H)](94) |
| 3107 | -- | R1[ν(C68H)](65)+[δ(C68HS1)](31) |
| 3099 | -- | R[ν(CH)](96) |
| 3096 | -- | R[ν(C16H)](97) |
| 3090 | -- | R[ν(CH)](90) |
| 3090 | -- | R4[ν(C45H)](96) |
| 3088 | 3071 | R1[ν(C70H)](69)+[ν(C20H71)](21) |
| 3079 | -- | R[ν(CH)](96) |
| 3079 | -- | R4[ν(C50H)](84)+R1[ν(C70H)](6) |
| 3075 | -- | R[ν(CH)](97) |
| 3067 | 3062 | R[ν(CH)](97) |
| 3064 | -- | [ν_as_(C72H_3_)](94) |
| 3060 | -- | R[ν(CH)](96) |
| 3049 | 3048 | R2[ν(C64H)](93)+R4[ν(C50H)](5) |
| 3035 | -- | [ν_as_(C76H_3_)](99) |
| 3021 | 3020 | R5[ν_as_(C39H_2_)](100) |
| 3015 | -- | R3[ν_as_(C56H_2_)](99) |
| 3014 | 3010 | [ν_as_(C72H_3_)](100) |
| 2986 | -- | [ν_as_(C76H_3_)](99) |
| 2984 | 2977 | R3[ν_as_(C53H_2_)](94) |
| 2958 | -- | R5[ν_s_(C39H_2_)](100) |
| 2956 | 2943 | R3[ν_s_(CH_2_)](99) |
| 2930 | -- | [ν_s_(C72H_3_)](100) |
| 2928 | 2908 | R3[ν_s_(C53H_2_)](98) |
| 2920 | -- | [ν(O3H)](68)+[ν(O21H)](20) |
| 2904 | -- | [ν(O21H)](75)+[ν(O3H)](18) |
| 2854 | 2843 | [ν_s_(C76H_3_)](99) |
| 1684 | 1704 | [ν(C8=O2)](66)+[ν(C8O3)](9)+[δ(O2=C8C9)](8)+R[δin(C8C)](6) |
| 1619 | 1634 | R4[ν(CC)](50)+R4[δa](9)+R4[δin(CH)](11)+R5[δ’a](5) |
| 1608 | -- | R1[δin(C70H)](45)+[τ(C70HC26C)](17)+[τ(C68HS1C)](9)+  [δ(C20C70C63)](5) |
| 1603 | 1625 | [ν(C26=O20)](51)+R[ν(CC)](25)+R[δin(CH)](10)+R[δa](8)+  R[δin(C36S)](5) |
| 1600 | -- | R[ν(CC)](54)+R[δin(CH)](15)+R[δa](9)+[ρ’(S1O_3_)](7)+  R[δin(C18S)](5) |
| 1598 | -- | R1[δin(CH)](58)+[τ(C70HC26C)](25) |
| 1596 | 1595 | R1[δin(C70H)](22)+R4[ν(CC)](18)+R4,R5[ν(C42C52)](10)+  [τ(C68HS1C)](7)+[τ(C70HC26C)](6)+R3,R4[ν(C48C49)](6) |
| 1582 | -- | R[ν(CC)](49)+R[δin(C26C)](13)+R[δa’](9)+  R[δin(C32H)](7) |
| 1575 | 1570 | R[ν(CC)](58)+R[δin(C14H)](11)+R[δa’](11) |
| 1566 | -- | [δ’as(C76H_3_)](39)+R1[δin(CH)](27)+[τ(C68HS1C)](14)+  [τ(C70HC26C)](7)+[τ(C68HS1O5)](5) |
| 1563 | -- | R5[δ(C39H_2_)](87) |
| 1558 | -- | [δ’as(C72H_3_)](52)+[δas(C72H_3_)](37)+[ρ’(C72H_3_)](5) |
| 1546 | -- | [δ(C20C70C63)](24)+R1[δin(CH)](15)+R1[δa’](9)+R1[ν(CC)](8)+  [τ(C68HS1C)](7) |
| 1541 | -- | [δas(C76H_3_)](45)+R1[δin(C70H)](23)+[τ(C70HC26C)](11)+  [δs(C76H_3_)](6) |
| 1525 | -- | R1[δin(CH)](59)+[τ(C70HC26C)](22)+[τ(C68HS1C)](9) |
| 1520 | 1517 | R3[δ(C56H_2_)](22)+R1[δin(CH)](22)+[δ(C20C70C63)](7)+  [τ(C70HC26C)](6)+[τ(C68HS1C)](5) |
| 1519 | -- | R1[δin(CH)](37)+[ν(C26=O20)](11)+[δas(C72H_3_)](9)+[τ(C70HC26C)](7)+[δ’as(C72H_3_)](7) |
| 1514 | -- | R1[δin(CH)](48)+[τ(C70HC26C)](28)+[τ(C68HS1C)](8) |
| 1505 | -- | R1[δin(C70H)](43)+[τ(C70HC26C)](26)+R3[δ(C53H_2_)](16) |
| 1501 | -- | R1[δin(C70H)](50)+[τ(C70HC26C)](28) |
| 1495 | 1499 | R[δin(CH)](37)+R[ν(CC)](21)+R[δin(C28H)](7) |
| 1494 | -- | R1[δin(C70H)](33)+[τ(C70HC26C)](17)+R1[δin(C68H)](11)+  [τ(C68HS1C)](9)+[δs(C76H_3_)](5) |
| 1493 | -- | R[δin(CH)](24)+R1[δin(C70H)](11)+R[ν(CC)](13)+[τ(C70HC26C)](9) |
| 1490 | 1480 | R1[δin(CH)](45)+[τ(C68HS1C)](20)+[τ(C70HC26C)](15)+  [τ(C68HS1O5)](6) |
| 1474 | -- | R1[δin(C70H)](26)+[δs(C72H_3_)](24)+[τ(C70HC26C)](16)+  [δ(C20C70C63)](10) |
| 1472 | -- | R[δin(CH)](42)+R[ν(CC)](18)+R1[δin(C70H)](6)+[τ(C70HC26C)](6)+  R[δin(C26C)](5)+[ν(C26=O20)](5) |
| 1469 | -- | R[δin(CH)](60)+R[ν(CC)](24)+R[δin(C8C)](5) |
| 1447 | 1447 | R1[δin(C70H)](20)+R4[ν(CC)](20)+[τ(C70HC26C)](13)+R3[δtri](7) |
| 1430 | -- | [δ(C8O3H)](43)+[ν(C8O3)]16)+R[ν(C8C)](12)+[ρ(C8=O2)](11) |
| 1426 | 1422 | R[δin(CH)](51)+[τ(C70HC26C)](37) |
| 1403 | -- | R1[δin(C70H)](23)+R1[ν(CC)](19)+[τ(C70HC26C)](18)+  R1,R2[ν(C62C63)](8)+[δ(C20C70C63)](6) |
| 1393 | 1395 | R3[ω(C56H_2_)](26)+R1[δin(C68H)](19)+[τ(C68HS1C)](10)+  R1[ν(C62C66)](6)+R3,R4[ν(C48C49)](5) |
| 1387 | -- | R5[ω(C39H_2_)](30)+R3[ω(C56H_2_)](20)+R1[δin(C70H)](6)+  R3,R4[ν(C48C49)](5)+R5[ν(O37C42)](5) |
| 1375 | 1368 | R1[δin(CH)](28)+[τ(C70HC26C)](18)+RR5[ω(C39H_2_)](14) |
| 1367 | -- | R1[δin(C70H)](51)+[τ(C70HC26C)](40) |
| 1365 | 1350 | R1[δin(C70H)](42)+[τ(C70HC26C)](31)+R3[γ(C56H_2_)](6) |
| 1339 | -- | [τ(C68HS1C)](17)+R1[δin(C68H)](15)+[δ(C20C70C63)](9)+  R2[δin(CH)](8) |
| 1331 | -- | R1[δin(CH)](30)+[τ(C68HS1C)](20)+[τ(C70HC26C)](10)+  [δ(C20C70C63)](8)+R1[δtri](7)+[τ(C68HS1O5)](5) |
| 1329 | 1337 | R[ν(CC)](43)+R1[δin(CH)](11)+[τ(C68HS1C)](9) |
| 1319 | 1320 | R[ν(CC)](66)+[ρ’(S1O_3_)](7) |
| 1317 | -- | [ν(C26-O21)](24)+R[ν(CC)](21)+[ρ(C26=O20)](8) |
| 1311 | -- | [τ(C68HS1C)](27)+R1[δin(CH)](26)+[δ(S1OH)](9)+[τ(C68HS1O5)](8)+  [τ(C70HC26C)](5) |
| 1305 | -- | [δ(S1OH)](14)+[δ(C20C70C63)](12)+R4[δin(C50H)](11)+  R1[δin(C70H)](10)+R1[δtri](10)+[τ(C70HC26C)](8)+R2[δin(C60H)](5) |
| 1293 | -- | R[δin(CH)](42)+R[ν(CC)](18)+R[δin(C8C)](11)+R[δin(C18S)](11)+  [ρ’(S1O_3_)](7) |
| 1288 | -- | R1[δin(CH)](20)+R[δin(CH)](20)+[τ(C68HS1C)](17)+R[δin(C26C)](9)+  [τ(C70HC26C)](5)+R[ν(CC)](9)+R[δin(C36S)](5)+[τ(C68HS1O5)](5) |
| 1285 | -- | R1[ν(CC)](24)+[τ(C68HS1C)](13)+R1[δin(C68H)](9)+R1[ν(C67O)](5) |
| 1281 | 1275 | R1[δin(CH)](38)+[τ(C70HC26C)](28)+[τ(C68HS1C)](15)+  [τ(C68HS1O5)](5) |
| 1269 | 1267 | R1[δin(C70H)](48)+[τ(C70HC26C)](48) |
| 1244 | -- | R3[γ(C53H_2_)](20)+R3[ω(C53H_2_)](12)+R4[ν(C45C48)](8)+  [τ(C68HS1C)](7) |
| 1242 | 1246 | R1[δin(CH)](38)+[τ(C68HS1C)](24)+[τ(C70HC26C)](21)+  [τ(C68HS1O5)](6) |
| 1237 | 1234 | R3[γ(C56H_2_)](27)+R1[δin(CH)](15)+[τ(C68HS1C)](11)+  [τ(C70HC26C)](10)+[δ(C20C70C63)](5)+R2[ν(N38C60)](5) |
| 1230 | -- | [δ(C8O3H)](28)+R[ν(C8C)](23)+[ν(C8O3)]16)+R[δtri](7)+  R[ν(C9C10)](5) |
| 1215 | -- | R[δin(CH)](76)+R[ν(CC)](21) |
| 1215 | 1201 | R[δin(CH)](74)+R[ν(CC)](25) |
| 1211 | -- | [τ(C68HS1C)](23)+R1[δin(C68H)](12)+R4[δin(C45H)](7)+  [δ(C20C70C63)](7)+R3[ν(C49C59)](6)+[τ(C68HS1O5)](6) |
| 1184 | 1184 | R4[δtri](20)+R3[ν(C48C53)](17)+R4[δin(CH)](10)+[ρ’(C76H_3_)](8)+  R4[ν(C49C50)](6)+R5[ν(O41C52)](6) |
| 1179 | -- | R1[δin(CH)](29)+[τ(C68HS1C)](20)+[τ(C70HC26C)](17)+  [ρ’(C72H_3_)](10)+[ρ(C72H_3_)](7) |
| 1176 | -- | [τ(C70HC26C)](32)+R1[δin(C70H)](29)+[ρ’(C76H_3_)](13) |
| 1171 | -- | R[ν(CC)](22)+R[δin(CH)](19)+[ν(C8O3)]14)+R[δin(C18S)](8)+  R[δin(C8C)](7))+[δ(C8O3H)](5) |
| 1164 | 1166 | R[ν(CC)](27)+[ν(C26O20)](21)+R[δin(CH)](17)+[δ(C26OC70)](7)+  R[δtri](7)+R[δin(C36S)](5) |
| 1154 | -- | R1[δin(CH)](34)+ [τ(C70HC26C)](33)+[δ(C20C70C63)](5) |
| 1151 | -- | R5[γ(C39H_2_)](97) |
| 1149 | -- | [τ(C70HC26C)](21)+[ρ(C76H_3_)](20)+[τ(C68HS1C)](12)+  R1[δin(C70H)](9)+R1[δoop(C68H)](7)+[τ(C68HS1O5)](5) |
| 1147 | -- | R[ν(CC)](46)+R[δin(CH)](20)+R[δin(C26C)](12)+[ν(S19C)](6) |
| 1145 | 1143 | [ρ(C72H_3_)](39)+[ρ’(C72H_3_)](28)+[τ(C70HC26C)](12)+R1[δin(C70H)](8) |
| 1130 | 1132 | R1[δoop(CH)](41)+[τ(C70HC26C)](18)+[δ(C68HS1)](14)+  [τ(C68HS1C)](10)+[τ(C68HS1O5)](8) |
| 1126 | -- | R[ν(CC)](21)+R1[δoop(CH)](17)+[ν(C8O3)]13)+[τ(C70HC26C)](7)+  [δ(C68HS1)](6) |
| 1120 | 1114 | [τ(C70HC26C)](30)+R1[δin(CH)](27)+[τ(C68HS1C)](11) |
| 1095 | 1099 | R5[ρ(C39H_2_)](29)+[τ(C70HC26C)](23)+R1[δin(C70H)](16)+  [τ(C68HS1C)](6)+[δ(C20C70C63)](5) |
| 1094 | -- | [τ(C70HC26C)](30)+R1[δin(C70H)](21)+R5[ρ(C39H_2_)](7)+  [τ(C68HS1C)](7)+[δ(C20C70C63)](6) |
| 1092 | -- | R[δtri](58)+[ν(S19C)](13) |
| 1083 | -- | R[δtri](23)+[ν(S1O_3_)](21)+R[ν(C12C14)](7)+δ’as(S1O_3_)](5) |
| 1079 | 1079 | [ν(S1O_3_)](36)+R[δtri](21)+[δ’as(S1O_3_)](8)+[ν(S1C)](5)+  R1[δoop(C70H)](5) |
| 1073 | -- | R3[ρ(CH_2_)](37)+R3[ν(C53C56)](11)+R3[puck](6)+[τ(C68HS1C)](5) |
| 1073 | -- | R1[δoop(C68H)](22)+[δ(C68HS1)](17)+[ν(S19O_3_)](11)+R2[δoop(C64H)](11)+[τ(C68HS1C)](8)+[τ(C8O3)](7)+[τ(C68HS1O5)](6) |
| 1063 | 1064 | R1[δoop(C68H)](22)+R2[δoop(C64H)](19)+[δ(C68HS1)](17)+  [τ(C68HS1C)](9)+[τ(C68HS1O5)](8) |
| 1060 | -- | R[δoop(CH)](49+ R[puck](22) |
| 1059 | -- | R[δoop(CH)](44)+R[puck](15)+R[δtri](5)+R[ν(C12C14)](5) |
| 1058 | -- | [τ(S1O7)](49)+R2[δoop(C64H)](11) |
| 1051 | -- | R[δoop(CH)](25)+R[ν(CC)](16)+ R[puck](15)+[τ(S1O7)](5)+  R[ν(C10C12)](5) |
| 1048 | -- | R[ν(CC)](42)+R[puck](8)+R[δoop(CH)](15)+R[δin(CH)](9)+  [τ(C8O3)](7) |
| 1038 | -- | [τ(C70HC26C)](31)+R1[δin(CH)](18)+[ν(O47C76)](6) |
| 1031 | 1037 | R[δoop(CH)](76)+[R[τa’](9) |
| 1025 | -- | R[δoop(CH)](78)+R[τa’](9) |
| 1015 | 1016 | R5[ν(CO)](46)+R5[δa](10)+R4,R5[ν(C42C52)](7) |
| 1012 | 1002 | [τ(C8O3)](54)+[ν(S19O_3_)](31) |
| 999 | -- | [δ(C20C70C63)](14)+[τ(C68HS1C)](14)+R3[ν(C53C56)](12)+  [τ(C70HC26C)](11)+R1[δtri](8)+R[ν(O44C72)](6)+[τ(C68HS1O5)](5) |
| 989 | -- | R4[δoop(C50H)](79)+R4[puck](7) |
| 981 | 978 | R1[δoop(CH)](30)+[τ(C70HC26C)](17)+[δ(C68HS1)](13)+  R2[δoop(C60H)](10)+[τ(C68HS1C)](8)+[τ(C68HS1O5)](7)+R1[puck](5) |
| 967 | 967 | R1[δoop(CH)](36)+[τ(C70HC26C)](21)+[δ(C68HS1)](17)+  [τ(C68HS1C)](10)+[τ(C68HS1O5)](8) |
| 962 | -- | R1[δoop(CH)](31)+[δ(C68HS1)](13)+[τ(C70HC26C)](9)+  [τ(C68HS1C)](5)+[τ(S1OC68C67)](5)+[τ(C68HS1O5)](5) |
| 950 | 955 | R[δoop(CH)](50)+R[δoop(C26C)](16)+R[τa](8)+R[puck](8)+  R[δoop(C36S)](8) |
| 948 | -- | [τ(C70HC26C)](17)+[ν(O47C76)](16)+R1[δtri](11)+R1[δin(C70H)](6)+  [τ(S1OC68C67)](5) |
| 945 | -- | R[δoop(CH)](44)+R[δoop(C8C)](18)+R[τa](7)+R[puck](6) |
| 935 | 936 | R3[ρ(C53H_2_)](21)+R3[δtri](14)+R3[ρ(C56H_2_)](12)+[τ(C70HC26C)](6) |
| 923 | 921 | [ν(S1O6)](24)+[ν(S19O_3_)](19)+[τ(C68HS1C)](10)+[ρ’(S1O_3_)](8) |
| 918 | 913 | [ν(S19O_3_)](26)+[ν(S1O_3_)](21)+[τ(C68HS1C)](7)+[ρ’(S1O_3_)](6)+  [τ(C68HS1O5)](5) |
| 914 | 892 | R4[δoop(C45H)](71)+R4[puck](12) |
| 881 | 878 | [τ(C70HC26C)](29)+[τ(C68HS1C)](11)+R[ν(O44C72)](8)+  R1[δin(C70H)](5)+[δ(C20C70C63)](5)+R1,R2[ν(C62C63)](5) |
| 863 | 862 | R5[ν(O37C39)](37)+R5[ν(C39O41)](29)+R5[δ’a](9)+R5[ω(C39H_2_)](5) |
| 855 | 850 | R1[puck](58)+[τ(S1OC68C67)](10) |
| 836 | -- | R[δoop(C8C)](45)+R[puck](25)+[ω(C8=O2)](12)+  R[δoop(C14H)](6)+R[δoop(C18S)](5) |
| 835 | 832 | R[δoop(C26C)](28)+R[puck](19)+[ω(C26=O20)](13)+  [τ(C26OC70C63)](10)+[τ(C70HC26C)](7)+R[δoop(C36S)](7)+  [τ(C26OC70C68)](7) |
| 826 | -- | [δ(C20C70C63)](25)+[τ(C68HS1C)](11)+R5[ν(OC)](9)+R1[δa’](7)+  R4,R5[ν(C42C52)](7)+R4[ν(CC)](6)++R4[δa’](5)+R1[δtri](5) |
| 809 | 812 | R[δa](16)+R[ν(C8C)](14)+[ν(S1O7)](8)+[ρ(C8=O2)](7)+  [δ(S1OC68)](6)+R[δtri](6)+[ν(S1C)](5)+[δs(S1O_3_)](5) |
| 806 | -- | [δ(C26OC70)](20)+R[δa](15)+R[ν(C26C)](12)+[ρ(C26=O20)](10)+  [δ(C20C70C63)](6) |
| 802 | -- | R[δoop(CH)](33)+R[δoop(CS)](26)+R[puck](25) |
| 799 | 797 | R[δoop(CH)](40)+R[puck](28)+R[δoop(C18S)](12)+  R[δoop(C8C)](11) |
| 788 | -- | [δ(C20C70C63)](33)+R4[puck](17)+R1[δtri](8)+R1[δa’](7) |
| 787 | -- | [δ(C20C70C63)](38)+R4[puck](9)+R1[δtri](9)+R1[δa’](7) |
| 783 | 781 | [ν(S1O7)](20)+[δ(S1OC68)](16)+[τ(C68HS1C)](9)+[δs(S1O_3_)](7)+  [δ(C20C70C63)](5)+[τ(C70HC26C)](5)+R1[δin(C70H)](5) |
| 776 | 770 | R2[puck](16)+[τ(S1OC68C67)](13)+R1[puck](10)+[δ(C68HS1)](8)+  [δ(C20C70C63)](6)+[τ(C68HS1O5)](6)+[τ(C68HS1C)](6) |
| 758 | -- | [δ(C20C70C63)](14)+R1[δa](7)+R5[δa](6)+R3[ν(C48C53)](6)+  R4[δtri](5)+R2[δa’](5) |
| 757 | 752 | [ν(S19O_3_)](19)+[δ(C20C70C63)](16)+[δ(C26OC70)](12)+  [ν(S1O7)](6)+[ρ(C26=O20)](6)+[δ(S1OC68)](5) |
| 744 | -- | R3[ν(N38C56)](17)+R2,R3[ν(N38C59)](10)+[τ(C68HS1C)](7)+  R1[ν(C63C70)](6)+R2[δa](5)+R2[δa’](5)+R2[δtri](5) |
| 739 | 733 | [δ(C20C70C63)](15)+R2[δa’](14)+R1[δa](8)+R5[δa](6)+R4[δtri](5) |
| 720 | -- | R[puck](46)+[ω(C8=O2)](27)+R[δoop(CH)](7)+R[δoop(C18S)](6) |
| 719 | -- | [τ(C70HC26C)](15)+R5[δ’a](13)+[δ(C20C70C63)](9)+R4[τa](8)+  R3[δtri](6) |
| 716 | -- | [τ(C26OC70C63)](28)+[ω(C26=O20)](21)+[τ(C26OC70C68)](19)+  R[puck](17) |
| 712 | 714 | R5[δa](27)+R5[δ’a](14)+R5[ν(O37C39)](8)+R5[ν(C39O41)](6)+  R4[δa’](5) |
| 698 | -- | R[δa’](34)+[ρ(C8=O2)](17)+[ν(S1C)](9)+R[δa](5) |
| 691 | -- | R1[τa’](18)+[τ(C26OC70C63)](11)+[τ(C70HC26C)](7)+[δ(C68HS1)](6)+  [τ(C68HS1C)](5)+R1[τa](5)+[τ(S1OC68C67)](5)+RR1[δoop(C67O)](5) |
| 691 | 684 | R[δa’](33)+[ν(S19O_3_)](12)+[ν(S19C)](5) |
| 673 | -- | [δ(C20C70C63)](38)+[τ(C70HC26C)](22)+[τ(C68HS1C)](12) |
| 669 | 670 | R[δa’](32)+[ρ(C8=O2)](23)+R[δa](19)+[ρ’(S1O_3_)](8) |
| 665 | 660 | [ρ(C26=O20)](24)+[δ(C26OC70)](22)+[τ(C70HC26C)](15)+R[δa](8) |
| 646 | 645 | [τ(C70HC26C)](29)+[δ(C20C70C63)](15)+[τ(C68HS1C)](7)+  [τ(S1OC68C67)](6)+R1[τa’](5) |
| 607 | 614 | [τ(S1OC68C67)](26)+R1[τa](22)+[δ(C68HS1)](10)+  [τ(C26OC70C68)](8)+R1[δoop(C70H)](6) |
| 584 | 595 | [τ(C70HC26C)](11)+R1[τa](10)+R1[δa](7)+R3[δτtri](6)+  R4[τa](5)+[τ(C68HS1C)](5) |
| 574 | 574 | [δ(O21=C26C)](18)+R[δin(C26C)](15)+[τ(C70HC26C)](9)+  R[τa](7)+R[δoop(C36S)](5) |
| 569 | 565 | [δ(O2=C8C9)](15)+R[τa](14)+R[δoop(C8C)](11)+R[δin(C8C)](9)+  [ρ’(S1O_3_)](8)+[δ(O21=C26C)](6)+R[δin(C18S)](5) |
| 556 | -- | [τ(C70HC26C)](23)+R1[δa](16)+R3[δa](14)+[τ(S1OC68C67)](11)+  R1[δin(C70H)](6) |
| 551 | 554 | R[δoop(C36S)](20)+R[τa’](11)+R[τa](9)+R[δoop(C18S)](6)+  R[τa’](5)+R[τa](5) |
| 540 | -- | [τ(C70HC26C)](39)+[δ(C20C70C63)](12)+[τ(C68HS1C)](11)+R3[δa’](5)+R2[δa](5) |
| 536 | 535 | R[τa’](12)+R[δoop(C18S)](11)+[ρ’(S1O_3_)](11)+R[δoop(C36S)](9)+  R[τa’](5)+[δ(O2=C8C9)](5) |
| 521 | 523 | R1[δa’](29)+[τ(C68HS1C)](22)+[τ(C68HS1O5)](8)+[δ(C20C70C63)](6) |
| 507 | 506 | [δs(S1O_3_)](48)+[τ(C68HS1O5)](13) |
| 499 | -- | [δs(S1O_3_)](54)+[τ(C68HS1O5)](13) |
| 487 | -- | R2,R3[τ(C49CN53C)](13)+[τ(C68HS1C)](12)+R2[τa](10)+  [τ(S1OC68C67)](10)+R2[τa’](9)+R1[τa](7)+[τ(C68HS1O5)](6) |
| 472 | 476 | R[τa](14)+R[δoop(C26C)](11)+R[δoop(C8C)](8)+R[τa](7)+  [δs(S1O_3_)](7)+[δ’as(S19O_3_)](5)+[δ(C20C70C63)](5)+[ν(C20H71)](5) |
| 468 | -- | [δ(C20C70C63)](22)+[τ(C70HC26C)](22)+R4[τa’](7)+  [τ(S1OC68C67)](5)+R4,R5[τ(O41CCC)](5)+ |
| 462 | 460 | R[τa](18)+[τ(C26OC70C63)](18)+R[δoop(C26C)](15)+  [τ(C26OC70C68)](15)+R[τa’](8) |
| 459 | -- | R[τa](22)+R[δoop(C8C)](22)+R[τa’](8)+R[τ(C18S)](5) |
| 451 | -- | [τ(C70HC26C)](35)+[δ(C20C70C63)](15)+R4[δa](8)+R4[δa’](6)+  R1[δa](5) |
| 440 | 439 | [δ(C20C70C63)](21)+[τ(C70HC26C)](18)+[τ(C68HS1O5)](7)+  [τ(C26OC70C68)](7)+R1[δ(C67OC)](5) |
| 435 | -- | [δ’as(S1O_3_)](36)+[τ(S1OC68C67)](14)+[τ(C68HS1O5)](10)+  [δs(S1O_3_)](6) |
| 434 | 429 | [τ(C68HS1O5)](26)+[δas(S1O_3_)](17)+[δs(S1O_3_)](12)+[ρ’(S1O_3_)](5)+  R[τa](5) |
| 424 | -- | [τ(C26OC70C68)](32)+R1[δoop(CH)](13)+R1[puck](8)+  [τ(C68HS1O5)](7)+R1[τa](7)+[δ(C68HS1)](5) |
| 421 | -- | [ρ’(S1O_3_)](17)+[δas(S1O_3_)](13)+[τ(C68HS1C)](8)+R[τa’](7)+  [τ(C68HS1O5)](7)+[δas(S19O_3_)](6)+[δ(C20C70C63)](5) |
| 407 | 409 | [τ(C26OC70C68)](34)+R1[τa](11)+R1[δoop(C68H)](9)+  R1[δoop(C70H)](6)+R1[puck](5) |
| 397 | 394 | [δ’as(S1O_3_)](17)+[δ(C20C70C63)](16)+[τ(S1OC68C67)](12)+  [δas(S1O_3_)](7)+[δs(S1O_3_)](5)+[τ(C70HC26C)](5) |
| 395 | -- | [δ(C20C70C63)](17)+[τ(C68HS1O5)](14)+[τ(S1OC68C67)](14)+  [τ(C26OC70C63)](6)+[τ(C26OC70C68)](5)+[δ’as(S1O_3_)](5)+  [δ(C68HS1)](5) |
| 390 | 378 | [δas(S1O_3_)](34)+[δ’as(S1O_3_)](19)+[δs(S1O_3_)](7)+R[τa’](5)+  [τ(S1OC68C67)](5)+[τ(C68HS1O5)](5) |
| 359 | 357 | R1[δa’](24)+R1[δin(C66O)](7)+R1[δ(C66OC)]((6)+R1[δtri](5) |
| 350 | -- | [δ(C20C70C63)](21)+[τ(C70HC26C)](19)+[τ(S1OC68C67)](12)+  [δ(C68HS1)](5) |
| 340 | 338 | [ρ’(S1O_3_)](53)+[δ’as(S1O_3_)](9)+R[δin(C18S)](7)+[τ(C68HS1C)](5) |
| 319 | -- | [δ’as(S1O_3_)](12)+[ρ’(S1O_3_)](11)+[δ(C26OC70)](9)+R[δin(C26C)](9)+  [δ(C20C70C63)](8)+[τ(C26OC70C63)](6)+R[δin(C36S)](6)+  [δ’as(S19O_3_)](5)+[ρ(S19O_3_)](5) |
| 311 | -- | [τ(S1OC68C67)](40)+[τ(C68HS1O5)](8)+[δ(C68HS1)](7)+R1[τa](6) |
| 310 | -- | [τ(C70HC26C)](29)+[δ(C20C70C63)](14)+[τ(S1OC68C67)](8)+  [ρ’(S1O_3_)](8) |
| 302 | -- | [τ(C70HC26C)](30)+[δ(C20C70C63)](15)+[τ(C26OC70C68)](6)+  R1[τa](6)+R3[puck](5) |
| 291 | 290 | [τ(C68HS1O5)](25)+R[δin(C18S)](9)+[δas(S1O_3_)](7)+[ρ(S1O_3_)]6)+  [τ(S1OC68C67)](6)+[δs(S1O_3_)](6) |
| 287 | -- | [δ(C20C70C63)](25)+[τ(C70HC26C)](17)+[τ(C26OC70C68)](10)  +[τ(C68HS1C)](9)+[τ(C76O)](5) |
| 281 | -- | [δ(C20C70C63)](15)+[τ(C26OC70C63)](15)+[τ(C68HS1C)](13) |
| 275 | 278 | [δ(C20C70C63)](30)+[τ(C26OC70C68)](17)+[τ(C26OC70C63)](7) |
| 270 | -- | [τ(C68HS1O5)](21)+[ρ’(S1O_3_)](18)+[τ(S1OC68C67)](7)+  R[δoop(C8C)](6)+[ρ(S1O_3_)]6)+[δas(S1O_3_)](5) |
| 263 | -- | [τ(C26OC70C63)](13)+[δ(C20C70C63)](13)+[ρ’(S1O_3_)](12)+  [τ(S1OC68C67)](11)+[τ(C26OC70C68)](9)+[δ’as(S1O_3_)](6) |
| 249 | -- | [τ(C70HC26C)](31)+[δ(C20C70C63)](14)+R1[τa](8)+[τ(C68HS1C)](8) |
| 235 | 239 | [τ(S1OC68C67)](39)+[τ(C68HS1O5)](11)+[τ(C68HS1C)](9)+  [τ(C26OC70C63)](9)+R1[τa](8)+[δ(C68HS1)](5) |
| 225 | 222 | [τ(C26OC70C68)](21)+[τ(C26OC70C63)](13)+[τ(C70HC26C)](12)+  [τ(S1OC68C67)](7)+[δ(C26OC70)](6)+R[δin(C26C)](5)+  R[δoop(C26C)](5) |
| 218 | -- | [ρ’(S1OOO)](45)+[τ(S1OC68C67)](39) |
| 212 | -- | R1[τa](11)+R1[δ(C67OC)](10)+R1[δin(C67O)](9)+[δ(C20C70C63)](9)+  [τ(C76O)](8)+[τ(C68HS1C)](7)+[τ(C70HC26C)](5) |
| 207 | -- | [τ(C68HS1O5)](27)+[ρ’(S1OOO)](16)+[τ(C68HS1C)](11)+  [δ’as(S1OOO)](10)+R[δoop(C8C)](8) |
| 195 | 199 | [τ(C26OC70C68)](21)+[ν(C20H71)](7)+R1[τa’](7)+R1[τ(C67O)](6)  +[δ(C68HS1)](5) |
| 186 | -- | [τ(C68HS1O5)](13)+[δs(S1O_3_)](10)+R[τ(C18S)](10)+[δ’as(S1O_3_)](9)+  [ρ’(S1O_3_)](6)+[τ(S1OC68C67)](5)+R[τa](5)+R[δoop(C8C)](5)+  R[δin(C18S)](5) |
| 184 | -- | [τ(C26OC70C63)](32)+[τ(C26OC70C68)](27)+R[τ(C27C)](9)+  [δ(C26OC70)](6)+[δ’as(S1O_3_)](5) |
| 176 | -- | [τ(C70HC26C)](23)+[τ(S1OC68C67)](23)+[ρ’(S1O_3_)](8)+[τ(S1O7)](5)  +R[δin(C36S)](5)+R[τ(C27C)](5) |
| 170 | -- | [τ(S1OC68C67)](55)+[τ(C26OC70C68)](15) |
| 158 | -- | [τ(S1OC68C67)](39)+[δ(C20C70C63)](8) |
| 154 | 151 | [τ(C70HC26C)](14)+[δ’as(S1O_3_)](13)+R[τ(C18S)](10)+  R[τ(C36S)](9)+[τ(C68HS1O5)](6) |
| 147 | -- | [τ(S1OC68C67)](45)+[δ(C68HS1)](12)+[τ(C26OC70C63)](6)+  [τ(C68HS1C)](5)+[δ(C20C70C63)](5)+[τ(C68HS1O5)](5) |
| 146 | -- | [τ(C26OC70C63)](40)+[τ(C26OC70C68)](36)+R[τ(C27C)](5) |
| 133 | -- | [τ(C68HS1O5)](34)+R[τ(C18S)](19)+[ρ’(S1O_3_)](11)+R[τ(C9C)](10)  +[δs(S1O_3_)](5)+R[τa](5) |
| 128 | 129 | [τ(S1OC68C67)](36)+[τ(C26OC70C68)](23)+R1[τ(C67O)](5) |
| 126 | -- | [τ(C26OC70C68)](28)+[τ(S1OC68C67)](27)+  [τ(C26OC70C63)](22)+[ρ’(S1O_3_)](5) |
| 116 | -- | [δ(C20C70C63)](37)+[τ(C70HC26C)](28)+[τ(C68HS1C)](6)+  [τ(C26OC70C68)](6) |
| 113 | -- | [τ(S1OC68C67)](29)+[τ(C26OC70C63)](15)+[δ(C20C70C63)](11)+  [τ(C26OC70C68)](8)+[ρ’(S1O_3_)](5) |
| 109 | 107 | [τ(C26OC70C63)](17)+[τ(S1OC68C67)](17)+[τ(C26OC70C68)](15)+  [τ(C68HS1O5)](13)+R5[τa](8)+R5[τ’a](8) |
| 99 | -- | [τ(C26OC70C63)](18)+[τ(S1OC68C67)](15)+[τ(C70HC26C)](9)+  [ρ’(S1O_3_)](7)+[τ(C26OC70C68)](5) |
| 94 | -- | [τ(S1OC68C67)](59)+[τ(C26OC70C68)](24) |
| 90 | -- | [δ(C20C70C63)](25)+[τ(C70HC26C)](17)+[τ(S1OC68C67)](16)+  [τ(C68HS1C)](10)+[δ(C26OC70)](8)+[τ(C26OC70C63)](5) |
| 81 | -- | [ρ’(S1O_3_)](27)+[τ(C26OC70C63)](19)+[δ(C68HS1)](9)+  [τ(S1OC68C67)](7)+[τ(C26OC70C68)](6)+[δ(S1OC68)](5) |
| 78 | -- | [τ(C68HS1O5)](26)+[δ(C20C70C63)](14)+[τ(C68HS1C)](12)+  R[τ(C18S)](11) |
| 74 | -- | [δ(C20C70C63)](52)+[τ(C70HC26C)](27)+[τ(S1OC68C67)](7)+  [δ(C26OC70)](6) |
| 63 | -- | [τ(C68HS1C)](23)+[τ(S1OC68C67)](21)+[δ(C26OC70)](12)+  [τ(C70HC26C)](9)+R[τ(C18S)](8)+[ν(C20H71)](6) |
| 61 | -- | [τ(C68HS1O5)](21)+[τ(C26OC70C68)](17)+[τ(C26OC70C63)](16)+  [τ(S1OC68C67)](15)+[δ(C68HS1)](8)+R[τ(C18S)](7) |
| 51 | -- | [τ(S1OC68C67)](47)+[τ(C26OC70C68)](20)+[δ(C26OC70)](7) |
| 47 | -- | [τ(C68HS1O5)](34)+[δ(C20C70C63)](16)+[τ(S1OC68C67)](13)+  [τ(C26OC70C68)](8)+[τ(C68HS1C)](6) |
| 35 | -- | [δ(C20C70C63)](56)+[τ(C70HC26C)](23)+[ν(C20H71)](5) |
| 32 | -- | [τ(C68HS1O5)](45)+[τ(C70HC26C)](15)+[δ(C20C70C63)](11)+  [τ(S1OC68C67)](10)+[δ(C68HS1)](6) |
| 27 | -- | [τ(C70HC26C)](38)+[τ(C26OC70C63)](29)+[τ(C26OC70C68)](18)+  [τ(C68HS1C)](6) |
| 23 | -- | [δ(C20C70C63)](27)+[τ(S1OC68C67)](26)+[τ(C68HS1C)](25)+  [τ(C70HC26C)](7) |
| 12 | -- | [τ(C68HS1O5)](23)+[τ(C70HC26C)](23)+[τ(C68HS1C)](16)+  [τ(C26OC70C63)](13)+[τ(S1OC68C67)](10)+[δ(C68HS1)](6) |
| 11 | -- | [τ(C26OC70C63)](53)+[δ(C68HS1)](13)+[τ(C26OC70C68)](13)+  [τ(S1OC68C67)](9)+[δ(C20C70C63)](5) |

**Table S7.** Geometrical parameters for intermolecular interaction in BER-SBA salt: bond distance (Å), bond angle (°) and sum of van der Waals radii of interacting atoms (Å).

| **Interactions (D−H···A)** | ***d*_D−H_** | ***d*_H···A_** | ***d*_D···A_** | **D−H···A** | **(*r*_H_+*r*_A_)** |
| --- | --- | --- | --- | --- | --- |
| S1-O7···H71 | 1.6940 | 2.5117 | 3.9128 | 136.1008 | 2.72 |
| S1-O6···H17 | 1.6006 | 2.1957 | 2.7115 | 89.7498 | 2.72 |
| O3-H4···O25 | 1.0424 | 1.5347 | 2.5670 | 169.6949 | 2.72 |
| C26=O20···H71 | 1.3099 | 2.0825 | 3.0490 | 126.4817 | 2.72 |
| S1-O7···H22 | 1.6940 | 1.0810 | 2.3856 | 116.8283 | 2.72 |
| S19-O24···H35 | 1.5861 | 2.3533 | 2.7695 | 87.0544 | 2.72 |
| S19-O23···H51 | 1.6900 | 2.1685 | 3.2032 | 111.6354 | 2.72 |
| S19-O23···H65 | 1.6900 | 2.1630 | 3.5072 | 130.6880 | 2.72 |
| C67-O47···H73 | 1.3752 | 2.1363 | 2.8018 | 103.7337 | 2.72 |
| S1-O6···H79 | 1.6006 | 2.2294 | 3.3945 | 123.9948 | 2.72 |
| S1-O7···H69 | 1.6940 | 2.0905 | 3.0044 | 104.6130 | 2.72 |

**Table S8.** Second-order perturbation theory analysis of the Fock matrix, in the NBO basis for intra- and intermolecular interactions in BER-SBA salt.

| **Donor Orbitals** | **Donor Occupancy** | **Acceptor Orbitals** | **Acceptor Occupancy** | **E(2)**  **kcal/mol** |
| --- | --- | --- | --- | --- |
| **Within unit 1** | | | | |
| π(C9−C10) | 0.98741 | π*(C12−C14) | 0.16156 | 10.08 |
| π(C9−C10) | 0.81140 | π*(C16−C18) | 0.17541 | 10.37 |
| π(C12−C14) | 0.82220 | π*(C9−C10) | 0.17010 | 10.33 |
| π(C12−C14) | 0.82220 | π*(C16−C18) | 0.17541 | 11.25 |
| π(C16−C18) | 0.83609 | π*(C9−C10) | 0.17541 | 10.50 |
| LP (2) O2 | 0.92674 | σ*(O3−C8) | 0.04198 | 14.35 |
| LP (2) O3 | 0.88898 | π*(O2−C8) | 0.13548 | 27.31 |
| LP (3) O5 | 0.88997 | σ*(S1−O7) | 0.14819 | 12.52 |
| LP (3) O6 | 0.90444 | σ*(S1−O7) | 0.14819 | 9.80 |
| **From unit 1 to 2** | | | | |
| LP (1) O2 | 0.98638 | σ(O21−C26) | 0.01943 | 0.19 |
| LP (2) O2 | 0.92674 | π*(O21−C26) | 0.17572 | 0.31 |
| LP (3) O7 | 0.83664 | LP*(1) H 22 | 0.22476 | 148.01 |
| **From unit 1 to 3** | | | | |
| LP (3) O6 | 0.90444 | σ*(C76−H79) | 0.01229 | 1.24 |
| LP (1) O7 | 0.97525 | σ*(C68−H69) | 0.01185 | 2.02 |
| **Within unit 2** | | | | |
| π(C27−C28) | 0.80980 | π*(O21−C26) | 0.17572 | 11.34 |
| π(C27−C28) | 0.80980 | π*(C34−C36) | 0.17642 | 10.98 |
| π(C30−C32) | 0.81794 | π*(C27−C28) | 0.16866 | 10.66 |
| π(C30−C32) | 0.81794 | π*(C34−C36) | 0.17642 | 11.09 |
| π(C34−C36) | 0.83895 | π*(C27−C28) | 0.16866 | 10.10 |
| LP (3) O20 | 0.85382 | π*(O21−C26) | 0.17572 | 36.64 |
| LP (3) O24 | 0.90167 | σ*(S19−O23) | 0.13520 | 11.32 |
| **From unit 2 to 1** | | | | |
| LP (1) O25 | 0.97194 | LP*(1) H4 | 0.22783 | 10.70 |
| LP (3) O25 | 0.90198 | LP*(1) H4 | 0.22783 | 23.07 |
| π(O21−C26) | 0.98971 | LP*(1) H22 | 0.22476 | 1.30 |
| LP (1) O21 | 0.97496 | LP*(1) H22 | 0.22476 | 6.42 |
| **Unit 2 to unit 3** | | | | |
| LP (1) O20 | 0.97496 | σ*(C70−H71) | 0.01412 | 1.43 |
| LP (2) O20 | 0.90969 | σ*(C70−H71) | 0.01412 | 2.36 |
| LP (1) O23 | 0.99031 | σ*(C64−H65) | 0.01195 | 1.23 |
| LP (2) O23 | 0.96118 | σ*(C50−H51) | 0.01280 | 2.05 |
| LP (3) O23 | 0.95136 | σ*(C64−H65) | 0.01195 | 1.72 |
| **from unit 3 to unit 1** | | | | |
| σ(C68−H69) | 0.98571 | σ*(S1−O7) | 0.14819 | 0.07 |
| σ(C76−H79) | 0.99737 | σ*(S1−O6) | 0.08624 | 0.06 |
| **Within unit 3** | | | | |
| π(C42−C45) | 0.84137 | π*(C48−C49) | 0.20976 | 10.48 |
| π(C48−C49) | 0.84146 | π*(N38−C59) | 0.36257 | 12.12 |
| π(C50−C52) | 0.84530 | π*(C42−C45) | 0.18587 | 10.46 |
| LP (2) O37 | 0.92548 | π*(C42−C45) | 0.18587 | 13.80 |
| LP (2) O41 | 0.92894 | π*(C50−C52) | 0.16520 | 13.14 |
| LP (2) O47 | 0.91876 | π*(C66−C67) | 0.18208 | 14.67 |

**Table S9** Molecular docking analysis showing the binding mode and intermolecular interactions of berberine with selected proteins.

| **Ligand** | **Target** | **PDB code** | **Binding Energy**  **Kcal mol^-1^** | **Ligand Efficiency** | **Hydrogen bonds** | **Bonded residues** | **Hydrogen bond lengths (Å)** |
| --- | --- | --- | --- | --- | --- | --- | --- |
| Berberine | HMG-CoA (3-hydroxy-3-methylglutaryl-coenzyme A) | 1T02 | -5.79 | -0.23 | 3 | ALA B:331  GLY B:330  ASP B:283 | 3.31  3.13  3.05 |
|  | MAPK (Mitogen-activated protein kinase ) | 1HZM | -7.14 | -0.29 | 2 | CYS A:147  GLU A:148 | 1.94  2.16 |
|  | Nrf2 (Nuclear factor erythroid 2-related factor ) | 7K2F | -9.28 | -0.39 | 2 | ARG B:470  HIS B:516 | 2.63  2.66 |
